# Supplementary material for: L-RNA aptamer-based CXCL12 inhibition combined with radiotherapy in newly-diagnosed glioblastoma: dose escalation of the phase I/II GLORIA trial
Source: Nat Commun. 2024 May 28;15:4210. doi: 10.1038/s41467-024-48416-9 (PMC11133480; doi:10.1038/s41467-024-48416-9)
Supplement: Supplementary file 1 — Supplementary Information [file 41467_2024_48416_MOESM1_ESM.pdf]

Supplementary Information

a

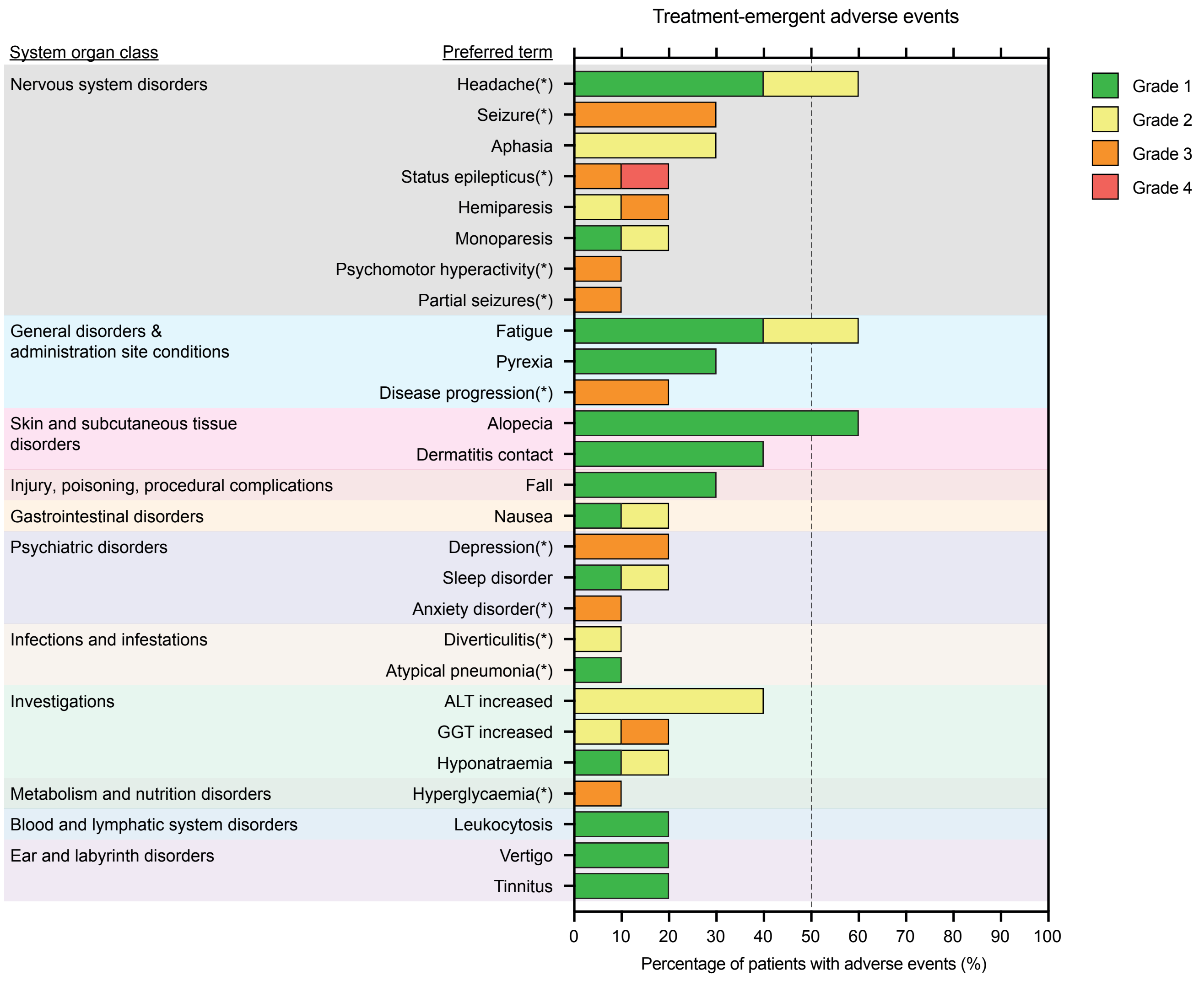

b

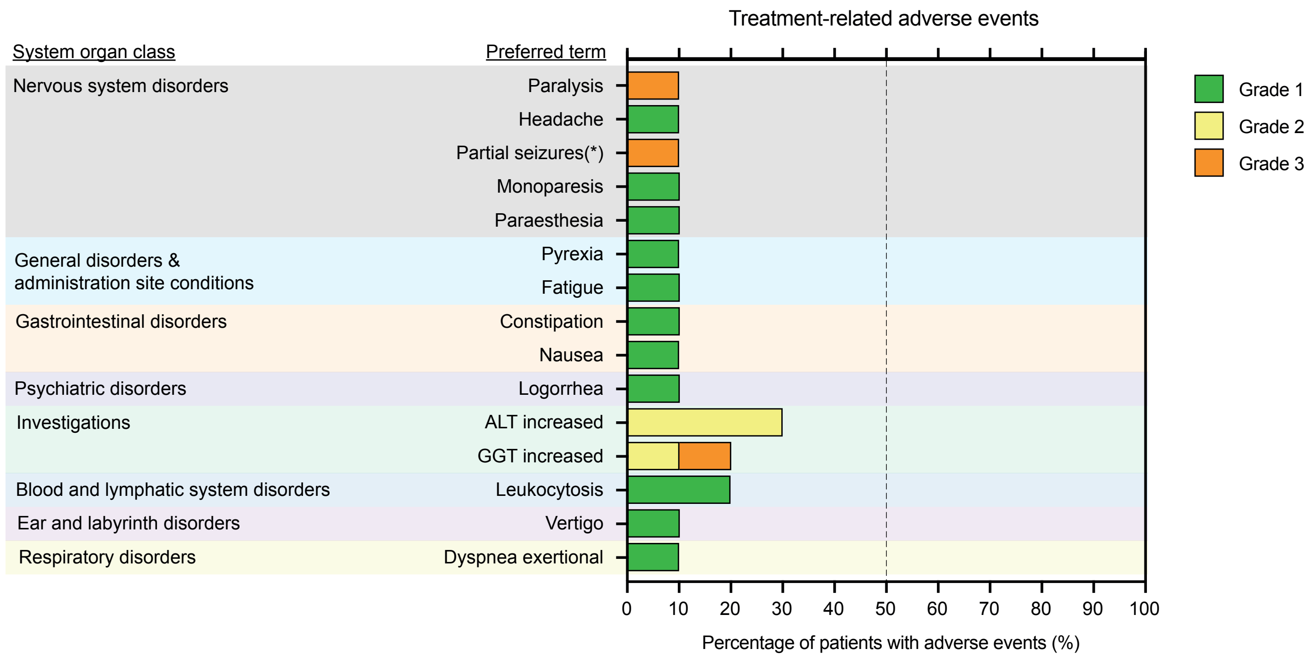

Supplementary Fig. 1: Treatment-emergent AEs (TEAEs) and treatment-related AEs (TRAEs) in the GLORIA trial. a, Bar plots depicting percentage of affected GLORIA patients (n=10) and gradings as per CTCAE for all TEAEs affecting at least two patients or having been reported as serious adverse event (SAE, indicated by an asterisk (*)). **b,** Bar plots depicting percentage of affected GLORIA patients (n=10) and gradings for all TRAEs. SAEs are indicated additionally by an asterisk (*). TRAEs are defined as TEAEs related to NOX-A12 and/or underlying disease and/or irradiation. ALT: alanine aminotransferase; CTCAE: Common Terminology Criteria for Adverse Events; GGT: gamma-glutamyltransferase.

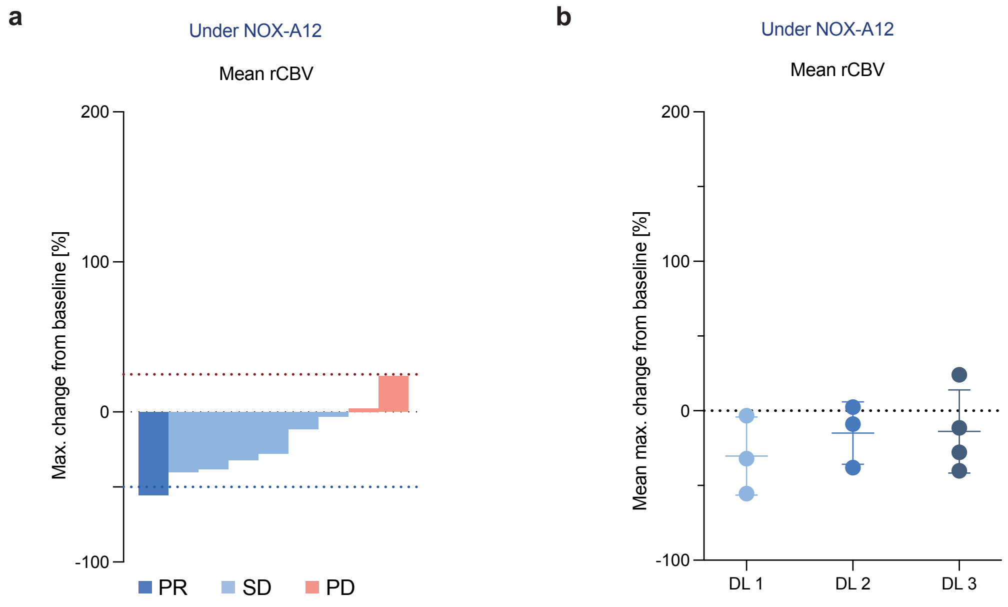

Supplementary Fig. 2: Treatment with RT and NOX-A12 decreases relative cerebral blood volume (rCBV). a, Waterfall plot for individual best rCBV response under NOX-A12 (maximum change from baseline). Colors from blue to red indicate PR, SD and PD for each patient. Red dotted line indicates 25% increase (PD), blue dotted line indicates -50% decrease (PR). **b,** Dot plots depicting mean maximum change from baseline under NOX-A12 for rCBV in the respective DLs (color-labeling in blue; 200 (n=3), 400 (n=3), 600 mg/week (n=4)). Error bars indicate mean and standard deviation. DL: dose level; NOX-A12: olaptesed pegol; PD: progressive disease; PR: partial response; rCBV: relative cerebral blood volume; SD: stable disease.

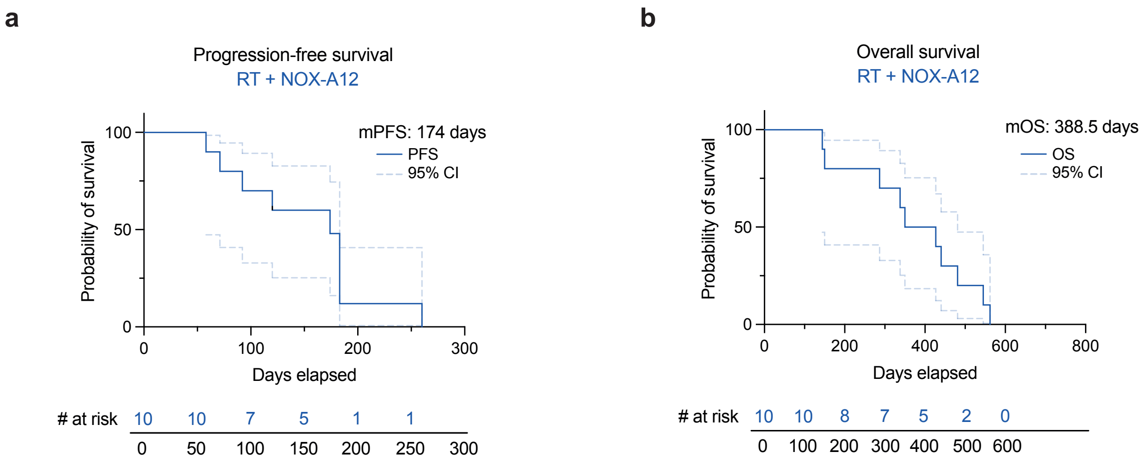

Supplementary Fig. 3: Survival outcome of the GLORIA cohort. Kaplan-Meier curves of progression-free (**a**) and overall (**b**) survival (in days) in the GLORIA cohort (n=10). Dashed lines indicate 95% confidence intervals. mOS: median overall survival; mPFS: median progression-free survival.

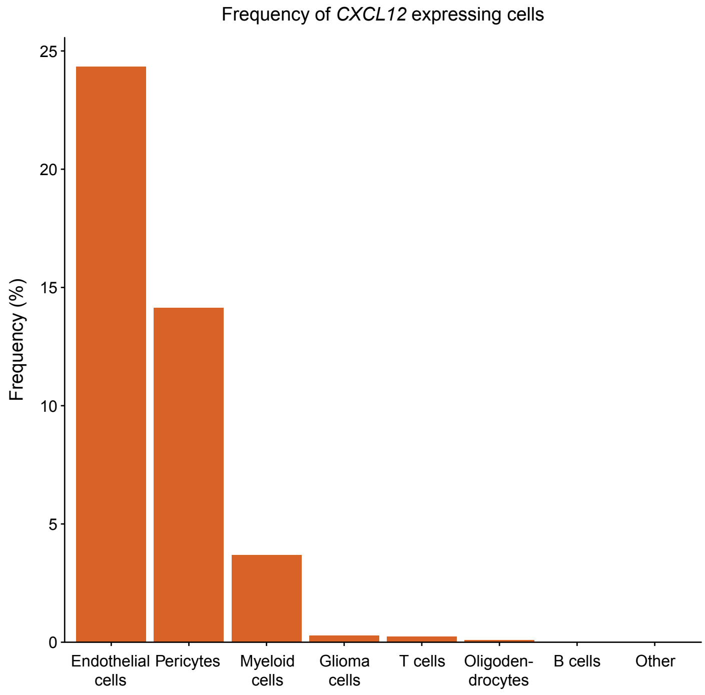

Supplementary Fig. 4: Frequencies of CXCL12 expressing cells within clusters of analyzed cells from Abdelfattah et al., 2022. Bar plot of the frequency of cells (%) within each cluster that is CXCL12 positive (≥ 1 log-normalized count).

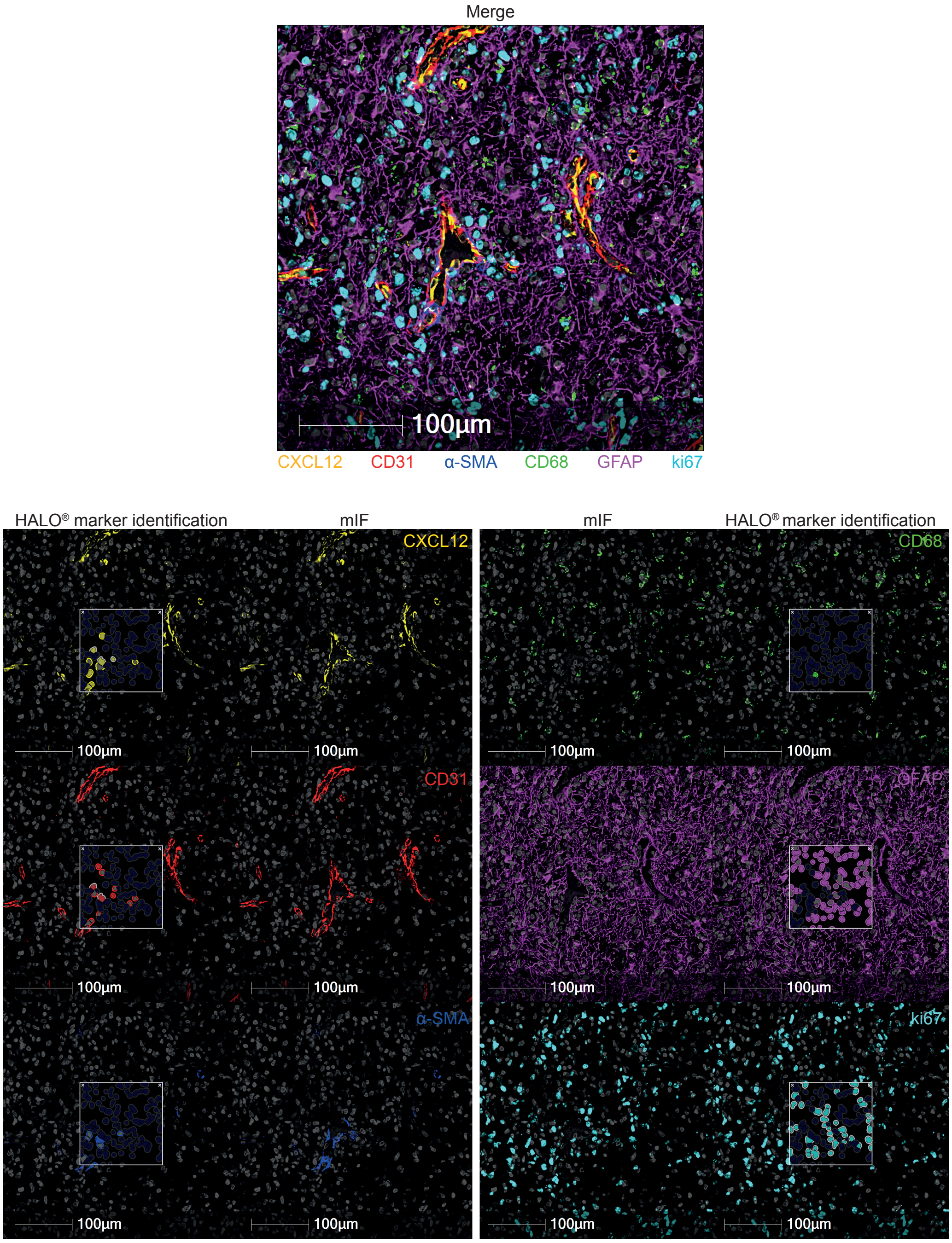

Supplementary Fig. 5: Representative multiplexed immunofluorescence image field of a GBM section and marker identification. Exemplary images (n=32) show mIF staining of cell types of interest (GBM tissue sample from GLORIA patient C1-001) and the identification of the corresponding phenotype by the algorithm implemented within the HALO® software. Top panel: Merged CXCL12/CD68/CD31/GFAP/α-SMA/Ki-67/DAPI mIF staining; lower panels: individual channels; CXCL12 (yellow), CD68 (green), CD31 (red), GFAP (magenta), α-SMA (blue), Ki-67 (cyan), DAPI (gray). GBM: glioblastoma; mIF: multiplexed immunofluorescence.

Supplementary Fig. 6: Side-by-side illustration of tumor areas analyzed in mIF staining and corresponding H&E staining. mIF analysis (left panels) of GLORIA and SOC cohort samples was performed in the indicated tumor areas (yellow) as identified by two independent neuropathologists in the corresponding H&E staining (right panels). Excluded areas were further characterized as follows. A: artefact; B: bleeding; G: granulocytes; LM: leptomeninges; mIF: multiplexed immunofluorescence; N: necrosis.

High resolution images of Supplementary Fig. 6 are provided in the following repository:

Layer, Julian (2024), “Giordano, Layer, Leonardelli et al. Supplementary Fig. 6”, Mendeley Data, V1, doi: 10.17632/wfhnv7j2wh.1

https://data.mendeley.com/datasets/wfhnv7j2wh/1

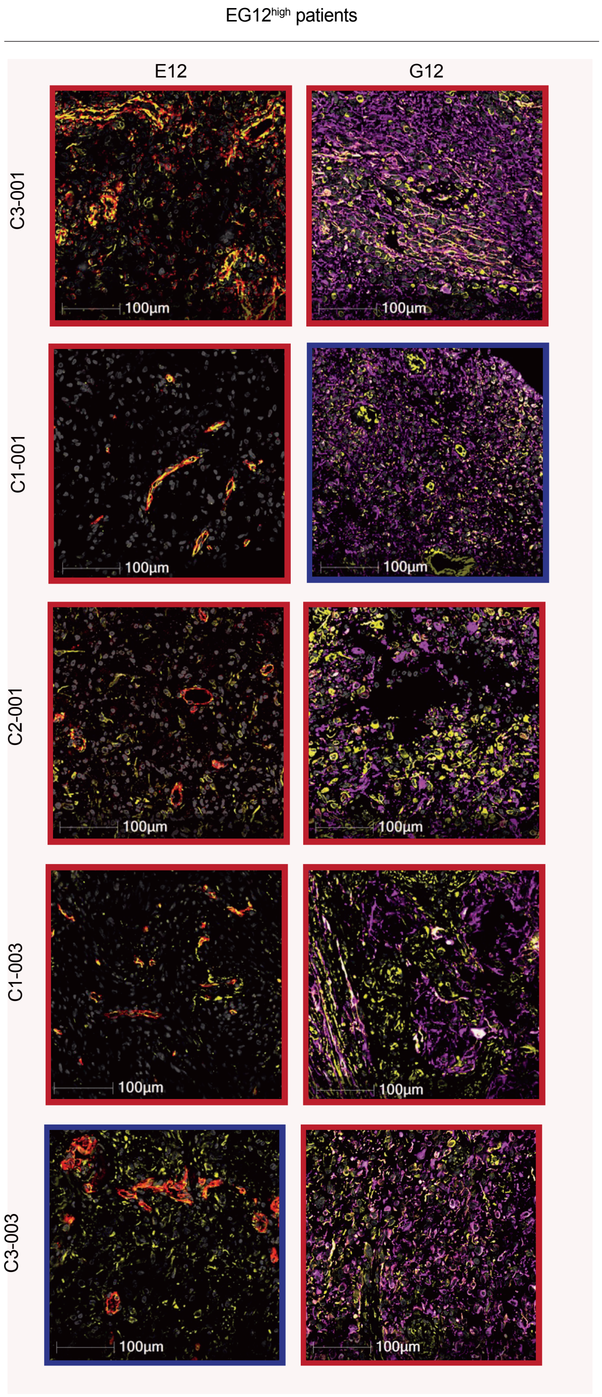

Supplementary Fig. 6: Patient stratification by the combined EG12 score compared to isolated E12 and G12 assessment. Representative mIF image sections of EG12^{high} GLORIA patients showing concordant (E12^{high} and G12^{high}) or discordant E12 and G12 (E12^{high}/G12^{low} or E12^{low}/G12^{high}) as indicated by color coding of image borders (red: high; blue: low). E12: CXCL12+ endothelial cells; G12: CXCL12+ glioma cells; mIF: multiplexed immunofluorescence.

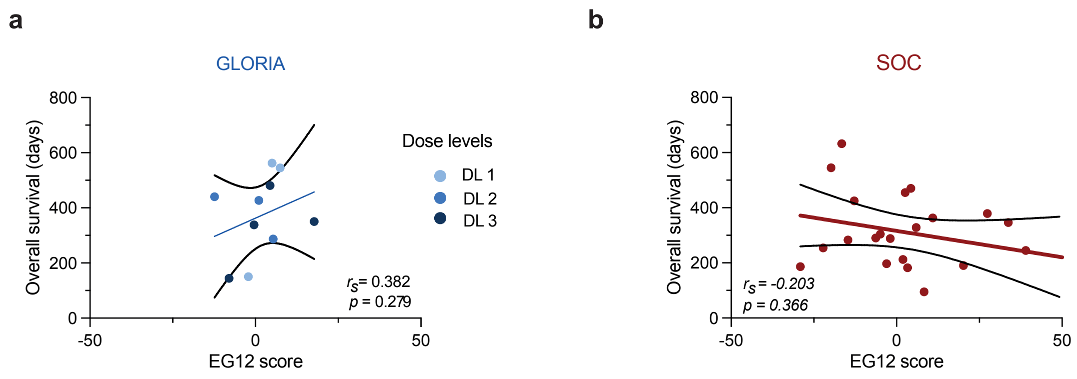

Supplementary Fig. 7: Correlation of EG12 with overall survival in the GLORIA and SOC cohort. Correlation analysis of overall survival (days) with EG12 score in corresponding tumor tissue of the GLORIA cohort (left panel, n=10) and the SOC cohort (right panel, n=22). Spearman's rank correlation; r_s- and *p*-values (two-sided) are depicted in the corresponding graphs.

Supplementary Note 1: Patient narratives of the GLORIA cohort

C1-001

This is a patient with a frontal lobe GBM. The patient underwent incomplete resection on 28.08.2019. After proper diagnosis, the patient was included in GLORIA. The patient was started on 200mg/week NOX-A12 on October 7, 2019 and was treated with normofractionated RT. A non-planned scan was performed on December 10, 2019 (week 11) due to worsening clinical status. MRI/ RANO suggested preliminary PD. Since this was the first patient on NOX-A12 and there was no experience with the drug, the confirmatory scan was skipped and the patient was scheduled for surgery (performed on January 3, 2020) and treatment was interrupted for three days from January 8 to 10, 2020. However, histopathology was indicative of reactive tissue only with no proof of tumor recurrence. Thus, the situation was interpreted as surgery for symptomatic pseudoprogression/radionecrosis and not for PD, defining the time point response to be "stable disease". Thus, an AE grade 3 event *radionecrosis* was documented and the patient continued with NOX-A12. The patient remained clinically unimpaired (and physically active). Therapy was discontinued as per protocol after 26 weeks (April 6, 2020), where an MRI confirmed stable disease. This long period of freedom of recurrence corroborated the definition of the occurrence of pseudoprogression instead of tumor recurrence. Three months after end of treatment (June 22, 2020) an MRI suggested tumor recurrence (pPD), which was then confirmed in a subsequent scan. At this stage, the patient personally decided to refuse any further intervention or therapy. Therefore, no further therapies after the 26 weeks of NOX-A12 were applied. The patient deceased on April 3, 2021.

Final interpretation:

- PD as per RANO occurred June 22, 2020 with a resulting PFS of **260 days.**
- The overall survival time was **545 days**

C1-002

This is a patient with a frontoparietal GBM. The patient underwent biopsy on January 15, 2020. After proper diagnosis, the patient was included in GLORIA. The patient was started on 200mg/week NOX-A12 on February 10, 2020 and was treated with hypofractionated RT. The first scan in week 9 showed stable disease. An unplanned scan was performed on May 11, 2020 (week 14) due to worsening neurological symptoms requiring high dose dexamethasone treatment, but MRI (RANO) revealed SD with even decreased tumor size. Therefore, NOX-A12 was continued. However, the patient repeatedly suffered from seizures with the worst occurring on May 26, 2020 (grade 4). On June 8, 2020 an MRI suggested tumor recurrence (pPD), but the patient died on July 8, 2020 before a confirmatory scan could be performed. Our interpretation is that even in the absence of a confirmatory MRI scan, PD as observed with clinical deterioration to no other cause but the tumor was most likely already present on May 11, 2020 and confirmed by the follow-up scan on June 8, 2020. Thus, the MRI on May 11, 2020 is the event for PFS.

Final interpretation:

- PD as per RANO occurred May 11, 2020 with a resulting PFS of **92 days**
- The overall survival time was **150 days**

C1-003

This is a patient with a left frontal GBM. The patient underwent partial resection on February 26, 2020. After proper diagnosis, the patient was included in GLORIA. The patient was started on 200mg/week NOX-A12 on March 25, 2020 and was treated with normofractionated RT. The first scan in week 9 showed partial remission (PR), which was confirmed in the week 18 scan. The patient completed 26 weeks of NOX-A12 per protocol. At the time of completion of NOX-A12, an MRI performed on September 23, 2020 suggested tumor recurrence (pPD) which was then confirmed in a scan on November 5, 2020. The patient received various other therapies (temozolomide, regorafenib, CCNU, bevacizumab) and subsequently died on October 7, 2021.

Final interpretation:

- PD as per RANO occurred September 23, 2020 with a resulting PFS of **183 days**
- The overall survival time was **562 days**

C2-001

This is a patient with a frontotemporal GBM. The patient underwent partial resection on May 22, 2020. After proper diagnosis, the patient was included in GLORIA. The patient was started on 400 mg/week NOX-A12 on June 22, 2020 and was treated with hypofractionated RT. The first scan in week 9 showed stable disease. The scan performed on October 19, 2020 (week 18) suggested tumor recurrence (pPD) which was not confirmed in a further scan. Of note, at the time point of this MRI no AEs were reported and the time point NANO score remained 0. Despite this, the PI decided to discontinue NOX-A12 and initiate salvage therapy with temozolomide on November 18, 2020 without performing an MRI scan. On January 6, 2021 the patient received an infusion of bevacizumab. Although a scan performed 3 weeks later showed PR, while previous pseudo-progression cannot be excluded, we considered the overall time point response as “not evaluable” due to “bevacizumab blinding”. After rapid clinical deterioration, the patient died on April 4, 2021.

Since salvage therapy was i) initiated in the absence of confirmed PD and ii) no clinical deterioration was noted at the previous scan date, the patient is censored for PFS at the time point of the scan preceeding the initiation of salvage therapy (October 19, 2020).

Final interpretation:

- PD as per RANO is indeterminable, resulting in PFS being censored at **120 days**
- The overall survival time was **287 days**

C2-002

This is a patient with a temporoparietal GBM. The patient underwent partial resection on August 31, 2020. After proper diagnosis, the patient was included in GLORIA. The patient was started on 400 mg/week NOX-A12 on September 28, 2020 and was treated with normofractionated RT. The first scan (week 9) on November 24, 2020 suggested tumor recurrence (pPD) which was confirmed in an unscheduled scan on December 22, 2020, where also clinical deterioration was noted. NOX-A12 was discontinued and the patient was started on various other therapies (temozolomide, bevacizumab). The patient died on December 11, 2021.

Final interpretation:

- PD as per RANO occurred November 24, 2020 with a resulting PFS of **58 days**
- The overall survival time was **440 days**

C2-003

This is a patient with a temporal GBM. The patient underwent biopsy on September 4, 2020. After proper diagnosis, the patient was included in GLORIA. The patient was started on 400 mg/week NOX-A12 on October 5, 2020 and was treated with normofractionated RT. The first scan (week 9) was defined to be stable disease. The scan on February 1, 2021 (week 18) was suggestive for tumor recurrence (pPD). The patient also showed a worsening clinical condition (increasing aphasia) thus treatment with NOX-A12 was ended. However, the confirmatory scan on March 4, 2021 was interpreted by the central reader as SD, thus the patient was formally not reaching confirmed radiographic PD. The patient decided anyhow to discontinue the treatment under clinical deterioration, salvage therapy with temozolomide was initiated on February 23, 2021. Because of accompanying clinical deterioration, the scan on February 1, 2021 was in overall assessment defined as date of PD. The patient was later started on various other therapies (temozolomide, bevacizumab, CCNU). The patient died on December 5, 2021.

Final interpretation:

- PD as per RANO occurred February 1, 2021 with a resulting PFS of **120 days**
- The overall survival time was **427 days**

C3-001

This is a patient with a temporal GBM. The patient underwent partial resection on January 8, 2021. After proper diagnosis, the patient was included in GLORIA. The patient was started on 600 mg/week NOX-A12 on February 8, 2021 and was treated with hypofractionated RT. The first two scans (week 7 and 18) were defined to be stable disease. A scan on August 9, 2021 (week 27) was suggestive for tumor recurrence (pPD). The patient showed no worsening clinical condition. NOX-A12 was discontinued as the site was hesitant to treat beyond 26 weeks. After discussion with the patient and shared decision-making, NOX-A12 was re-initiated on August 23, 2021. The scan on September 3, 2021 showed confirmed PD. The patient then underwent re-surgery on September 6, 2021, where vital tumor was found, confirming PD. Despite no conformation MRI scan was carried out, we thus considered the initial MRI suggesting PD to be the date of PD (August 9, 2021). The patient received various salvage therapies (Re-RT, temozolomide, CCNU) but eventually died on January 23, 2022.

Final interpretation:

- PD as per RANO occurred on August 9, 2021 with a resulting PFS of **183 days**
- The overall survival time was **350 days**

C3-002

This is a patient with a parieto-occipital GBM. The patient underwent partial resection on February 12, 2021. After proper diagnosis, the patient was included in GLORIA. The patient was started on 600 mg/week NOX-A12 on March 24, 2021 and was treated with normofractionated RT. An unscheduled scan was performed on April 29, 2021 due to clinical deterioration. This scan showed a reduction of enhancing tumor size (considered pPR) but there was still a considerable edema, so the patients was put on steroids. The patient improved clinically and a further scan two weeks later on May 19, 2021 (week 9) then confirmed PR and a shrinking edema. There were various treatment interruptions due to unclear elevation of liver enzymes (ALT), which was potentially caused by a sigma diverticulitis diagnosed in May 2021. A further unscheduled MRI scan on June 2, 2021 showed SD while clinical deterioration due to the tumor was noted by the PI. On June 24, 2021 another unscheduled scan was suggestive for tumor recurrence (pPD) and NOX-A12 was discontinued. The patient then refused any further scan and intervention and died on August 14, 2021. The patient had as part of the highest dose level cohort only reveived treatment for a short period and with various treatment interruptions. While the patient remained part of the intention-to-treat cohort, the patient was therefore considered a drop-out and replaced to ensure data quality of the primary endpoint (safety).

Despite the scan on June 2, 2021 was not confirmed, we think that rapid tumor progression caused the death 2.5 months later and thus this scan is the date of PD.

Final interpretation:

- PD as per RANO occurred on June 2, 2021 with a resulting PFS of **71 days**
- The overall survival time was **144 days**

C3-003

This is a patient with a frontal lobe GBM. The patient underwent partial resection on February 17, 2021. After proper diagnosis, the patient was included in GLORIA. The patient was started on 600 mg/week NOX-A12 on March 31, 2021 and was treated with normofractionated RT. The patient had an uneventful course with all scans showing SD in week 9 and 18. A scan on September 20, 2021 (week 26) was suggestive for tumor recurrence (pPD) and the patient also deteriorated clinically. NOX-A12 was discontinued. The patient underwent subsequent therapies (Re-RT, temozolomide, bevacizumab). The follow-up scan on November 15, 2021 confirmed PD. The patient eventually died on July 24, 2022.

Final interpretation:

- PD as per RANO occurred on September 20, 2021 with a resulting PFS of **174 days**
- The overall survival time was **481 days**

C3-004

This is a patient with a frontotemporal GBM. The patient underwent partial resection on August 9, 2021. After proper diagnosis, the patient was included in GLORIA. The patient was started on 600 mg/week NOX-A12 on September 13, 2021 and was treated with normofractionated RT. The patient had an uneventful course with SD on week 9. A scan on January 10, 2022 (week 18) showed even PR. However, a scan on March 14, 2022 (week 27) was suggestive for tumor recurrence (pPD) but the patient remained clinically stable, thus, NOX-A12 was not discontinued. The confirmation scan on April 12, 2022 confirmed PD and the patient received additional bevacizumab. The patient died on August 16, 2022.

Final interpretation:

- PD as per RANO occurred on March 14, 2022 with a resulting PFS of **183 days**
- The overall survival time was **338 days**

Supplementary Note 2: GLORIA study protocol, minimally redacted to account for data protection rights of the sponsor.

NOXXON
PHARMA AG

Clinical Study Protocol

Single-arm, Dose-Escalation, Phase 1/2 Study of Olaptesed Pegol (NOX-A12) in Combination with Irradiation in Inoperable or Partially Resected First-line Glioblastoma Patients with Unmethylated MGMT Promoter with a 3-arm Expansion Group Including Fully Resected Patients and Combination with Bevacizumab or Pembrolizumab

**Study Protocol Number:**	SNOXA12C401
**Short study title:**	GLIOBLASTOMA TREATMENT WITH IRRADIATION AND OLAPTESED PEGOL (NOX-A12) IN UNMETHYLATED PATIENTS
**Study Acronym:**	GLORIA
**EudraCT Number:**	2018-004064-62
**IND:**	NA
**Investigational Product:**	Olaptesed pegol (NOX-A12)
**Clinical Phase:**	1/2
**Indication:**	Glioblastoma
**Sponsor:**	NOXXON Pharma AG<br>Max-Dohrn-Str. 8-10<br>10589 Berlin, Germany<br>Tel: +49 30 726247100<br>Fax: +49 30 726247225<br>Email: clinic@noxxon.com
**Sponsor Representative:**	[redacted]
**Version:**	Version 7.0
**Date:**	14-Mar-2022
**Safety Reporting:**	[redacted]
**Emergency Call:**	[redacted]

Confidential: The information contained in this document, particularly unpublished data, is in the property or under control of NOXXON Pharma AG and is provided to you in confidence as an investigator, potential investigator, or consultant, for review by you, your staff, and an applicable Institutional Review Board or Independent Ethics Committee. The information is only to be used by you in connection with authorized clinical studies of the investigational drug described in the protocol.

Protocol Approval Signature Page

This clinical study protocol was subject to critical review and has been approved by the sponsor. The information it contains is consistent with:

- the current risk-benefit evaluation of the investigational product.
- the moral, ethical, and scientific principles governing clinical research as set out in the Declaration of Helsinki and the principles of GCP as described in ICH GCP.

The investigator will be supplied with details of any significant or new findings, including adverse events, relating to treatment with the investigational product.

[redacted] Date

[redacted] Date

[redacted] Date

[redacted] Date

[redacted] Date

Investigator Signature Page

Study Title:

Single-arm, dose-escalation Phase 1/2 Study of olaptesed pegol (NOX-A12) in combination with irradiation in inoperable or partially resected first-line glioblastoma patients with unmethylated MGMT promoter with a 3-arm expansion group including fully resected patients and combination with bevacizumab or pembrolizumab

The undersigned hereby declares his/her consent to performance of the clinical study in compliance with regulations as laid down in this clinical study protocol, in the Declaration of Helsinki and in the ICH-GCP Guideline and applicable national laws and regulations. Changes to this protocol require written agreement of both investigator and sponsor.

The Investigator has acquainted himself with the results of the pharmacological and toxicological studies of the investigational product and the results of other studies as described in the investigator's brochure or other appropriate information.

Site Number: ______________________

Principal Investigator: ______________________

Institution: ______________________

Address: ______________________

Investigator:

______________________	______________________	______________
Printed Name	Signature	Date

2. Synopsis and Flow Chart

Title of Study:

Single-arm dose-escalation Phase 1/2 study of olaptesed pegol (NOX-A12) in combination with irradiation in inoperable or partially resected first-line glioblastoma patients with unmethylated MGMT promoter with a 3-arm expansion group including fully resected patients and combination with bevacizumab or pembrolizumab

Rationale:

Glioblastoma (GB) is the most common and aggressive primary malignant brain tumor in adults (1). The disease is characterized by high growth rate and invasiveness, marked angiogenesis, ability to escape the immune system and resistance to treatment (2).

Treatment with surgical resection, chemotherapy, and external-beam radiotherapy (EBRT) does not prevent tumor recurrence with less than 10% of patients reaching 5-years survival (1). There are two ways how tumors can re-establish the vasculature that is lost after radiotherapy: angiogenesis, which is stimulated by VEGF with the creation of neovasculature by sprouting from existing blood vessels, and vasculogenesis, where the new blood vessels are created *de novo* by vascular precursors cells recruited from the bone marrow; the key driver for this latter process is CXCL12 (3). Studies in mice and rats showed that radiation-induced devascularization is efficiently restored by vasculogenesis after recruitment of bone marrow–derived cells (BMDCs), primarily CD11b+ myelomonocytes. Inhibition of the CXCL12-CXCR4/CXCR7 axis with receptor antagonists, antibodies, or olaptesed pegol after tumor irradiation was able to inhibit the recruitment of the BMDCs and the development of functional tumor vasculature, resulting in abrogation of tumor regrowth (4-6). In contrast, inhibition of VEGF with aflibercept or bevacizumab had only little effect on glioblastoma, because BMDCs bypass anti-VEGF therapy (7-9).

There is broad and robust consensus that CXCL12 and VEGF have complementary and synergistic activity in vasculo- and angiogenesis in physiological and pathological conditions including (myocardial) ischemia or stroke (10-16). Over the past decades, this synergistic activity has been consistently detected to have a major role in the (re)nourishment of various tumors before and in response to anti-tumor therapies (17-20).

In the setting of glioblastoma, the rationale for combining CXCL12 and VEGF inhibition is derived from the processes in the tumor microenvironment that are initiated by radiotherapy – the mainstay of glioblastoma treatment – which has been reported to cause blood vessel damage that may lead to additional tumor cell killing (21). Depletion of microvasculature following radiotherapy results in weakly perfused or non-perfused areas of tumor in which surviving cells respond with overexpression of hypoxia induced factor 1 alpha (HIF-1). HIF-1 then induces expression of a wide variety of genes such as VEGF, which promotes the process of angiogenesis, driving sprouting of new vessels from nearby vessels (22,23), and CXCL12, which promotes vasculogenesis by attracting two types of bone marrow-derived cell populations to build new blood vessels; endothelial progenitor cells via CXCR7 and pro-neovascularizing bone marrow-derived monocytes via CXCR4.

Kioi et al. (4) published data supporting the hypothesis that tumor irradiation sterilizes sufficient number of endothelial cells in and adjacent to the tumor to abrogate angiogenesis which forces the tumor to rely on the vasculogenesis pathway to rebuild blood vessels. However, others (24) suggested that the small fraction of endothelial cells surviving radiotherapy would be capable of responding to VEGF with angiogenesis-driven revascularization. Greenfield thus hypothesized that (VEGF-driven) angiogenesis and (CXCL12-driven) vasculogenesis are both active in glioblastoma following radiotherapy.

This implies that CXCL12 and VEGF might thus each play a part in treatment escape when the other pathway is inhibited. A complementary role for CXCL12 and VEGF in tumor revascularization is further supported by the more recently available information that CXCL12 and VEGF are expressed preferentially in different GBM tumor compartments (25,26).

Assessment of the relevance of CXCL12-driven vasculogenesis and VEGF-driven angiogenesis thus provide a rationale for the hypothesis that addition of VEGF inhibition has the potential to synergize with CXCL12 inhibition by olaptesed pegol: Both processes appear to contribute to the recurrence of glioblastoma tumors after radiotherapy, and they are complementarily active in different substructures of the tumor.

This escape mechanism might be the reason why in Phase 3 trials with the VEGF inhibitor bevacizumab in newly diagnosed glioblastoma patients no improved overall survival was observed (7,8); bevacizumab was therefore not approved for this indication. Of note, the reason for the EMA's refusal was the absence of established efficacy, as *"overall, the toxicity of bevacizumab in this regimen and combination did not, in itself, raise major concerns"* (EMA Annex: Scientific conclusions and grounds for refusal of the variation presented by the European Medicines Agency).

The fact that VEGF blockade is effectively bypassed by CXCL12 expression in glioblastoma (9) together with our own data showing that VEGF blockade synergized with olaptesed pegol in an animal model where this combination treatment reduced not only the number of pro-angiogenic macrophages and the microvessel density but also prolonged overall survival (27) warrant the testing of this combination in a clinical study.

Anti-PD1 immunotherapy as a single agent (Checkmate-143, NCT02017717) or in combination with radiotherapy (Checkmate-498, NCT02617589), or radiochemotherapy (Checkmate-548, NCT02667587) was assessed in Phase 3 clinical trials, and has shown only modest effect in patients with glioblastoma (28,29). The reason for that is believed to be due to the tumor immunologically "cold" milieu characterized by a scarcity of tumor infiltrating lymphocytes (TILs) and a predominance of immunosuppressive myeloid cells (30). Emerging evidence suggests that myeloid cells play a critical role in glioblastoma immunosuppression which makes combination immunotherapy with agents such as a CXCL12 inhibitor a promising option to modulate the microenvironment by partially depleting immunosuppressive myeloid cells and hopefully rendering the tumor more sensitive to checkpoint inhibitors treatment. There is some preclinical evidence supporting our hypothesis, a study in an orthotopic syngeneic mouse model explored the blockade of the CXCL12/CXCR4 axis by comparing the combination of anti PD-1 and the CXCR4 inhibitor AMD3100 with both monotherapy controls. Animals receiving the combination therapy had lower numbers of immunosuppressive tumor-infiltrating leukocytes, a significantly decreased CD4+/CD8+ lymphocyte ratio, and increased levels of circulating inflammatory antitumoral cytokines, all of which translated into a survival

benefit and long-term protective immunity compared to the monotherapy arms (31). Animals in this study which achieved long-term survival following anti-PD-1 and CXCL12/CXCR4 blockade were able to completely resist re-challenge with a second implantation of tumor cells, indicating durable immune response.

The clinical experience with olaptesed pegol ranges from studies in healthy subjects to studies in patients with relapsed/ refractory CLL in combination with rituximab and bendamustine, in patients with relapsed/ refractory multiple myeloma (MM) in combination with bortezomib and dexamethasone, and patients with metastatic colorectal and pancreatic cancer in combination with the PD-1 checkpoint inhibitor pembrolizumab. In the CLL and MM studies, treatment with olaptesed pegol resulted in high overall and complete response rates (CLL: 86% ORR; MM: 68% ORR) and was safe and well tolerated, i.e. without appreciable additional adverse events than previously reported for the respective background therapies; in the study with solid tumors the adverse event profile was comparable with the safety profile for pembrolizumab or typical for the underlying diseases (32-34).

In GBM, patients with resectable tumor and methylated MGMT promoter have a median survival of approximately 24 months when treated with irradiation and temozolomide. However, there is no efficient therapy for patients characterized by non-resectable tumors with unmethylated MGMT promoter, which are temozolomide resistant (35).

In this Phase 1/2 study, it is planned to treat patients with glioblastoma (36) and unmethylated MGMT promoter with external-beam radiotherapy (60 Gy in daily fractions of 2 Gy or, for elderly patients, 40.05 Gy in daily fractions of 2.67 Gy, 5 per week, Mo through Fr) in combination with the anti-CXCL12 Spiegelmer olaptesed pegol.

The safety database will be extended to evaluate future clinical development by including a 3-arm expansion group. With these three arms, additional data will be collected from (i) patients with MGMT unmethylated promoter and fully resected tumors who will receive olaptesed pegol in addition to radiotherapy and (ii) patients with MGMT unmethylated promoter and fully resected, unresected or only partially resected tumors who will receive olaptesed pegol in addition to radiotherapy and either bevacizumab or pembrolizumab. The latter two patient arms will be included to establish safety for these combinations and also to explore a potential benefit from combing olaptesed pegol and bevacizumab or pembrolizumab.

Phase of development:

1/2

Patient Population:

Dose escalation cohorts:

Patients with newly diagnosed glioblastoma (WHO grade IV) of unmethylated MGMT promoter status either not amenable to resection (biopsy only) or after incomplete tumor resection

Expansion group:

Arm A: Patients with newly diagnosed glioblastoma (WHO grade IV) with unmethylated MGMT promoter status after complete, incomplete or no tumor resection

Arm B: Patients with newly diagnosed glioblastoma (WHO grade IV) with unmethylated MGMT promoter status after complete resection

Arm C: Patients with newly diagnosed glioblastoma (WHO grade IV) with unmethylated MGMT promoter status after complete or incomplete tumor resection

Studied duration (planned):

Screening period: up to 4 weeks

Treatment period: up to 6 months

Follow up period: ends 24 months after first treatment of last patient

Number of patients (planned):

up to 18 in dose escalation cohorts, 18 patients in the expansion group (6 in each arm)

Type of study, study design:

Multi-center, prospective, open-label, single-arm dose escalation part and a 3-arm expansion group

Study objectives:

Primary objective:

- To investigate the safety of either olaptesed pegol in combination with radiation therapy or olaptesed pegol combination with radiation therapy and bevacizumab or pembrolizumab in patients with newly diagnosed glioblastoma of unmethylated MGMT promoter status

Secondary objectives:

- To explore the efficacy of olaptesed pegol in combination with radiation therapy on patients with glioblastoma
- To investigate the efficacy of olaptesed pegol in combination with radiation therapy and bevacizumab or pembrolizumab in patients with glioblastoma of unmethylated MGMT promoter status
- To investigate the pharmacokinetics of olaptesed pegol during continuous administration
- To monitor symptoms (NANO) and QoL

Study endpoints:

Primary endpoint:

- Safety (adverse events)

Secondary endpoints:

- PFS at 6 months (PFS-6)
- Median progression-free survival (mPFS)
- Median overall survival (mOS)

- Tumor vascularization as per vascular MRI scans at baseline and 2, 4, and 6 months
- Topography of recurrence
- Determination of maximum tolerated dose (MTD)
- Definition of recommended Phase 2 dose (RP2D)
- Olaptesed plasma levels at steady state
- NANO assessment
- Quality of Life

<u>Criteria for inclusion - Dose escalation cohorts:</u>

1. Written informed consent
2. Age ≥ 18 years
3. Patient agreement to diagnostic and scientific work-up of glioblastoma tissue obtained during the preceding surgery or biopsy (e.g., MGMT promoter analysis, cytogenetic markers such as IDH-1 mutations, etc.)
4. Patient agrees to subcutaneous port implantation
5. Newly diagnosed, histologically confirmed, supratentorial WHO grade IV glioblastoma
6. Status post biopsy or incomplete resection (detectable residual tumor as per postoperative T1-weighted, contrast-enhanced MRI scan)
7. Unmethylated MGMT promoter status
8. Maximum Eastern Cooperative Oncology Group (ECOG) score 2
9. Estimated minimum life expectancy 3 months
10. Stable or decreasing dose of corticosteroids during the week prior to inclusion
11. The following laboratory parameters should be within the ranges specified:
    - Total bilirubin ≤ 1.5 x upper limit normal (ULN)
    - Creatinine ≤ 1.5 x ULN or glomerular filtration rate ≥ 60 mL/min/1.73m²
    - ALT (alanine transaminase) ≤ 3 x ULN
    - AST (aspartate transaminase) ≤ 3 x ULN
12. Female patients of child-bearing potential must have a negative serum pregnancy test within 21 days prior to enrollment and agree to use a highly effective method of birth control (failure rate less than 1% per year when used consistently and correctly such as contraceptive implants, vaginal rings, sterilization, or sexual abstinence) during and for 3 months following last dose of drug (more frequent pregnancy tests may be conducted if required per local regulations)
13. Male patients must use an effective barrier method of contraception during study and for 3 months following the last dose if sexually active with a FCBP

<u>Criteria for inclusion - Expansion Group:</u>

1. Written informed consent
2. Age ≥ 18 years

3. Patient agreement to diagnostic and scientific work-up of glioblastoma tissue obtained during the preceding surgery or biopsy (e.g., MGMT promoter analysis, cytogenetic markers such as IDH-1 mutations, etc.)
4. Patient agrees to subcutaneous port implantation
5. Newly diagnosed, histologically confirmed, supratentorial WHO grade IV glioblastoma
6. a) Status post biopsy or incomplete (detectable residual tumor as per postoperative T1-weighted, contrast-enhanced MRI scan) or complete resection (Arm A)

   OR

   b) Status post complete resection (Arm B)

   OR

   c) Status post complete or incomplete resection (circumscribed enhancing tumor ≤ 5.0 cm in largest diameter as per postoperative T1-weighted, contrast-enhanced MRI scan) (Arm C)
7. Unmethylated MGMT promoter status
8. Maximum Eastern Cooperative Oncology Group (ECOG) score 2
9. Estimated minimum life expectancy 3 months
10. Stable or decreasing dose of corticosteroids during the week prior to inclusion
11. The following laboratory parameters should be within the ranges specified:
    - Total bilirubin ≤ 1.5 x upper limit normal (ULN)
    - Creatinine ≤ 1.5 x ULN or glomerular filtration rate ≥ 60 mL/min/1.73m²
    - ALT (alanine transaminase) ≤ 3 x ULN
    - AST (aspartate transaminase) ≤ 3 x ULN
12. Female patients of child-bearing potential must have a negative serum pregnancy test within 21 days prior to enrollment and agree to use a highly effective method of birth control (failure rate less than 1% per year when used consistently and correctly such as contraceptive implants, vaginal rings, sterilization, or sexual abstinence) during and for 3 months (6 months Arm A, 4 months Arm C) following last dose of drug (more frequent pregnancy tests may be conducted if required per local regulations)
13. Male patients must use an effective barrier method of contraception during study and for 3 months (6 months Arm A, 4 months Arm C) following the last dose if sexually active with a FCBP

Criteria for exclusion - Dose escalation cohorts:

1. Inability to understand and collaborate throughout the study or inability or unwillingness to comply with study requirements
2. Participation in any clinical research study with administration of an investigational drug or therapy within 30 days from screening visit or observation period of competing studies
3. Contra-indication or known hypersensitivity to MRI contrast agents, olaptesed pegol or polyethylene glycol
4. Cytostatic therapy (chemotherapy) within the past 5 years

5. History of other cancers (except for adequately treated basal or squamous cell skin cancer, in situ cervical cancer, or other cancer from which the patient was disease-free for ≥ 5 years)
6. Clinically significant or uncontrolled cardiovascular disease, including
   - Myocardial infarction in the previous 12 months
   - Uncontrolled angina
   - Congestive heart failure (New York Heart Association functional classification of ≥2)
   - Diagnosed or suspected congenital long QT syndrome
   - QTc prolongation on an electrocardiogram prior to entry (>470 ms)
   - Uncontrolled hypertension (blood pressure ≥ 160/95 mmHg)
   - Heart rate <50/min on the baseline electrocardiogram
   - History of ventricular arrhythmias of any clinically significant type (such as ventricular tachycardia, ventricular fibrillation or torsades de pointes)
7. Prior radiotherapy to the head
8. Any other previous or concomitant experimental glioblastoma treatments
9. Placement of Gliadel® wafer, seeds, or ferromagnetic nanoparticles
10. Pregnancy or lactation
11. Uncontrolled intercurrent illness including, but not limited to ongoing or active infection, chronic liver disease (e.g., cirrhosis, hepatitis), diabetes mellitus, or subjects with either of the following: fasting blood glucose (FBG defined as fasting for at least 8 hours) ≥ 200 mg/dL (7.0 mmol/L), or HbA1c ≥ 8%, chronic renal disease, pancreatitis, chronic pulmonary disease, or psychiatric illness/social situations that would limit compliance with study requirements. Patients must be free of any clinically relevant disease (other than glioma) that would, in the treating investigator's opinion, interfere with the conduct of the study or study evaluations.
12. Treatment not initiated within 6 weeks after first biopsy or surgery of glioblastoma
13. Prior enrolment into this study

Criteria for exclusion - Expansion group, Arms A and B:

1. Inability to understand and collaborate throughout the study or inability or unwillingness to comply with study requirements
2. Participation in any clinical research study with administration of an investigational drug or therapy within 30 days from screening visit or observation period of competing studies
3. Contra-indication or known hypersensitivity to MRI contrast agents, bevacizumab (Arm A only), olaptesed pegol or polyethylene glycol
4. Planned hypofractionated radiotherapy
5. Cytostatic therapy (chemotherapy) within the past 5 years
6. History of other cancers (except for adequately treated basal or squamous cell skin cancer, in situ cervical cancer, or other cancer from which the patient was disease-free for ≥ 5 years)
7. Secondary malignancy which is currently active

8. Clinically significant or uncontrolled cardiovascular disease, including
   - Myocardial infarction in the previous 12 months
   - Uncontrolled angina
   - Congestive heart failure (New York Heart Association functional classification of ≥2)
   - Diagnosed or suspected congenital long QT syndrome
   - QTc prolongation on an electrocardiogram prior to entry (>470 ms)
   - Uncontrolled hypertension (blood pressure ≥ 160/95 mmHg)
   - Heart rate <50/min on the baseline electrocardiogram
   - History of ventricular arrhythmias of any clinically significant type (such as ventricular tachycardia, ventricular fibrillation or torsades de pointes)
   - Cerebrovascular accident
9. Prior radiotherapy to the head
10. Any other previous or concomitant experimental glioblastoma treatments
11. Placement of Gliadel® wafer, seeds, or ferromagnetic nanoparticles
12. Patients with a history of arterial or venous thrombosis (or any other disease) requiring permanent intake of anticoagulants (Arm A only)
13. Pregnancy or lactation
14. Uncontrolled intercurrent illness including, but not limited to ongoing or active infection, chronic liver disease (e.g., cirrhosis, hepatitis), diabetes mellitus, or subjects with either of the following: fasting blood glucose (FBG defined as fasting for at least 8 hours) ≥ 200 mg/dL (7.0 mmol/L), or HbA1c ≥ 8%, chronic renal disease, pancreatitis, chronic pulmonary disease, auto-immune diseases or psychiatric illness/social situations that would limit compliance with study requirements. Patients must be free of any clinically relevant disease (other than glioma) that would, in the treating investigator's opinion, interfere with the conduct of the study or study evaluations.
15. Prolongation of coagulation factors ≥ 2.5 x ULN (Arm A only)
16. Treatment not initiated within 6 weeks after first biopsy or surgery of glioblastoma
17. Prior enrolment into this study

Criteria for exclusion – Expansion group, Arm C:

1. Inability to understand and collaborate throughout the study or inability or unwillingness to comply with study requirements
2. Participation in any clinical research study with administration of an investigational drug or therapy within 30 days from screening visit or observation period of competing studies
3. Contra-indication or known hypersensitivity to MRI contrast agents olaptesed pegol or polyethylene glycol or pembrolizumab (≥ Grade 3)
4. Biopsy-only of GBM with less than 20% of tumor removed
5. Presence of extracranial metastatic or leptomeningeal disease
6. Severe hypersensitivity (≥ Grade 3) to other monoclonal antibodies
7. Receiving immunosuppressive therapy

8. Previous or current treatment with an anti-CTLA-4, anti-PD-1, anti-PD-L1, or anti-PDL2 agent
9. Planned hypofractionated radiotherapy
10. Cytostatic therapy (chemotherapy) within the past 5 years
11. History of other cancers or secondary malignancy which is currently active (except for adequately treated basal or squamous cell skin cancer, in situ cervical cancer, or other cancer from which the patient was disease-free for ≥ 5 years)
12. Clinically significant or uncontrolled cardiovascular disease, including
    - Myocardial infarction in the previous 12 months
    - Uncontrolled angina
    - Congestive heart failure (New York Heart Association functional classification of ≥2)
    - Diagnosed or suspected congenital long QT syndrome
    - QTc prolongation on an electrocardiogram prior to entry (>470 ms)
    - Uncontrolled hypertension (blood pressure ≥ 160/95 mmHg)
    - Heart rate <50/min on the baseline electrocardiogram
    - History of ventricular arrhythmias of any clinically significant type (such as ventricular tachycardia, ventricular fibrillation or torsades de pointes)
    - Cerebrovascular accident
13. Prior radiotherapy to the head
14. Evidence of acute intracranial / intra-tumoral hemorrhage
15. Any other previous or concomitant experimental glioblastoma treatments
16. Placement of Gliadel® wafer, seeds, or ferromagnetic nanoparticles
17. Pregnancy or lactation
18. Uncontrolled intercurrent illness including, but not limited to ongoing or active infection, chronic liver disease (e.g., cirrhosis, hepatitis), diabetes mellitus, or subjects with either of the following: fasting blood glucose (FBG defined as fasting for at least 8 hours) ≥ 200 mg/dL (7.0 mmol/L), or HbA1c ≥ 8%, chronic renal disease, pancreatitis, chronic pulmonary disease, auto-immune diseases or psychiatric illness/social situations that would limit compliance with study requirements. Patients must be free of any clinically relevant disease (other than glioma) that would, in the treating investigator's opinion, interfere with the conduct of the study or study evaluations.
19. Received a live vaccine within 30 days prior to the first dose of study drug.
20. Known active central nervous system (CNS) metastases and/or carcinomatous meningitis. Previously treated brain metastases may participate provided these remain stable
21. Known history of HIV infection, hepatitis B or hepatitis C infection
22. Active autoimmune disease that has required systemic treatment in past 2 years (i.e. with use of disease modifying agents, corticosteroids or immunosuppressive drugs)
23. History of (non-infectious) pneumonitis / interstitial lung disease that required steroids or current pneumonitis / interstitial lung disease

24. Immunodeficiency diagnosis or receiving chronic systemic steroid therapy (exceeding 10 mg daily of prednisone) or any other form of immunosuppressive therapy
25. High dose of corticosteroids (> 4mg/day of dexamethasone or equivalent for at least 3 consecutive days) within two weeks prior to the first dose of study drug
26. Treatment not initiated within 6 weeks after first biopsy or surgery of glioblastoma
27. Prior enrolment into this study

Investigational medicinal product:

Olaptesed pegol; presented in a single-use vial containing a solution with 100 mg/6.8 mL of olaptesed pegol

MVASI® (Bevacizumab): presented in a single-use vial containing a solution with 100 mg/4 mL or 400 mg/16 mL

KEYTRUDA® (pembrolizumab): presented in a single-use vial containing a solution with 100mg/4mL

Treatments, dose, mode of administration, duration of treatment:

During weeks 1 to 6, radiotherapy will be administered as EBRT to a cumulative dose of 60 Gy in daily fractions of 2 Gy (5 per week, Mo through Fr). Patients enrolled in the dose escalation cohorts aged 65 years or older may also undergo hypofractionated radiotherapy (40.05 Gy in 15 fractions). Hypofractionated radiotherapy is not allowed for patients in the expansion group.

Olaptesed pegol will be administered i.v. by continuous infusion using an active pump. For the first 24 hours olaptesed pegol will be administered at rates of 70, 160, and 230 mg/day and thereafter, at a rate of 200, 400 or 600 mg/week throughout the 6-week period of radiotherapy and the following 20 weeks. Radiotherapy starts on treatment week 1 day 2.

MVASI will be administered by i.v. infusion at doses of 10mg/kg every 2 weeks for 26 weeks (7,8).

KEYTRUDA will be administered by i.v. infusion at doses of 200 mg every 3 weeks for 26 weeks.

Cohort	Treatments	Weeks 1-6	Weeks 7-26
1	Olaptesed pegol	200 mg/week continuous infusion	200 mg/week continuous infusion
	Irradiation	2 Gy, (2.67 Gy for elderly), Mo-Fr	-
2	Olaptesed pegol	400 mg/week continuous infusion	400 mg/week continuous infusion
	Irradiation	2 Gy, (2.67 Gy for elderly), Mo-Fr	-
3	Olaptesed pegol	600 mg/week continuous infusion	600 mg/week continuous infusion
	Irradiation	2 Gy, (2.67 Gy for elderly), Mo-Fr	-
Arm A	Olaptesed pegol	600 mg/week continuous infusion	600 mg/week continuous infusion
	Irradiation	2 Gy Mo-Fr	-
	MVASI	10 mg/kg every 2 weeks	10 mg/kg every 2 weeks

Cohort	Treatments	Weeks 1-6	Weeks 7-26
Arm B	Olaptesed pegol	600 mg/week continuous infusion	600 mg/week continuous infusion
	Irradiation	2 Gy Mo-Fr	-
Arm C	Olaptesed pegol	600 mg/week continuous infusion	600 mg/week continuous infusion
	Irradiation	2 Gy Mo-Fr	
	KEYTRUDA	200 mg every 3 weeks	200 mg every 3 weeks

Interruption of olaptesed pegol treatment is not permitted for more than 2 consecutive days and/or 7 days in total. Longer interruption of treatment with olaptesed pegol will be considered as major protocol violation.

Dose escalation part: After 4 weeks of olaptesed pegol treatment of the last patient in each cohort, the DSMB will review all DLTs, all AEs, and relevant laboratory values before initiation of the next cohort.

Expansion group, Arm A: After 4 weeks of treatment of the first patient with olaptesed pegol in combination with bevacizumab, the DSMB will review all DLTs, all AEs, and relevant laboratory values.

Expansion group, Arm C: After 4 weeks of treatment of the first patient with olaptesed pegol in combination with pembrolizumab, the DSMB will review all DLTs, all AEs, and relevant laboratory values.

The DSMB will also meet on an ad hoc basis if 2 or more DLTs are observed in any cohort. Furthermore, the DSMB may recommend decreasing the weekly-infused dose based on the results from safety evaluations.

Criteria for evaluation:

Safety:

The following safety parameters will be evaluated during screening, throughout the treatment and follow up periods as outlined below at the time points provided in the study flow-chart:

- Adverse events
- Dose limiting toxicities
- Vital signs (pulse rate, blood pressure)
- Hematology (full blood count including platelets and differential count)
- Safety laboratory
- Concomitant medication, incl. steroids
- Immunogenicity of olaptesed pegol

Unscheduled safety tests may be performed at any time if clinically indicated.

Efficacy:

- Response assessed according to most recent RANO criteria (37) at 2, 4, 6 months after treatment start and subsequently at 3-monthly intervals

- Time-to event and survival analysis at 6, 9, 12, 15, 18, 21, 24 months
- Tumor vascularization as per vascular MRI scans at baseline and 2, 4, and 6 months of treatment with olaptesed pegol and following at 3-monthly intervals
- NANO assessment (38), neurological assessments
- Quality of Life assessed by European Organization for Research and Treatment (EORTC) Quality of Life Questionnaires (QLQ C30/BN20)
- Determination of MTD
- Determination of RP2D

Pharmacodynamics:

- Mobilization of white blood cells
- CXCL12 concentration in plasma
- Exploratory biomarker analysis (optional)

Pharmacokinetics:

- Plasma levels of olaptesed pegol prior to and during treatment

Data Safety Monitoring Board:

A data safety monitoring board is set up to review the safety information arising from this clinical study and to give recommendations as to continuation of treatment at certain doses, dose reductions, or as to the continuation of the clinical study in its entirety.

Statistical methods:

No formal sample size calculations were performed for this standard dose-escalation 3+3 design. In addition, a further 12 patients will be enrolled (Arm A: 6 patients; Arm B: 6 patients) to obtain first results for a combined therapy with bevacizumab (Arm A) and fully resected (Arm B). Analyses for all endpoints will be performed descriptively. Another 6 patients will be enrolled (Arm C) to obtain first results for a combined therapy with pembrolizumab. No hypotheses will be tested.

Continuous endpoints will be analyzed by descriptive statistics in terms of means, standard deviation, median, the first, and third quartile, minimum and maximum. Categorical endpoints will be analyzed by absolute and relative frequencies.

The primary endpoint of this study is the safety in terms of adverse events with focus on DLTs. Efficacy will be assessed as exploratory analysis.

This dose-escalation study is designed to enroll successive cohorts of patients (at least 3 patients/cohort) with three dose levels, 200, 400, and 600 mg/week. Dose escalation is to be halted when the maximum tolerated dose (MTD) was reached. The MTD is defined as the dose level below that at which the DLT was observed in one-third or more patients. If one of the patients in a dose cohort experienced a DLT, three more patients are to be added to the cohort. If no further DLT is observed in the group, an additional cohort of three patients is to be enrolled at the next higher dose (39).

2.1 Schedule of Assessments and Flow Chart

Table 2-1: Schedule of assessments

	Screen		Treatment Phase				Follow Up
	≤28 days		Irradiation + Olaptesed		Olaptesed		Until W104
**Study Day (D) / Week (W) / Month (M)**	**D-28 to -8**	**D-7 to -1**	**Week 1-6**		**Week 7 - 26**	**EOT**	**Q3M**
			**D1**	**D2-D5**	**W9,18,26**		
Informed consent	X						
Inclusion / exclusion criteria, eligibility	X						
Pregnancy test	X		W1, 5, 9,13,18,21			X	
Medical history incl. demography	X						
Physical examination	X					X	X
Neurological examination	X			end of RT	W9,18	X	X
12-lead ECG	X		W1, 5, 9, 13, 18, 21			X	
Vital signs	x		weekly				X
QoL questionnaire	X				W9	X	X
Blood sampling							
Clinical chemistry	X		X		W9,13,15,18,21,24	X	X
Hematology	X		X		W9,18	X	X
Coagulation	X						
PK			W1[a],2,4,6	W1 (D2+5)	W9		
Immunogenicity	X		W1[a],2,4,6	W1 (D2+5)	W9		M9,12
Biomarkers (e.g. CXCL12)	X		W1[a],2,4,6	W1D2	W9,18	X	X
Pharmacogenetic			X[a]				
Response Assessment:							
Imaging (multiparametric MRI)		X			W9,18	X[f]	X
RANO criteria		X			W9,18	X[f]	X
Port Implant/Explant		IMPL				EXPL[b]	
Radiotherapy (RT)			X[c]	X[d]			
Olaptesed pegol[e]			X→	→	→X		
Bevacizumab (Arm A)			W1, 3, 5, 7, 9, 11, 13, 15, 17, 19, 21, 23, 25				
Pembrolizumab (Arm C)			W1, 4, 7, 10, 13, 16, 19. 22, 25				
Adverse Events	X→	→	→	→	→	→X	
Concomitant medication	X→	→	→	→	→	→	→X
Survival							X

a: pre-dose; b: time point may vary after Olaptesed Pegol administration (as per patient/investigator decision); c: RT starts on Day 2 of Week 1; d: RT may continue until week 3 for elderly patients in the dose escalation cohort and until week 6 for all other patients; e: continuous infusion with weekly changes of container; f: only at regular end of treatment after 26 weeks

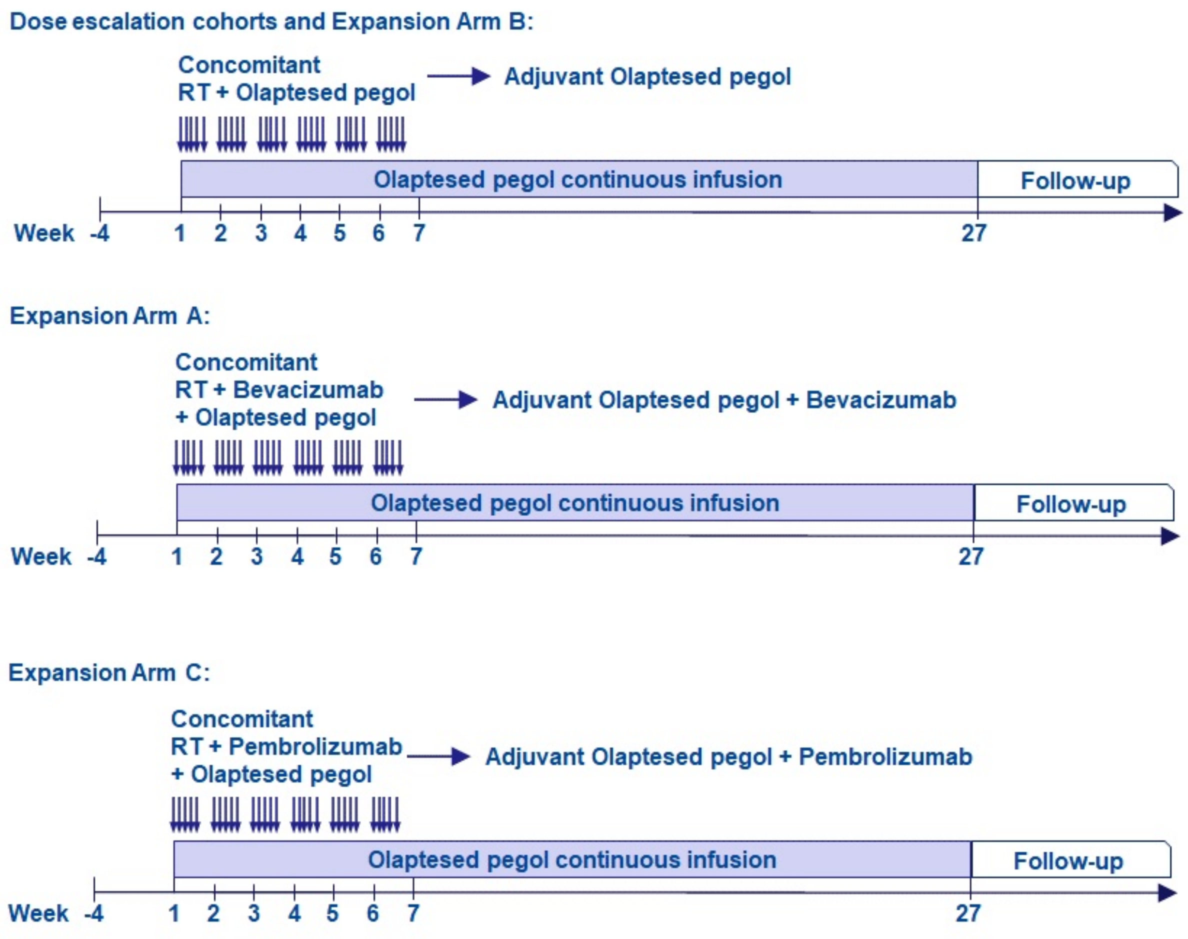

Figure 2-1: Study outline

3. Table of Contents

Protocol Approval Signature Page 2
Investigator Signature Page 3
2. Synopsis and Flow Chart 4
2.1 Schedule of Assessments and Flow Chart 16
3. Table of Contents 18
3.1 List of In-Text Tables 23
3.2 List of In-Text Figures 23
4. List of Abbreviations and Definition of Terms 24
5. Study Administrative Structure 26
6. Introduction 27
6.1 Background 27
6.2 Investigational Medicinal Product 28
6.2.1 Olaptesed Pegol 28
6.2.2 Bevacizumab 28
6.2.3 Pembrolizumab 28
6.3 Non-Clinical Data 28
6.4 Clinical Data 32
6.5 Study Design Rationale 35
6.6 Benefit / Risk Assessment 36
7. Study Objectives and Endpoints 39
7.1 Primary Objective 39
7.2 Primary Endpoint 39
7.3 Secondary Objectives 39
7.4 Secondary Endpoints 39
8. Investigational Plan 40
8.1 Overall Study Design 40
8.2 Choice of Control Groups 40
8.3 Selection of Doses 40
8.3.1 Olaptesed pegol 40
8.3.2 Bevacizumab 46
8.3.3 Pembrolizumab 46
8.4 Selection of Study Population 47
8.4.1 Inclusion Criteria 47

8.4.2 Exclusion Criteria 49
8.4.3 Patient Withdrawal 52
8.4.4 Replacement of Patients 53
8.4.5 Restrictions during the Study 53
8.4.6 Termination of the Study 55
8.5 Treatments 55
8.5.1 Identity of Investigational Product(s) 55
8.5.2 Packaging and Labelling 56
8.5.3 Preparation 58
8.5.4 Complaints 58
8.5.5 Olaptesed Pegol Treatment Doses and Administration 59
8.5.6 Radiotherapy 61
8.5.7 Dose Limiting Toxicities 63
8.5.8 Dose Modification 63
8.5.9 Prior and Concomitant Therapy 67
8.5.10 Rescue Medication 67
8.5.11 Treatment after End of the Study 67
8.5.12 Drug Accountability and Treatment Compliance 68
8.5.13 Method of Assigning Patients to Treatment Groups 68
8.5.14 Blinding 68
9. Study Procedures and Assessments 69
9.1 Study Visits 69
9.1.1 Informed Consent Visit / Screening 69
9.1.2 Treatment Period: Week 1 70
9.1.3 Treatment Period: Week 2 - 6 70
9.1.4 Treatment Period: Week 7, 8, 10, 11, 12, 13, 14, 15, 16, 17, 19, 20, 21, 22, 23, 24, 25, 26 71
9.1.5 Treatment Period: Week 9, 18 and End of Treatment 71
9.1.6 Follow-up 72
9.1.7 Premature Study Termination 73
9.1.8 Time Windows 73
9.2 Efficacy Assessments 74
9.2.1 Response Assessments 74
9.2.2 Tumor Vascularization 74
9.2.3 Neurological Examination 74
9.2.4 Time to Event Time Points 74

9.2.5 Quality of Life Assessments 74
9.2.6 Pharmacokinetics 74
9.2.7 Pharmacodynamics 75
9.2.8 Pharmacogenetics 75
9.3 Demographics and Medical History 76
9.4 Safety Assessments 76
9.4.1 Physical Examination 76
9.4.2 Vital Signs 76
9.4.3 ECG 76
9.4.4 Clinical Laboratory Evaluation 77
9.4.5 Pregnancy Test 77
9.4.6 Immunogenicity 77
10. Pharmacovigilance 78
10.1 Definition of Adverse Events 78
10.2 Definition of Serious Adverse Events 78
10.3 Period of Observation 79
10.4 Recommendations to treat Overdosing and Intoxication with the Study Medication 80
10.5 Documentation and Reporting of Adverse Events 80
10.5.1 Nature 80
10.5.2 Severity 81
10.5.3 Duration 81
10.5.4 Relationship 81
10.5.5 Outcome 82
10.6 Documentation and Reporting of Serious Adverse Events 83
10.7 Reporting of Pregnancy and Lactation to the Sponsor 84
10.8 Follow-up of Missing Information 84
10.9 Deaths 85
10.10 Expedited Reporting of Adverse Events 85
10.10.1 SUSAR Reporting 85
10.10.2 Development Safety Update Reports 85
11. Statistical Methods 86
11.1 Determination of Sample Size 86
11.2 Statistical and Analytical Plans 86
11.3 Summary of Variables 86
11.4 Data Sets to be Analyzed 86

11.4.1 Safety Analysis Set 86
11.4.2 Full Analysis Set 87
11.5 Analysis of Study Conduct and Patient Disposition 87
11.6 Analysis of Baseline and Background Characteristics 87
11.7 Efficacy Analyses 87
11.8 Safety Analyses 87
11.8.1 Adverse Events 87
11.8.2 Clinical Laboratory 88
11.8.3 Physical Examination and Other Safety Measures 88
11.9 Pharmacokinetics 88
11.10 Pharmacodynamics 88
11.11 Subgroup Analyses 88
11.12 Interim Analyses 88
11.13 Handling of Dropouts and Missing Data 88
12. Data Collection, Handling, and Record Keeping 89
12.1 Generation of Data Base 89
12.2 Data Collection 89
12.2.1 Source Data 89
12.3 Data Management 90
12.4 Data Protection 90
13. Quality Assurance 92
13.1 Data Monitoring 92
13.2 Audits and Inspections 92
14. Ethical and Legal Requirements 93
14.1 Independent Ethics Committee 93
14.2 Ethical Conduct of the Study 93
14.3 Changes in the Conduct of the Study 93
14.4 Patient Information and Consent 93
14.5 Confidentiality 94
14.6 Finance, Patient Insurance Coverage and Investigator Indemnity 94
14.7 Publication of Study Results 95
15. Amendments to the Protocol 96
15.1 Protocol Amendment 1 96
15.1.1 Rationale for Protocol Amendment 96
15.1.2 Changes to the Protocol 96

15.2 Protocol Amendment 2 ... 104
15.2.1 Rationale for Protocol Amendment ... 104
15.2.2 Changes to the Protocol ... 104
15.3 Protocol Amendment 3 ... 113
15.3.1 Rationale for Protocol Amendment ... 113
15.3.2 Changes to the Protocol ... 113
15.4 Protocol Amendment 4 ... 131
15.4.1 Rationale for Protocol Amendment ... 131
15.4.2 Changes to the Protocol ... 131
15.5 Protocol Amendment 5 ... 146
15.5.1 Rationale for Protocol Amendment ... 146
15.5.2 Changes to the Protocol ... 146
15.6 Protocol Amendment 6 ... 158
15.6.1 Rationale for Protocol Amendment ... 158
15.6.2 Changes to the Protocol ... 158
15.7 Protocol Amendment 7 ... 159
15.7.1 Rationale for Protocol Amendment ... 159
15.7.2 Changes to the Protocol ... 159
15.8 Protocol Amendment 8 ... 160
16. Reference List ... 161

3.1 List of In-Text Tables

Table 2-1: Schedule of assessments 16
Table 6-1: Kaplan-Meier overall survival over 5 years (adapted from ref. (35)) 27
Table 8-1: Expected systemic exposure of GB patients continuously treated with iv olaptesed pegol at an infusion rate of 200, 400 and 600 mg/week 43
Table 8-2: Toxicities related to treatment with olaptesed pegol by cohort....45
Table 8-3: Plasma concentration Olaptesed pegol Cohort 3 45
Table 8-4: Olaptesed pegol Dose Modification Guidelines for drug-related Adverse Events 64
Table 8-5: MVASI Dosage Modifications for Adverse Reactions 64
Table 8-6: Recommended treatment modifications for KEYTRUDA....65
Table 10-1: WHO-UMC assessment criteria 82
Table 15-1: Summary of Changes Table....160

3.2 List of In-Text Figures

Figure 2-1: Study outline 17
Figure 8-1: Simulation of olaptesed plasma concentration for continuous infusion of 200, 400 and 600 mg/week olaptesed pegol....42
Figure 8-2: Dose escalation in three cohorts 60

4. List of Abbreviations and Definition of Terms

AE(s)	Adverse event(s)
ALT	Alanine aminotransferase
aPTT	activated partial thromboplastin time
AST	Aspartate aminotransferase
ATC	Anatomical Therapeutic Chemical
AUC	Area under the curve
BL	Baseline
BMDC	Bone marrow–derived cells
BMI	Body Mass Index
BP	Blood pressure
BUN	Blood urea nitrogen
CI	Confidence Interval
CLL	Chronic lymphocytic leukemia
C_{max}	Maximum observed plasma concentration
CNS	Central nervous system
CR	Complete response
CRO	Contract Research Organization
CT	Computer tomography
CTCAE	Common Terminology Criteria for Adverse Events
ECG	Electrocardiogram
ECOG	Eastern Co-operative Oncology Group
eCRF	Electronic Case Report Form
EMA	European Medicines Agency
EORTC	European Organization for Research and Treatment of Cancer
EoS	End of Study
EoT	End of Treatment
EU	European Union
FAS	Full Analysis Set
FCBP	Female of childbearing potential
FU	Follow up
GB	Glioblastoma
GCP	Good Clinical Practice
GGT	Gamma-Glutamyl transferase
i.v.	Intravenous
ICH	International Conference on Harmonisation of Technical Requirements for Pharmaceuticals for Human Use
IEC	Independent Ethics Committee
IMP	Investigational Medicinal Product
IRB	Institutional Review Board
IUD	Intrauterine device
IUS	Intrauterine hormone-releasing system

LDH	Lactate dehydrogenase
LOCF	Last Observation Carried Forward
MedDRA	Medical Dictionary for Regulatory Activities
MGMT	O6-Methylguanin-DNS-Methyltransferase
MM	Multiple Myeloma
MRI	Magnetic resonance imaging
N	Number
NANO	Neurologic Assessment in Neuro-Oncology
NOXXON	NOXXON Pharma AG
OS	Overall survival
PD	Pharmacodynamics, Progressive disease
PEG	Polyethylenglycol
PFS	Progression free survival
PK	Pharmacokinetics
PPS	Per Protocol Set
PR	Partial response
PT	Prothrombin time
PVI	Protracted intravenous infusion
q2d	Every second day
QoL	Quality of life
RANO	Response assessment in neuro-oncology criteria
RBC	Red blood cells
RNA	Ribonucleic Acid
RSI	Reference Safety Information
s.c.	Subcutaneous
SAE(s)	Serious Adverse Event(s)
SAF	Safety Analysis Set
SAP	Statistical Analysis Plan
SD	Standard Deviation, Stable Disease
SmPC	Summary of Product Charateristics
SOC	System Organ Class
SOP	Standard Operating Procedure
SUSAR	Suspected Unexpected Serious Adverse Reaction
TEAE	Treatment emergent adverse event
TMZ	Temozolomide
U/L	Units per liter
V_{24h}	24 h urine volume
vs.	Versus
WBC	White blood cells
WHO	World Health Organization
WHODD	World Health Organization Drug Dictionary
WMA	World Medical Association

5. Study Administrative Structure

The study will be conducted at 6 sites located in Germany.

A Contract Research Organization (CRO) will be responsible for the administration, coordination and monitoring of this study, as well as for enrollment of patients, data management and biometric analyses.

Laboratory analyses (blood and biomarker analyses) will be done by local laboratory facilities.

For the eCRF a specialized provider will be utilized.

A pharmacovigilance CRO will be responsible for pharmacovigilance services and safety monitoring.

A data safety monitoring board (DSMB) will be set up to review the safety information arising from this clinical study and to give recommendations as to continuation of treatment at certain doses, dose reductions, or as to the continuation of the clinical study in its entirety.

Further details / addresses and telephone numbers of main responsible persons will be provided in the pharmacovigilance, project management and quality plans.

6. Introduction

6.1 Background

Glioblastoma (GB) is the most common and aggressive primary malignant brain tumor in adults (1). The disease is characterized by high growth rate and invasiveness, marked angiogenesis, ability to escape the immune system and resistance to treatment (2).

Treatment with surgical resection, chemotherapy, and external-beam radiotherapy (EBRT) does not prevent tumor recurrence with less than 10% of patients reaching 5-years survival (1). There are two ways how tumors can re-establish the vasculature that is lost after radiotherapy: angiogenesis, which is stimulated by VEGF with the creation of neovasculature by sprouting from existing blood vessels, and vasculogenesis, where the new blood vessels are created *de novo* by vascular precursors cells recruited from the bone marrow; the key driver for this latter process is CXCL12 (3). Studies in mice and rats showed that radiation-induced devascularization is efficiently restored by vasculogenesis after recruitment of bone marrow–derived cells (BMDCs), primarily CD11b+ myelomonocytes. Inhibition of the CXCL12-CXCR4/CXCR7 axis with receptor antagonists, antibodies, or olaptesed pegol after tumor irradiation was able to inhibit the recruitment of the BMDCs and the development of functional tumor vasculature, resulting in abrogation of tumor regrowth (4-6). In contrast, inhibition of VEGF with aflibercept or bevacizumab had only little effect on glioblastoma, because BMDCs bypass anti-VEGF therapy (7-9).

In GBM, patients with resectable tumor and methylated MGMT promoter have a median survival of approximately 24 months when treated with irradiation and temozolomide, there is no efficient therapy for patients characterized by non-resectable tumors with unmethylated MGMT promoter, which exhibit a temozolomide resistant phenotype (Table 6-1,(35)).

Table 6-1: Kaplan-Meier overall survival over 5 years (adapted from ref. (35))

		Deaths / patients	Hazard ratio (95% CI)	Median (months; 95% CI)
**Complete resection**	Radiation only	109/113	1	14.2 (12.1-13.0)
	Radiation + Temozolomide	96/113	0.6 (0.5-0.7)	18.8 (16.4-22.9)
**Partial resection**	Radiation only	126/128	1	11.7 (9.7-13.1)
	Radiation + Temozolomide	113/126	0.6 (0.5-0.8)	13.5 (11.9-16.4)
**Biopsy only**	Radiation only	43/45	1	7.8 (6.4-10.6)
	Radiation + Temozolomide	45/48	0.7 (0.5-1.1)	9·4 (7·5-13·6)
**MGMT unmethylated**	Radiation only	54/54	1	11.8 (10.0-14.4)
	Radiation + Temozolomide	54/60	0.6 (0.4-0.8)	12.6 (11.6-14.4)

6.2 Investigational Medicinal Product

6.2.1 Olaptesed Pegol

The Spiegelmer olaptesed pegol is an inhibitor of the human chemokine CXCL12, also referred to as SDF-1. Olaptesed pegol is a Spiegelmer (i.e. an oligonucleotide aptamer possessing the unnatural L-stereoconfiguration) composed of 45 L-nucleotides which terminates at the 5'-end in a hexylamino linker, to which a branched 40 kDa monomethoxy-polyethylene-glycol (PEG) unit is covalently attached via an N-alkyl amide linkage. Olaptesed pegol has a molecular weight of approximately 54.5 kDa. The PEG moiety determines the pharmacokinetic behavior of the Spiegelmer and has been selected in order to increase the half-life in plasma. The oligonucleotide moiety, which is the part of the molecule that confers activity, has a molecular weight of 14,657 kDa. This molecular weight is used for the calculation of concentrations and doses.

6.2.2 Bevacizumab

Bevacizumab is a recombinant humanized monoclonal antibody produced by DNA technology in Chinese Hamster Ovary cells. Bevacizumab binds to vascular endothelial growth factor (VEGF), the key driver of vasculogenesis and angiogenesis, and thereby inhibits the binding of VEGF to its receptors, Flt-1 (VEGFR-1) and KDR (VEGFR-2), on the surface of endothelial cells. Neutralizing the biological activity of VEGF regresses the vascularization of tumors, normalizes remaining tumor vasculature, and inhibits the formation of new tumor vasculature, thereby inhibiting tumor growth.

6.2.3 Pembrolizumab

Pembrolizumab is a humanized monoclonal antibody which binds to the programmed cell death-1 (PD-1) receptor and blocks its interaction with ligands PD-L1 and PD-L2. The PD-1 receptor is a negative regulator of T-cell activity that has been shown to be involved in the control of T-cell immune responses. Pembrolizumab potentiates T-cell responses, including anti-tumor responses, through blockade of PD-1 binding to PD-L1 and PD-L2, which are expressed in antigen presenting cells and may be expressed by tumors or other cells in the tumor microenvironment.

6.3 Non-Clinical Data

Animal model to combine radiotherapy with CXCL12 inhibition: In preclinical studies using an autochthonous rat brain tumor model that mimics human glioblastoma, which is highly refractory to treatment, olaptesed pegol in combination with 20 Gy single dose irradiation (with or without TMZ) significantly reduced tumor burden and prolonged rat survival even if the rats were irradiated when the tumors were quite advanced as indicated by MRI monitoring. These preclinical data are strongly supported by clinical studies, e.g. clinical correlative studies have shown that elevated expression of SDF-1 and its receptors is associated with higher tumor grade and invasion and decreased apoptosis in glioblastoma (40,41). This novel approach provides a unique opportunity for glioblastoma patients, especially those with unmethylated MGMT promoter, as targeting rather the microenvironment and not the genetically instable

tumor cells renders olaptesed pegol therapy independent from any resistance mechanisms that might occur in cancer cells, i.e. TMZ resistance due to MGMT promoter methylation. Therefore, olaptesed pegol in combination with effective irradiation could be a promising approach for the treatment of glioblastoma for which there is a high unmet medical need.

Animal models to combine inhibition of VEGF and CXCL12 in glioblastoma: The combination of olaptesed pegol with VEGF blockade was tested in two orthotopic models, one in rats (syngeneic C6 glioblastoma) and another in mice (human G12 glioblastoma); the results were published in (27). In these models, animals were treated with anti-VEGF antibodies in combination with olaptesed pegol. As expected, VEGF blockade led to tumor hypoxia and elevated CXCL12 expression. Whereas intratumoral CD68 positive tumor associated macrophages (TAMs) were increased by VEGF blockade, the combination of olaptesed pegol and VEGF blockade markedly lowered TAM levels again. Macrophages are known to be proangiogenic (42), and they are actively recruited to the tumor microenvironment where they orchestrate resistance to anti-VEGF therapy (43). As a consequence, the combination of VEGF inhibition with olaptesed pegol also led to lower microvessel density in the tumor. Finally, adding olaptesed pegol to VEGF inhibition significantly prolonged the survival of the animals in both models compared with anti-VEGF therapy alone. In conclusion, inhibition of CXCL12 with olaptesed pegol decreased the recruitment of TAMs by VEGF blockage. Moreover, suppression of both, VEGF and CXCL12 pathways, was shown to synergize and reduce microvessel density which resulted in increased tumor control and prolonged overall survival in preclinical models of GBM.

Assessment of potential overlapping toxicities of olaptesed pegol and bevacizumab: No toxicology or drug-drug-interaction studies have been conducted for the combination of olaptesed pegol and bevacizumab. In order to enable a patient safety risk assessment, potential overlapping toxicities of VEGF inhibition with bevacizumab and of CXCL12 inhibition with olaptesed pegol are described in the following:

Safety pharmacology studies of olaptesed pegol demonstrate no effect on central nervous (CNS), cardiovascular (CV), or respiratory systems. No formal in vivo safety pharmacology studies were conducted with bevacizumab or the mouse anti-human VEGF monoclonal antibody A4.6.1; however, no effects on CNS and on the hERG tail current in vitro are anticipated as bevacizumab is a monoclonal antibody. Moreover, no treatment-related effects for other safety pharmacology endpoints, e.g. on blood pressure, ECG and respiration, were observed in repeat-dose toxicology studies of bevacizumab in cynomolgus monkeys. Further, olaptesed pegol and bevacizumab do not share organ toxicities based on data generated in repeated dose toxicity studies; both compounds were tested in cynomolgus monkeys as lead toxicology species. It should be mentioned for the sake of completeness that bevacizumab produced reversible, dose-dependent occurrence of physeal dysplasia secondary to the inhibition of blood vessel formation in long bone growth plates in nonclinical safety studies. As physeal dysplasia was only related to young animals with open growth plates, this pathological bone finding would only have clinical relevance for studies that enroll pediatric or adolescent patients. No adverse effect on bone growth plates has been seen with olaptesed pegol.

Concerning a potential genotoxic or carcinogenic effect of these compounds, experimental data demonstrate that olaptesed pegol is non-genotoxic; its carcinogenic effect is unknown

yet. As bevacizumab is a monoclonal antibody no genotoxic or carcinogenic effect is expected anyway.

Reproductive and developmental toxicity has not yet been fully investigated for olaptesed pegol. However, as evidenced from repeat-dose studies of up to 3 months duration employing mice and cynomolgus monkeys, olaptesed pegol produced no adverse effect on the male or female reproductive organs. Likewise, no specific studies in animals have been conducted for bevacizumab to evaluate the effect on fertility, embryo-fetal development, and peri-/postnatal development. A critical effect of bevacizumab was identified in repeat dose toxicity studies that have shown inhibition of the maturation of ovarian follicles and a decrease/absence of corpora lutea and associated decrease in ovarian and uterus weight as well as a decrease in the number of menstrual cycles in monkeys or rabbits. An adverse effect on female fertility is an expected effect due to VEGF inhibition by bevacizumab – the drug's mode of action. Moreover, bevacizumab was embryotoxic and teratogenic when administered to rabbits. IgGs are known to cross the placenta, and bevacizumab is anticipated to inhibit angiogenesis in the fetus. These effects, which are based on the critical role of angiogenesis in ovarian function and normal fetal development, are expected for bevacizumab. Potential embryo-fetal developmental toxicity is not investigated yet for NOX-A12.

Olaptesed pegol did not elicit a significant immunogenic response in animal studies while antibody formation against bevacizumab has been reported in nonclinical safety studies. An overlapping immunogenic response is not expected.

Both olaptesed pegol as well as bevacizumab demonstrated good local tolerability at application sites and therefore, no overlapping toxicity exists in this endpoint. Finally, for bevacizumab there is a range of other toxicity studies which would not indicate potential for overlapping toxicities. Overall, the above reasonings show that the intended combination therapy does not jeopardize a causality assessment of potential adverse events in relation to olaptesed pegol.

From the toxicological point of view, a non-clinical combination toxicology study is deemed not necessary to characterize overlapping toxicities. As deduced from published nonclinical safety data of bevacizumab, the risk of potential overlapping toxicities from olaptesed pegol + bevacizumab is negligible.

In conclusion, no overlapping toxicities have been identified (for details see Abbreviated Investigator's Brochure "MVASI® (Bevacizumab) VEGF Inhibitor to be Used in Combination with CXCL12 Inhibitor Olaptesed Pegol").

Animal models to combine inhibition of PD-1 and the CXCR4/CXCL12 axis in glioblastoma and other tumors: Glioblastoma is known to be an immunologically "cold" tumor characterized by a scarcity of tumor infiltrating lymphocytes (TILs). The bulk of infiltrating immune cells are macrophages and monocytes that are considered to have protumor and immunosuppressive effects (30). Recent studies suggest that myeloid cells play a critical role in glioblastoma immunosuppression. One preclinical study investigated the treatment effects of combination immunotherapy with anti-PD-1 and an inhibitor of the CXCL12/CXCR4 axis in an orthotopic, syngeneic murine glioma model. Combination therapy conferred a significant survival benefit compared to control and monotherapy arms. Mice that received combination therapy demonstrated immune memory and decreased populations of immunosuppressive tumor-

infiltrating leukocytes, such as monocytic myeloid-derived suppressor cells and microglia within the brain. Furthermore, combination therapy improved CD4+/CD8+ ratios in the brain as well as contributed to increased levels of pro-inflammatory cytokines (31).

The concept of combining olaptesed pegol with PD-1 inhibition in order to modulate the tumor microenvironment has been established *in vitro* and *in vivo*. It has been shown that olaptesed pegol enhanced the infiltration of immune effector cells into multicellular spheroids that mimicked a CXCL12-expressing tumor microenvironment. Moreover, olaptesed pegol synergized with PD-1 immune checkpoint blockade in a reporter-based T cell activation assay adapted to the spheroid format. *In vivo* proof of concept was achieved in a syngeneic mouse model for colorectal cancer that poorly responds to treatment with PD-1 inhibitor, where olaptesed pegol significantly enhanced the efficacy of anti-PD-1 therapy. This suggests that inhibition of CXCL12 by olaptesed pegol might be a promising strategy to overcome the resistance of solid tumors to checkpoint inhibitors (44). Our data is in line with other publications of observed synergy between inhibition of the CXCL12/CXCR4 axis and anti-PD-1 treatment in other cancer types such as pancreatic cancer (45), melanoma and colorectal cancer (46).

Assessment of potential overlapping toxicities of olaptesed pegol and pembrolizumab: No toxicology or drug-drug-interaction studies have been conducted for the combination of olaptesed pegol and pembrolizumab. In order to enable a patient safety risk assessment, potential overlapping toxicities of PD-1 inhibition with pembrolizumab and of CXCL12 inhibition with olaptesed pegol were evaluated.

Safety pharmacology studies of olaptesed pegol demonstrate no effect on central nervous (CNS), cardiovascular (CV), or respiratory systems. No formal *in vivo* safety pharmacology studies were conducted with pembrolizumab; however, no effects on CNS and on the hERG tail current *in vitro* are anticipated as pembrolizumab is a monoclonal antibody. Moreover, safety pharmacology endpoints for pembrolizumab were examined as part of the repeat-dose toxicity studies in cynomolgus monkeys. No treatment-related effects e.g., on ECG, general veterinary and physical examinations, body temperature, blood pressure, clinical observations, and histopathology of tissues were observed. Further, olaptesed pegol and pembrolizumab do not share organ toxicities based on data generated in repeated dose toxicity studies; both compounds were tested in cynomolgus monkeys as lead toxicology species.

Concerning a potential genotoxic or carcinogenic effect of these compounds, experimental data demonstrate that olaptesed pegol is non-genotoxic; its carcinogenic effect is unknown yet. As pembrolizumab is a monoclonal antibody no genotoxic or carcinogenic effect is expected anyway.

Reproductive and developmental toxicity has not yet been fully investigated for olaptesed pegol. However, as evidenced from repeat-dose studies of up to 3 months duration employing mice and cynomolgus monkeys, olaptesed pegol produced no adverse effect on the male or female reproductive organs. Likewise, no specific guideline-compliant reproductive and developmental toxicity studies in animals have been conducted for pembrolizumab to evaluate the effect on fertility, embryo-fetal development, and peri-/postnatal development. From repeat-dose toxicology studies it is known that there were no notable effects in the male and female reproductive organs in cynomolgus monkeys given pembrolizumab. Based on the mechanism of action of pembrolizumab, however, fetal harm may occur, and females of

reproductive potential should be advised of the potential risk to a fetus and to use effective method of contraception. This is because animal models link the PD-1/PD-L1 signaling pathway with maintenance of pregnancy through induction of maternal immune tolerance to fetal tissue. Blockade of PD-L1 signaling has been shown in murine models of pregnancy to disrupt tolerance to the fetus and to result in an increase in fetal loss. Further, human IgG4 are known to cross the placenta. Therefore, pembrolizumab has the potential to be transmitted from the mother to the developing fetus. There were no malformations related to the blockade of PD-1 signaling. Overall, potential risks of administering pembrolizumab during pregnancy include increased rates of abortion or stillbirth, as clearly indicated in the patient information of KEYTRUDA® (pembrolizumab). These effects, which are based on the mode of action, are expected for pembrolizumab. Potential embryo-fetal developmental toxicity is not investigated yet for olaptesed pegol.

Olaptesed pegol did not elicit a significant immunogenic response in animal studies while antibody formation against pembrolizumab has been reported in the nonclinical safety studies. For pembrolizumab there is also a non-GLP toxicity study in SCID mice which evaluated Fab-arm exchange of pembrolizumab *in vivo* and formation of hybrid antibodies. Induction of half molecules is typical for IgG4 antibodies. As expected for an IgG4/kappa isotype with a stabilizing SER228PRO sequence alteration in the Fc region, the mutation in the IgG4 hinge region of pembrolizumab prevented the formation of half molecules. Concerning Fab-arm exchange there is no potential for overlapping toxicity with olaptesed pegol. Overall, an overlapping immunogenic response is not expected.

Both olaptesed pegol as well as pembrolizumab demonstrated good local tolerability at application sites and therefore, no overlapping toxicity exists in this endpoint. Overall, the above reasonings show that the intended combination therapy does not jeopardize a causality assessment of potential adverse events in relation to olaptesed pegol.

From the toxicological point of view, a non-clinical combination toxicology study is deemed not necessary to characterize overlapping toxicities. As deduced from published nonclinical safety data of pembrolizumab, the risk of potential overlapping toxicities from olaptesed pegol and pembrolizumab is negligible.

In conclusion, no overlapping toxicities have been identified (for details see Abbreviated Investigator's Brochure "KEYTRUDA® (Pembrolizumab) PD-1 Inhibitor to be Used in Combination with CXCL12 Inhibitor Olaptesed Pegol").

6.4 Clinical Data

To date, the clinical program portfolio consists of two completed Phase I studies in healthy subjects, two completed Phase IIa studies, one in multiple myeloma patients, and one in patients with chronic lymphocytic leukemia, and one completed Phase I/II study in patients with pancreatic and colorectal cancer.

A first-in-human study in healthy male and female subjects examined the safety, tolerability, PK and pharmacodynamics (PD) of single ascending i.v. doses of olaptesed pegol ranging from 0.05 to 10.8 mg/kg. All administered doses were safe and well tolerated. Antibodies against the PEG moiety of olaptesed pegol (preexisting and treatment induced) were detected but did not affect PK or PD. Exposure increased super-proportionally with the dose. The dose

proportionality factor between the lowest and highest dose was 1.5 and 2.7 for C_{max} and AUC, respectively. The plasma half-life approximated 38 h up to a dose of 2.7 mg/kg but was prolonged at higher doses. In line with CXCL12 inhibition, olaptesed pegol induced a dose-dependent mobilization of white blood cells and hematopoietic stem cells into the peripheral blood (47). The second Phase I study was a repeated-dose study with daily intravenous dosing over 5 consecutive days. This regimen was chosen to mimic the standard G-CSF regimen for stem cell mobilization. The number of stem cells did not increase but rather a prolongation of mobilization was observed. 2 mg/kg daily over 5 days appeared to be safe and well tolerated, whereas liver transaminase elevations led to a protocol-defined discontinuation of the 4 mg/kg/d dose group. It is concluded that such a high and intensive dosing regimen is to be avoided (48).

In two single arm open label Phase IIa studies in MM and CLL, 56 patients were enrolled (28 for each indication). Patients were treated with olaptesed pegol in addition to a standard treatment of bortezomib and dexamethasone for patients with MM, and bendamustine and rituximab for CLL. The add-on treatment of olaptesed pegol to the standard therapy did not elicit any special adverse event pattern on top of the known adverse events of bortezomib, dexamethasone, bendamustine, and rituximab as standard therapy in the respective indications. In two CLL patients, tumor lysis syndrome was observed, one case mild and the other severe (49,50).

Two potential adverse events suspected from healthy volunteer and preclinical data were not observed in these patient cohorts, (i) increase of AST and ALT, as observed in the second phase I study in healthy patients after daily intravenous dosing over 5 consecutive days, and (ii) inhibition of plasmatic coagulation as observed in cynomolgus monkeys. Despite having advanced and often refractory disease, the MM patients had an overall response rate of 68%. Two patients (7%) achieved a complete response, five patients (18%) a very good partial response and twelve (43%) patients a partial response. Patients in the CLL study reached an overall response rate of 82% with four patients (14%) obtaining a complete response and an additional nineteen with a partial response (68%).

A Phase 1/2 study with olaptesed pegol in combination with pembrolizumab in patients with colorectal or pancreatic cancer to evaluate pharmacodynamic effects, safety and efficacy showed induction of immune response, stable disease in 25% of patients, and prolonged time on treatment vs. prior therapy for 35% of patients. The incidence and severity of the observed adverse events was in line with the expected adverse events in patients with metastatic colorectal cancer or metastatic pancreatic cancer and with treatment with pembrolizumab; there was no indication that olaptesed pegol would increase the incidence or the severity of the adverse events. Clinical responses were associated with Th1-like tissue reactivity upon CXCL12 inhibition; T cells showed aggregation and directed movement towards the tumor cells in responding tissues. Median progression-free survival was 1.87 months, overall survival was 39% at 6 months and 20% at 12 months. Three of the stable disease patients (15% of the study population) survived for more than a year (51).

Rationale for combining bevacizumab with olaptesed pegol in glioblastoma: There is broad and robust consensus that CXCL12 and VEGF have complementary and synergistic activity in vasculo- and angiogenesis in physiological and pathological conditions including (myocardial) ischemia or stroke (10-16). Over the past decades, this synergistic activity has

been consistently detected to have a major role in the (re)nourishment of various tumors before and in response to anti-tumor therapies (17-20).

In the setting of glioblastoma, the rationale for combining CXCL12 and VEGF inhibition is derived from the processes in the tumor microenvironment that are initiated by radiotherapy – the mainstay of glioblastoma treatment – which has been reported to cause blood vessel damage that may lead to additional tumor cell killing (21). Depletion of microvasculature following radiotherapy results in weakly perfused or non-perfused areas of tumor in which surviving cells respond with overexpression of hypoxia induced factor 1 alpha (HIF-1). HIF-1 then induces expression of a wide variety of genes such as VEGF, which promotes the process of angiogenesis, driving sprouting of new vessels from nearby vessels (22,23), and CXCL12, which promotes vasculogenesis by attracting two types of bone marrow-derived cell populations to build new blood vessels; endothelial progenitor cells via CXCR7 and pro-neovascularizing bone marrow-derived monocytes via CXCR4.

Kioi et al. (4) published data supporting the hypothesis that tumor irradiation sterilizes sufficient number of endothelial cells in and adjacent to the tumor to abrogate angiogenesis which forces the tumor to rely on the vasculogenesis pathway to rebuild blood vessels. However, others (24) suggested that the small fraction of endothelial cells surviving radiotherapy would be capable of responding to VEGF with angiogenesis-driven revascularization. Greenfield thus hypothesized that (VEGF-driven) angiogenesis and (CXCL12-driven) vasculogenesis are both active in glioblastoma following radiotherapy.

This implies that CXCL12 and VEGF might thus each play a part in treatment escape when the other pathway is inhibited. A complementary role for CXCL12 and VEGF in tumor revascularization is further supported by the more recently available information that CXCL12 and VEGF are expressed preferentially in different GBM tumor compartments (25,26).

Assessment of the relevance of CXCL12-driven vasculogenesis and VEGF-driven angiogenesis thus provide a rationale for the hypothesis that addition of VEGF inhibition has the potential to synergize with CXCL12 inhibition by olaptesed pegol: Both processes appear to contribute to the recurrence of glioblastoma tumors after radiotherapy, and they are complementarily active in different substructures of the tumor.

This escape mechanism might be the reason why in Phase 3 trials with the VEGF inhibitor bevacizumab in newly diagnosed glioblastoma patients no improved overall survival was observed (7,8); bevacizumab was therefore not approved for this indication. Of note, the reason for the EMA's refusal was the absence of established efficacy, as "overall, the toxicity of bevacizumab in this regimen and combination did not, in itself, raise major concerns" (EMA Annex: Scientific conclusions and grounds for refusal of the variation presented by the European Medicines Agency.

The fact that VEGF blockade is effectively bypassed by CXCL12 expression in glioblastoma (9) together with our own data showing that VEGF blockade synergized with olaptesed pegol in an animal model where this combination treatment reduced not only the number of pro-angiogenic macrophages and the microvessel density but also prolonged overall survival (27) warrant the testing of this combination in a clinical study.

Rationale for combining pembrolizumab with olaptesed pegol in glioblastoma: The rationale for combining CXCL12 and PD-1 inhibition is derived from the lack of response to anti-PD-1

treatment in phase 3 clinical studies of anti-PD-1 therapy in glioblastoma, administered concurrently with standard of care radiotherapy or chemoradiotherapy, where no prolongation of PFS or OS could be noted (CheckMate 498, NCT02617589 and CheckMate 548, NCT02667587). This can be explained by the fact that glioblastoma is an immunologically "cold" tumor characterized by a scarcity of tumor infiltrating lymphocytes (TILs). Macrophages and monocytes compose the bulk of infiltrating immune cells and are considered to have protumor and immunosuppressive effects (30) and inhibition of their influx into the tumor is therefore considered a valuable target for combination therapy with immune checkpoint inhibitors. In fact, the combination of anti-CXCR4 with anti-PD-1 showed promising results in a preclinical model conferring a significant survival benefit (31). Interestingly, this drug combination led to a decreased density of immunosuppressive myeloid cell populations, more favorable CD8+/CD4+ T cell ratios and increased production of pro-inflammatory cytokines (31). Therefore, targeting the myeloid cell compartment is expected to convert what is considered the "cold" tumor microenvironment of glioblastoma to a "hot" phenotype. We observed such a phenomenon also clinically in one of our patients where we were able to investigate tumor tissue before and under olaptesed pegol therapy due to re-resection. Here, endothelial cells stained positive for CXCL12 before but not during olaptesed pegol treatment and almost all tumor cells were negative for the proliferation marker Ki67 during treatment. A significant infiltration of CD8+ T cells was observed during olaptesed pegol therapy, and most of these T cells were proliferating. These data together with the very good safety profile of the combination of olaptesed pegol and pembrolizumab in colorectal and pancreatic cancer patients (SNOXA12C401, Keynote-559, NCT03168139) as well as first pharmacodynamic data – the shift to a more inflammatory tumor microenvironment in half of the patients - warrants further investigation of this combination in glioblastoma patients.

6.5 Study Design Rationale

This study is conducted to assess safety of ascending doses of olaptesed pegol when administered concomitantly to radiation therapy in this patient population.

In addition, the study is expected to explore the benefit of olaptesed pegol on progression-free survival when administered in addition to irradiation and to provide an estimate of the effect on overall survival.

This study is expected to advance the understanding of the important role that CXCL12 plays in neovascularization by recruiting endothelial and other bone marrow-derived pro-angiogenic cells through a CXCR4- and CXCR7-dependent mechanism. Notably, irradiation further increases CXCL12 expression (4,5). This study will thus explore the potential of inhibiting the CXCL12-driven influx of bone marrow-derived repair cells to irradiated glioblastoma and provide data on safety, pharmacokinetics and efficacy of olaptesed pegol when administered concomitantly to radiation therapy. More specifically, the data will be used to educate the further clinical development of olaptesed pegol.

Combination with VEGF inhibition: The efficacy of bevacizumab in combination with radiotherapy (+ temozolomide) is limited. In two Phase 3 studies (AVAglio, (7) and RTOG 0825, (8)), bevacizumab failed to show a beneficial effect on overall survival and it is clear that bevacizumab alone (i.e. in combination with radiotherapy) does not provide a significant clinical benefit for newly diagnosed glioblastoma patients, but there is a good rationale that

combination of the anti-angiogenic approach with CXCL12 inhibition can be an efficacious treatment for this patient population. There is

(i) broad and robust consensus that, in general, CXCL12 and VEGF have complementary and synergistic activity in vasculo- and angiogenesis;

(ii) evidence that both CXCL12 and VEGF play a part in treatment escape mechanism initiated by processes in the tumor microenvironment induced by radiotherapy of glioblastoma*;* and

(iii) data showing that suppression of both, VEGF and CXCL12 pathways, synergize and reduce microvessel density which leads to increased tumor control in two animal models of glioblastoma.

Combination with PD-1 checkpoint inhibition: The efficacy of anti-PD-1 in combination with radiotherapy (+ temozolomide) is limited. In two Phase 3 studies (CheckMate 498 and CheckMate 548), anti-PD-1 therapy concurrently with standard of care radiotherapy or chemoradiotherapy with temozolomide failed to show clinically meaningful effects, but there is a good rationale that combination immunotherapy of PD-1 inhibition and CXCL12 inhibition can be an efficacious treatment for this patient population. There is

(i) preclinical data showing synergy of inhibition of the CXCL12/CXCR4 signaling and anti-PD-1 treatment in a glioma model. In addition, CXCL12/CXCR4 inhibition has been shown to synergize with PD-1 inhibition in several other oncological models;

(ii) preliminary clinical data from the GLORIA study showing signs of immune cell activation in the tumor microenvironment of a glioblastoma patient on olaptesed pegol therapy;

(iii) established safety for the combination of olaptesed pegol in combination with pembrolizumab from a Phase 1/2 study as well as first pharmacodynamic data – a shift to a more inflammatory tumor microenvironment in half of the patients – in colorectal and pancreatic cancer.

Evaluation of the combination of VEGF- and CXCL12 inhibition as well as PD-1- and CXCL12 inhibition in this clinical study is therefore warranted.

6.6 Benefit / Risk Assessment

In this Phase 1/2 study, patients with newly diagnosed GB and unmethylated MGMT promoter will be treated with olaptesed pegol in combination with external-beam radiation therapy (EBRT) to explore safety/tolerability and efficacy of the combination with the aim to educate the design of subsequent studies. In an expansion group patient with newly diagnosed GB and unmethylated MGMT promoter will be treated with olaptesed pegol in combination with bevacizumab and EBRT to explore safety/tolerability and efficacy of the combination.

The risk of harm by study-specific procedures, i.e. venipuncture to collect blood samples, and subcutaneous implantation of a central venous port to enable protracted intravenous infusion (PVI) is considered acceptable for this patient population. Continuous infusions are then delivered using small light, portable and noiseless devices, which in many other indications are preferred over bolus infusion by patients and physicians (e.g. 5-FU in colorectal or esophageal cancer).

Radiation treatments are scheduled according to standard of care for glioblastoma. No additional radiological assessments beyond normal standard of care are planned. Thus,

weighing the available data, the level of risk for relevant complications is minimally increased in the tightly controlled setting of this study. As glioblastoma patients with unmethylated MGMT promoter do not clearly profit from combination of radiotherapy with temozolomide (35), a deviation from this standard of care radio-chemotherapy by omitting temozolomide treatment is justifiable in the patient population. Previous trials (52,53) have already been performed according to this principle without TMZ in the experimental arm. Continuous infusions are then delivered using small light, portable and noiseless devices, which in many other indications are preferred over bolus infusion by patients and physicians (e.g. 5-FU in colorectal or esophageal cancer).

In previous clinical studies, treatment with olaptesed pegol was well tolerated in patients with multiple myeloma and with chronic lymphocytic leukemia at similar or even higher cumulative weekly doses and on top of standard anti-cancer treatment with bortezomib/dexamethasone and bendamustine/rituximab, respectively; in addition, the observations in the study in patients with colorectal and pancreatic cancer do not suggest safety issues for weekly doses of up to 600 mg for two weeks. No specific safety concern or signal was identified for olaptesed pegol. The patients will be treated and monitored at study centers that have extensive experience with adequate management of their underlying medical condition and with monitoring and reviewing adverse effects in the context of clinical studies. Visits to the study center are scheduled at least every week, and laboratory values will be closely monitored.

With current standard of care, treatment with surgical resection, chemotherapy, and radiotherapy is followed by tumor recurrence and results in less than 10% of patients reaching 5-year survival (1). Animal studies suggest that radiation-induced devascularization is efficiently restored by vasculogenesis after recruitment of bone marrow–derived cells which leads to rapid recurrence of the tumor (4). Recently, the importance of the vascular scaffold in gliomas was examined further, implying that angiogenesis and vasculogenesis might in fact have complementary function in neoplasms.

The use of bevacizumab is not standard of care in glioblastoma patients but was assessed in the randomized, double-blind, placebo controlled AVAglio Phase 3 study of bevacizumab, temozolomide and radiotherapy in patients with newly diagnosed glioblastoma. In this study, an improvement of median PFS was reported in the bevacizumab arm, but there was no clinically relevant effect on OS. The most frequent toxicity associated with the bevacizumab treatment group were gastrointestinal (nausea, constipation, vomiting), alopecia, fatigue, thrombocytopenia, headache and hypertension. No new safety signals with bevacizumab were observed in the AVAglio study, but there was a higher incidence of Grade 3 and 4 AEs, SAEs, non-progression deaths, and discontinuations of treatment in the bevacizumab group. Arterial thromboembolic events, mainly ischemic strokes, were observed at a slightly higher incidence rate in this trial (5.0%) than in previous bevacizumab trials (up to 3.8% in combination with different chemotherapies), and higher than in the placebo group (1.6%) (7). Although the EMA concluded that in the absence of established efficacy, the benefit-risk balance does not warrant approval for this indication, they also state that *“overall, the toxicity of bevacizumab in this regimen and combination did not, in itself, raise major concerns”*, including when bevacizumab and radiotherapy were administered simultaneously. None of the toxicities of bevacizumab that are listed as “Special warnings and precautions for use” in the EMA Summary of Product Characteristics of bevacizumab or as “Warnings and Precautions” in the FDA Prescribing

Information of the marketed product have been observed in clinical studies with olaptesed pegol.

The use of pembrolizumab in glioblastoma is not standard of care in glioblastoma patients, but there were several clinical trials with PD-1 inhibitors. Treatment of patients with nivolumab or pembrolizumab in addition to standard of care did not show relevant benefit for the patients (28,29,54,55). The most frequent toxicities associated with the pembrolizumab treatment group were fatigue, pruritus, diarrhea, asthenia, hypothyroidism, arthralgia, nausea, rash and immune-mediated and infusion reactions. Grade 3 treatment-related adverse events occurred in approximately one tenth of patients, with the most common being increased gamma-glutamyltransferase and pneumonitis. Three patients had grade 4 treatment-related adverse events, and one patient died as a result of a treatment-related adverse event of pneumonia. Overall, severe toxicity occurred at a low rate, especially in light of all the cytotoxic chemotherapy agents that many patients in this population had previously received (55).

The combination of olaptesed pegol and pembrolizumab in colorectal and pancreatic cancer patients was generally well tolerated even by heavily pretreated patients with more than four previous lines of therapy (28,56). Toxicities were readily manageable with abdominal pain being the most frequent followed by edema peripheral, fatigue, constipation and tumor pain. Overall tolerability was very good with 83% of adverse events being Grade 1 and 2. Only low incidences of Grade 3 events mainly of gastrointestinal disorders were reported which all except one were not related to treatment with olaptesed pegol. No Grade 4 event was reported. One patient had a fatal event, general physical health deterioration. The event is considered neither to be related to treatment with olaptesed pegol and nor with pembrolizumab. This death is considered to be due to disease under study. Generally, the frequency and severity of adverse events are in the range to be expected in patients with metastatic colorectal cancer or metastatic pancreatic cancer and under treatment with pembrolizumab. It can therefore be concluded that treatment with olaptesed pegol did not result in additional relevant toxicities (SNOXA12C601, Keynote-559, NCT03168139).

The risk of a chemical interaction between olaptesed pegol and monoclonal antibodies such as bevacizumab, but also rituximab and pembrolizumab – which both have previously been combined with olaptesed pegol – is considered very unlikely. As olaptesed pegol is a highly hydrophilic molecule with negligible binding to plasma proteins, it is not expected to influence the plasma levels of bevacizumab and vice versa. In this study, plasma levels of olaptesed pegol will be continuously monitored to ensure that no unexpected changes in plasma levels are observed. The potential benefit of combining olaptesed pegol with bevacizumab or pembrolizumab is to target different elements in the supportive TME and by that prolong patients' life.

In summary, taking into account (i) the so far very benign safety profile of olaptesed pegol alone and in combination with standard cancer treatments in patients with hematological malignancies, (ii) the high medical need in the target patient population and (iii) the sound preclinical rationale for the combination of CXCL12 inhibition with EBRT as well as with VEGF and PD-1 inhibition in brain tumor models, the expected progress for this therapeutic area and the potential benefit for individual patients outweigh the moderate burden of risk to which the participating patients will be exposed.

7. Study Objectives and Endpoints

7.1 Primary Objective

- To investigate the safety of either olaptesed pegol combination with radiation therapy or olaptesed pegol combination with radiation therapy and bevacizumab or pembrolizumab in patients with newly diagnosed glioblastoma of unmethylated MGMT promoter status

7.2 Primary Endpoint

- Safety (adverse events)

7.3 Secondary Objectives

- To explore the efficacy of olaptesed pegol in combination with radiation therapy on patients with glioblastoma
- To investigate the efficacy of olaptesed pegol in combination with radiation therapy and bevacizumab or pembrolizumab in patients with glioblastoma of unmethylated MGMT promoter status
- To investigate the pharmacokinetics of olaptesed pegol during continuous administration
- To monitor symptoms (NANO) and QoL

7.4 Secondary Endpoints

- PFS at 6 months (PFS-6)
- Median progression-free survival (mPFS)
- Median overall survival (mOS)
- Tumor vascularization as per vascular MRI scans at baseline and following 2, 4, and 6 months
- Topography of recurrence
- Determination of maximum tolerated dose (MTD)
- Definition of recommended Phase 2 dose (RP2D)
- Olaptesed pegol plasma levels at steady state
- NANO assessment
- Quality of Life

8. Investigational Plan

8.1 Overall Study Design

This is a Phase 1/2 multi-center, open label, uncontrolled, dose-escalation clinical study evaluating the safety and efficacy of olaptesed pegol in combination with a radiation therapy in patients with glioblastoma consisting of

- a screening period of up to 28 days duration, to ensure that the patient is eligible for the study.
- a treatment period of 26 weeks with 6 weeks combination therapy of olaptesed pegol with radiation therapy followed by 20 weeks treatment with olaptesed pegol alone or of 26 weeks with 6 weeks combination therapy of olaptesed pegol with either bevacizumab or pembrolizumab and radiation therapy followed by 20 weeks combination treatment with olaptesed pegol and either bevacizumab or pembrolizumab. All visits will take place at the site. Patients with disease progression during the 26-week treatment period will continue treatment with all assessments if deemed appropriate by the investigator.
- a follow-up period of 24 months calculated from first dose with assessments every 3 months after end of treatment.

The study is completed with the last visit of last patient.

Details on all study visits can be found in the time and events schedule in Section 2.1.

8.2 Choice of Control Groups

The purpose of this Phase 1/2 study is to obtain first, exploratory information on the safety and efficacy of olaptesed pegol in combination with radiation therapy. The inclusion of a control group is not deemed appropriate given the small number of patients, which would not allow for a meaningful statistical evaluation.

8.3 Selection of Doses

8.3.1 Olaptesed pegol

Olaptesed pegol will be administered as a fixed dose of 200, 400, or 600 mg weekly by continuous i.v. infusion. The selection of the dosing regimen is supported by safety and efficacy considerations. The proposed continuous infusion of 200 mg/week is expected to result in pharmacologically relevant mean plasma levels of olaptesed pegol (approx. 1.5 µM at steady state), considered to be the minimal dose expected to disrupt CXCL12 mediated migration while leading to no or only low cell mobilization from the bone marrow.

An increase in CXCL12 plasma levels has been reported for glioblastoma patients in response to radiation therapy (57) which may increase the required dose of olaptesed pegol. Therefore, escalation to higher doses of 400 and 600 mg/week is planned to result in mean olaptesed pegol plasma levels of approx. 3.1 and 4.6 µM at steady state, respectively.

Simulations show that the respective steady state plasma concentrations are reached with continuous infusions after 7 to 10 days. In order to reach the expected steady state

concentrations after approx. 24h, doses of 70, 160 and 230 mg will be administered for the first 24 h resulting in expected plasma concentrations of approx. 1.3 µM, 3.0 µM and 4.4 µM, respectively.

Dosing rationale – clinical safety:

Due to the benign safety profile observed thus far, dose adjustment on body weight has already been abandoned in the last clinical study with olaptesed pegol in patients with pancreatic or colorectal cancer. For glioblastoma patients, the considerations are as follows:

The body weight distribution of a population of 1,259 glioblastoma patients was 78.9 ±17.5 kg (58), with calculated 2nd and 98th percentiles of 44 and 114 kg, respectively. Assuming this body weight distribution in future patients, a fixed dose

- of 200 mg olaptesed pegol would correspond to weight adjusted doses ranging from 1.8 to 4.5 mg/kg with a mean of 2.5 mg/kg;
- of 400 mg olaptesed pegol would correspond to weight adjusted doses ranging from 3.5 to 9.1 mg/kg with a mean of 5.1 mg/kg and;
- of 600 mg olaptesed pegol would correspond to weight adjusted doses ranging from 5.2 to 13.6 mg/kg with a mean of 7.6 mg/kg.

Dose limiting toxicities in healthy subjects, i.e. elevated ALT and AST, were observed at daily i.v. doses of 4 mg/kg (plasma levels between 10 and 20 µM at steady state), whereas daily i.v. doses of 2 mg/kg (plasma levels between 5 and 10 µM at steady state) were generally safe and well tolerated. A dosing regimen that results in continuous plasma levels of 10 µM or more is thus to be avoided. In two previous studies in patients with CLL and MM, twice weekly doses of 4 mg/kg, corresponding to a cumulative weekly dose of approx. 600 mg, did not add toxicity on top of standard cancer therapy. Thus, even the proposed highest flat fixed dose of 600 mg/week, is not exceeding the weekly dose in the MM study determined to be safe. Even for patients with an extremely low body weight of 44 kg where doses of 200, 400 and 600 mg/week correspond to a weekly dose of 4.5, 9.1 and 13.6 mg/kg, respectively (2.8, 5.6 and 8.3 µM at steady state), no safety issue is thus to be expected.

The concentration profile of olaptesed pegol in plasma was simulated from plasma elimination parameters determined after single i.v. dose administration of 2.7 mg/kg to healthy volunteers with an average weight of 77 kg. In order to estimate the systemic exposure of patients within the weight range 44 – 114 kg, it was roughly assumed that V_d is directly proportional to body weight (Table 8-1). Simulated concentration profiles for a 79 kg-patient are shown in Figure 8-1, steady state plasma concentrations of about 1.6 µM, 3.2 µM and 4.6 µM, respectively, are reached after approx. 1 to 2 days.

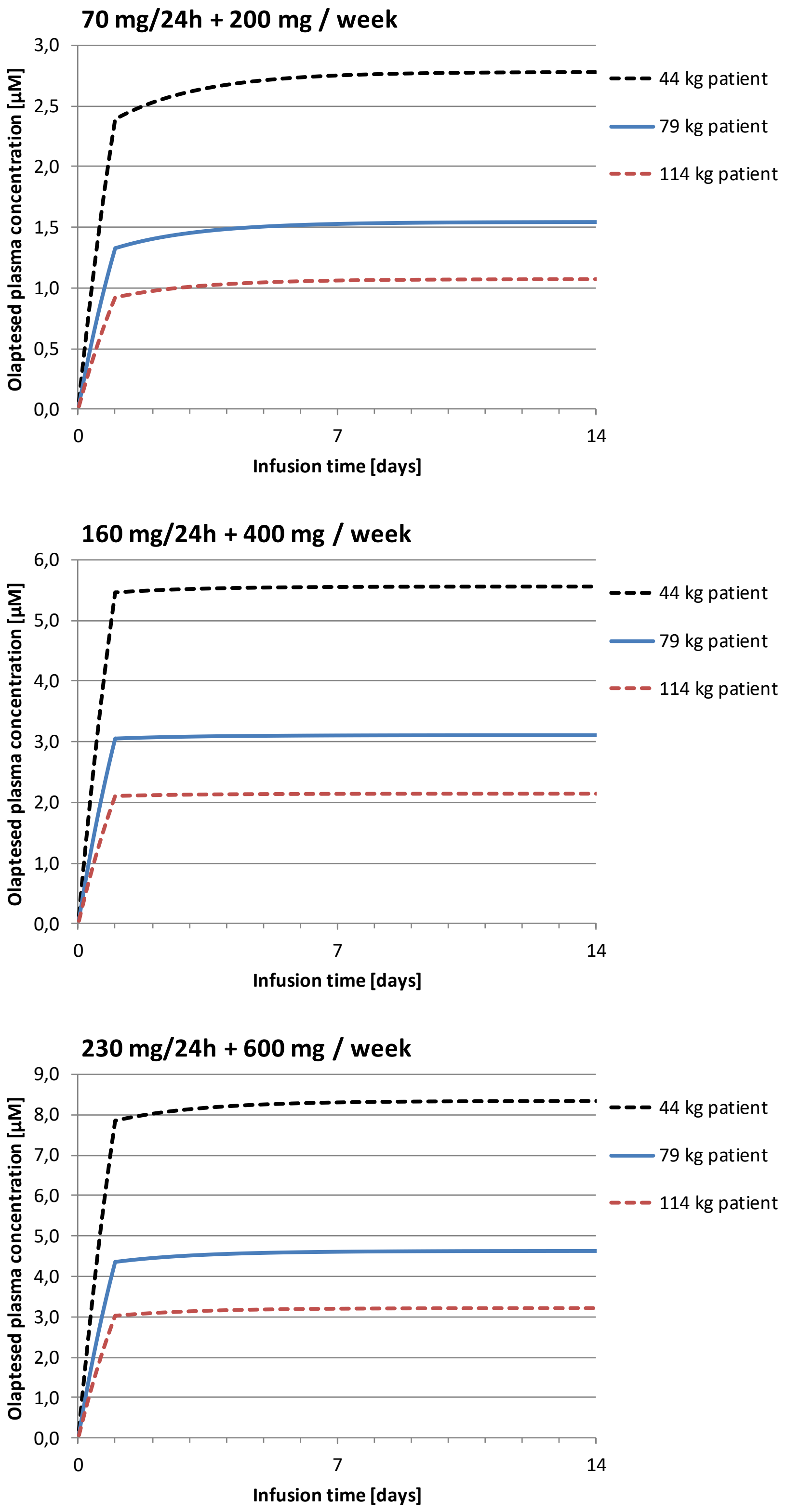

Figure 8-1: Simulation of olaptesed plasma concentration for continuous infusion of 200, 400 and 600 mg/week olaptesed pegol

Table 8-1: Expected systemic exposure of GB patients continuously treated with iv olaptesed pegol at an infusion rate of 200, 400 and 600 mg/week

Total dose applied	200 mg/week			400 mg/week			600 mg/week		
**Percentile**	**2nd**	**50th**	**98th**	**2nd**	**50th**	**98th**	**2nd**	**50th**	**98th**
**Body weight (BW) [kg]**	44	79	114	44	79	114	44	79	114
**BW adjusted V_d* [L]**	1.6	2.9	4.2	1.6	2.9	4.2	1.6	2.9	4.2
**BW adjusted dose [mg/kg/week]**	4.5	2.5	1.8	9.1	5.1	3.5	13.6	7.6	5.2
**Plasma steady-state concentration (simulated) [µM]**	2.8	1.5	1.1	5.6	3.1	2.1	8.3	4.6	3.2

* estimated by direct proportionality of V_d to body weight

Dosing rationale – human pharmacodynamics:

According to the proposed mode of action in glioblastoma – disruption of the CXCL12 gradient to inhibit recruitment of bone marrow-derived repair cells to the irradiated tumor – a steady state plasma level should be reached that abolishes the migration of the repair cells but leads to no or only marginal mobilization of white blood cells from the bone marrow. Such low-grade and short mobilization of white blood cells occurs at peak plasma levels of approx. 2 µM, as they are reached after an i.v. bolus of 1 mg/kg olaptesed.

The experimentally determined production rate of CXCL12 in healthy human subjects is approx. 26 nM/h in plasma (59). In order to achieve a pharmacologically relevant CXCL12 inhibition, olaptesed pegol has to be provided at a rate at least similar to the CXCL12 production. At the dose of 200 mg/week an average infusion rate of approx. 27 nM/h olaptesed pegol is achieved in a subject with an approximate plasma volume of 3 L, for the dose of 400 mg/week a rate of 55 nM/h and for the dose of 600 mg/week a rate of 81 nM/h.

Dosing rationale – non-clinical safety:

The expected olaptesed pegol plasma levels at steady state in this study are approx.1.6 µM, 3.2 µM and 4.6 µM for the 200, 400 and 600 mg/week dose group. These levels are substantially lower than the approx. 30 µM maximal concentration observed at the no observed adverse effect level (NOAEL) in the mouse and approx. 47 µM in the cynomolgus monkey 2-week toxicity studies. In the 3-month studies the plasma levels reached approx. 55 µM in mice at the NOAEL and approx. 4.2 µM in monkeys. Of note, even at doses about NOAEL, no target organ toxicity was observed. The major treatment-related findings in mice or monkeys in terms of laboratory diagnostic parameters were mild secondary effects on hematology and blood biochemistry parameters. Further, a slight, reversible increase in body weight was observed, as well as organ weight increases of liver and spleen with no corresponding toxicologically relevant pathological finding. Only at extreme doses leading to plasma levels of up to 220 µM, subcutaneous hemorrhage and hematomas were observed in some monkeys associated with prolonged coagulation time.

Dosing rationale – non-clinical pharmacology:

In a rat glioblastoma model (5), a dose of 10 mg/kg q2d was found to be efficacious. Olaptesed pegol was administered subcutaneously to achieve a continuous exposure rather than sharp peaks and troughs that are typical for i.v. bolus administration. Due to the low subcutaneous bioavailability and faster plasma elimination of olaptesed pegol in rat and a given dose proportionality, mean plasma levels between approx. 2 and 4 µM at trough and peak can be estimated for this dose at steady state. [redacted].

Definition and selection of RP2D:

The RP2D is defined as the highest dose tested that is declared safe and tolerable by the DSMB, taking into account data from four weeks of treatment at that dose. [redacted]

[redacted]

[redacted]

[redacted]

[redacted]

[redacted]

[redacted]

[redacted]

[redacted]	[redacted]	[redacted]	[redacted]
[redacted]	[redacted]	[redacted]	[redacted]
[redacted]	[redacted]	[redacted]	[redacted]
[redacted]	[redacted]	[redacted]	[redacted]
[redacted]	[redacted]	[redacted]	[redacted]
[redacted]	[redacted]	[redacted]	[redacted]
[redacted]	[redacted]	[redacted]	[redacted]

[redacted]

[redacted]

[redacted]

[redacted]

[redacted]

[redacted]

[redacted]	[redacted]		
	[redacted]	[redacted]	[redacted]
[redacted]	[redacted]	[redacted]	[redacted]
[redacted]	[redacted]	[redacted]	[redacted]
[redacted]	[redacted]	[redacted]	[redacted]
[redacted]	[redacted]	[redacted]	[redacted]

8.3.2 Bevacizumab

Patients will receive a 6-weeks combination therapy of 600 mg/week olaptesed pegol with 10 mg/kg q2w bevacizumab and radiation therapy followed by 20 weeks combination treatment with 600 mg/week olaptesed pegol and bevacizumab.

The chosen dose regimen of 10 mg/kg body weight every 2 weeks (q2w) was already used in Phase 2 studies in glioblastoma patients (61,62), which finally led to the choice of this dosing regimen for two Phase 3 glioblastoma studies.

In the first, AVAglio Phase 3 study, patients received concurrent radiotherapy and temozolomide in combination with bevacizumab. The last concurrent doses of temozolomide and bevacizumab were administered on the day of the last dose of radiotherapy. The concurrent-therapy phase was followed by a 28-day treatment break. In the maintenance phase, patients received temozolomide plus bevacizumab for six 4-week cycles. In the monotherapy phase, bevacizumab was continued every 3 weeks until the disease progressed, or unacceptable toxic effects developed (7).

In the second, RTOG 0825 Phase 3 study, bevacizumab was administered starting at week 4 of radiotherapy, until disease progression, severe treatment-related toxicity, or completion of adjuvant therapy (maximum number of doses, 24 over 12 cycles). Maintenance treatment with temozolomide began 4 weeks after the completion of radiotherapy. Treatment was planned for 6 cycles with the option of extension to a total of 12 cycles if there were no or only low-grade adverse events and there was evidence of continued benefit (8).

Further, the dose of 10 mg/kg q2w alone and in combination with several anti-cancer treatments has been investigated and / or approved. In the targeted indication of newly diagnosed glioblastoma, the dose of 10 mg/kg q2w might have failed in terms of efficacy to shown improvement on overall survival, but the toxicities of bevacizumab are well known and described. In the US, bevacizumab is approved for the treatment of glioblastoma with progressive disease in adult patients following prior therapy as a single agent with a recommended dose of 10 mg/kg q2w.

In both the EU and the US, the dosing regimen of 10 mg/kg q2w is used for most indications, with some exceptions where the recommended dose is 15 mg/kg q3w.

Therefore, the dose of 10 mg/kg q2w is deemed to be the most appropriate dose for this study.

8.3.3 Pembrolizumab

Patients will receive a 6-weeks combination therapy of 600 mg/week olaptesed pegol with 200 mg every 3 weeks pembrolizumab and radiation therapy followed by 20 weeks combination treatment with 600 mg/week olaptesed pegol and 200 mg every 3 weeks pembrolizumab.

The recommended dose of KEYTRUDA in adults is 200 mg every 3 weeks. The dose is approved in the US and in the EU for treatment of several types of cancer.

Elderly patients: No dose adjustment is necessary in patients ≥ 65 years.

Renal impairment: No dose adjustment is needed for patients with mild or moderate renal impairment. KEYTRUDA has not been studied in patients with severe renal impairment.

Hepatic impairment: No dose adjustment is needed for patients with mild hepatic impairment. KEYTRUDA has not been studied in patients with moderate or severe hepatic impairment.

8.4 Selection of Study Population

The study population will consist of a representative group of male and female patients aged 18 and older with newly diagnosed glioblastoma with unmethylated MGMT promoter who underwent only a biopsy or a partial tumor resection. It is planned to screen up to 24 patients of which up to 18 patients will be enrolled in the study.

After determination of the RP2D, 6 additional patients with newly diagnosed glioblastoma with unmethylated MGMT promoter who underwent complete, incomplete or no tumor resection will be enrolled and receive treatment with olaptesed pegol in combination with radiotherapy and bevacizumab (Arm A).

Further 6 patients with newly diagnosed glioblastoma with unmethylated MGMT promoter who underwent complete tumor resection will be enrolled and receive treatment with olaptesed pegol in combination with radiotherapy (Arm B).

Another 6 patients with newly diagnosed glioblastoma with unmethylated MGMT promoter who underwent complete or only incomplete tumor resection will be enrolled and receive treatment with olaptesed pegol in combination with radiotherapy and pembrolizumab (Arm C).

The investigator must ensure that all patients being considered for the study meet the following inclusion and exclusion criteria. No additional exclusions should be applied by the investigator, in order that the study population will be representative of all eligible patients. Patient selection is to be established by checking through all inclusion/exclusion criteria at screening and prior to enrollment. Deviation from any entry criterion excludes a patient from enrollment into the study. Re-enrollment of patients is not allowed.

Criteria must be reviewed prior to first treatment to ensure proper eligibility for enrollment. Withdrawal criteria (see Section 8.4.3) must be reviewed at each study visit until last treatment.

8.4.1 Inclusion Criteria

8.4.1.1 Inclusion Criteria for Dose Escalation Cohorts

Patients meeting the following criteria will be considered for inclusion into the study:

1. Written informed consent
2. Age ≥18 years
3. Patient agreement to diagnostic and scientific work-up of glioblastoma tissue obtained during the preceding surgery or biopsy (e.g., MGMT promoter analysis, cytogenetic markers such as IDH-1 mutations, etc.)
4. Patient agrees to subcutaneous port implantation
5. Newly diagnosed, histologically confirmed, supratentorial WHO grade IV glioblastoma
6. Status post biopsy or incomplete resection (detectable residual tumor as per postoperative T1-weighted, contrast-enhanced MRI scan)
7. Unmethylated MGMT promoter status

8. Maximum Eastern Cooperative Oncology Group (ECOG) score 2
9. Estimated minimum life expectancy 3 months
10. Stable or decreasing dose of corticosteroids during the week prior to inclusion
11. The following laboratory parameters should be within the ranges specified:
    - Total bilirubin ≤ 1.5 x upper limit normal (ULN)
    - Creatinine ≤ 1.5 x ULN or glomerular filtration rate ≥ 60 mL/min/1.73m²
    - ALT (alanine transaminase) ≤ 3 x ULN
    - AST (aspartate transaminase) ≤ 3 x ULN
12. Female patients of child-bearing potential must have a negative serum pregnancy test within 21 days prior to enrollment and agree to use a highly effective method of birth control (failure rate less than 1% per year when used consistently and correctly such as contraceptive implants, vaginal rings, sterilization, or sexual abstinence) during and for 3 months following last dose of drug (more frequent pregnancy tests may be conducted if required per local regulations)
13. Male patients must use an effective barrier method of contraception during study and for 3 months following the last dose if sexually active with a FCBP

8.4.1.2 Inclusion Criteria for Expansion Group

Patients meeting the following criteria will be considered for inclusion into the study:

1. Written informed consent
2. Age ≥ 18 years
3. Patient agreement to diagnostic and scientific work-up of glioblastoma tissue obtained during the preceding surgery or biopsy (e.g., MGMT promoter analysis, cytogenetic markers such as IDH-1 mutations, etc.)
4. Patient agrees to subcutaneous port implantation
5. Newly diagnosed, histologically confirmed, supratentorial WHO grade IV glioblastoma
6. a) Status post biopsy or incomplete (detectable residual tumor as per postoperative T1-weighted, contrast-enhanced MRI scan) or complete resection (Arm A)

   OR

   b) Status post complete resection (Arm B)

   OR

   c) Status post complete or incomplete resection (circumscribed enhancing tumor ≤ 5.0 cm in largest diameter as per postoperative T1-weighted, contrast-enhanced MRI scan) (Arm C)
7. Unmethylated MGMT promoter status
8. Maximum Eastern Cooperative Oncology Group (ECOG) score 2
9. Estimated minimum life expectancy 3 months
10. Stable or decreasing dose of corticosteroids during the week prior to inclusion
11. The following laboratory parameters should be within the ranges specified:
    - Total bilirubin ≤ 1.5 x upper limit normal (ULN)

- Creatinine ≤ 1.5 x ULN or glomerular filtration rate ≥ 60 mL/min/1.73m²
- ALT (alanine transaminase) ≤ 3 x ULN
- AST (aspartate transaminase) ≤ 3 x ULN

12. Female patients of child-bearing potential must have a negative serum pregnancy test within 21 days prior to enrollment and agree to use a highly effective method of birth control (failure rate less than 1% per year when used consistently and correctly such as contraceptive implants, vaginal rings, sterilization, or sexual abstinence) during and for 3 months (6 months Arm A, 4 months Arm C) following last dose of drug (more frequent pregnancy tests may be conducted if required per local regulations)
13. Male patients must use an effective barrier method of contraception during study and for 3 months (6 months Arm A, 4 months Arm C) following the last dose if sexually active with a FCBP

8.4.2 Exclusion Criteria

8.4.2.1 Exclusion Criteria for Dose Escalation Cohorts

A patient will not be eligible for inclusion if any of the following criteria applies during screening or prior to enrolment:

1. Inability to understand and collaborate throughout the study or inability or unwillingness to comply with study requirements
2. Participation in any clinical research study with administration of an investigational drug or therapy within 30 days from screening visit or observation period of competing studies
3. Contra-indication or known hypersensitivity to MRI contrast agents, olaptesed pegol or polyethylene glycol
4. Cytostatic therapy (chemotherapy) within the past 5 years
5. History of other cancers (except for adequately treated basal or squamous cell skin cancer, in situ cervical cancer, or other cancer from which the patient was disease-free for ≥ 5 years)
6. Clinically significant or uncontrolled cardiovascular disease, including
   - Myocardial infarction in the previous 12 months
   - Uncontrolled angina
   - Congestive heart failure (New York Heart Association functional classification of ≥2)
   - Diagnosed or suspected congenital long QT syndrome
   - QTc prolongation on an electrocardiogram prior to entry (>470 ms)
   - Uncontrolled hypertension (blood pressure ≥ 160/95 mmHg)
   - Heart rate <50/min on the baseline electrocardiogram
   - History of ventricular arrhythmias of any clinically significant type (such as ventricular tachycardia, ventricular fibrillation or torsades de pointes)
7. Prior radiotherapy to the head
8. Any other previous or concomitant experimental glioblastoma treatments
9. Placement of Gliadel® wafer, seeds, or ferromagnetic nanoparticles

10. Pregnancy or lactation
11. Uncontrolled intercurrent illness including, but not limited to ongoing or active infection, chronic liver disease (e.g., cirrhosis, hepatitis), diabetes mellitus, or subjects with either of the following: fasting blood glucose (FBG defined as fasting for at least 8 hours) ≥ 200 mg/dL (7.0 mmol/L), or HbA1c ≥ 8%, chronic renal disease, pancreatitis, chronic pulmonary disease, or psychiatric illness/social situations that would limit compliance with study requirements. Patients must be free of any clinically relevant disease (other than glioma) that would, in the treating investigator's opinion, interfere with the conduct of the study or study evaluations
12. Treatment not initiated within 6 weeks after first biopsy or surgery of glioblastoma
13. Prior enrolment into this study

8.4.2.2 Exclusion Criteria for Expansion Group Arms A and B

A patient will not be eligible for inclusion if any of the following criteria applies during screening or prior to enrolment:

1. Inability to understand and collaborate throughout the study or inability or unwillingness to comply with study requirements
2. Participation in any clinical research study with administration of an investigational drug or therapy within 30 days from screening visit or observation period of competing studies
3. Contra-indication or known hypersensitivity to MRI contrast agents, bevacizumab (Arm A only), olaptesed pegol or polyethylene glycol
4. Planned hypofractionated radiotherapy
5. Cytostatic therapy (chemotherapy) within the past 5 years
6. History of other cancers (except for adequately treated basal or squamous cell skin cancer, in situ cervical cancer, or other cancer from which the patient was disease-free for ≥ 5 years)
7. Secondary malignancy which is currently active
8. Clinically significant or uncontrolled cardiovascular disease, including
    - Myocardial infarction in the previous 12 months
    - Uncontrolled angina
    - Congestive heart failure (New York Heart Association functional classification of ≥2)
    - Diagnosed or suspected congenital long QT syndrome
    - QTc prolongation on an electrocardiogram prior to entry (>470 ms)
    - Uncontrolled hypertension (blood pressure ≥ 160/95 mmHg)
    - Heart rate <50/min on the baseline electrocardiogram
    - History of ventricular arrhythmias of any clinically significant type (such as ventricular tachycardia, ventricular fibrillation or torsades de pointes)
    - Cerebrovascular accident
9. Prior radiotherapy to the head
10. Any other previous or concomitant experimental glioblastoma treatments

11. Placement of Gliadel® wafer, seeds, or ferromagnetic nanoparticles
12. Patients with a history of arterial or venous thrombosis (or any other disease) requiring permanent intake of anticoagulants (Arm A only)
13. Pregnancy or lactation
14. Uncontrolled intercurrent illness including, but not limited to ongoing or active infection, chronic liver disease (e.g., cirrhosis, hepatitis), diabetes mellitus, or subjects with either of the following: fasting blood glucose (FBG defined as fasting for at least 8 hours) ≥ 200 mg/dL (7.0 mmol/L), or HbA1c ≥ 8%, chronic renal disease, pancreatitis, chronic pulmonary disease, auto-immune diseases or psychiatric illness/social situations that would limit compliance with study requirements. Patients must be free of any clinically relevant disease (other than glioma) that would, in the treating investigator's opinion, interfere with the conduct of the study or study evaluations.
15. Prolongation of coagulation factors ≥ 2.5 x ULN (Arm A only)
16. Treatment not initiated within 6 weeks after first biopsy or surgery of glioblastoma
17. Prior enrolment into this study

8.4.2.3 Exclusion Criteria for Expansion Group Arm C

A patient will not be eligible for inclusion if any of the following criteria applies during screening or prior to enrolment:

1. Inability to understand and collaborate throughout the study or inability or unwillingness to comply with study requirements
2. Participation in any clinical research study with administration of an investigational drug or therapy within 30 days from screening visit or observation period of competing studies
3. Contra-indication or known hypersensitivity to MRI contrast agents olaptesed pegol or polyethylene glycol or pembrolizumab (≥ Grade 3)
4. Biopsy-only of GBM with less than 20% of tumor removed
5. Presence of extracranial metastatic or leptomeningeal disease
6. Severe hypersensitivity (≥ Grade 3) to other monoclonal antibodies
7. Receiving immunosuppressive therapy
8. Previous or current treatment with an anti-CTLA-4, anti-PD-1, anti-PD-L1, or anti-PDL2 agent
9. Planned hypofractionated radiotherapy
10. Cytostatic therapy (chemotherapy) within the past 5 years
11. History of other cancers or secondary malignancy which is currently active (except for adequately treated basal or squamous cell skin cancer, in situ cervical cancer, or other cancer from which the patient was disease-free for ≥ 5 years)
12. Clinically significant or uncontrolled cardiovascular disease, including
    - Myocardial infarction in the previous 12 months
    - Uncontrolled angina
    - Congestive heart failure (New York Heart Association functional classification of ≥2)
    - Diagnosed or suspected congenital long QT syndrome

- QTc prolongation on an electrocardiogram prior to entry (>470 ms)
- Uncontrolled hypertension (blood pressure ≥ 160/95 mmHg)
- Heart rate <50/min on the baseline electrocardiogram
- History of ventricular arrhythmias of any clinically significant type (such as ventricular tachycardia, ventricular fibrillation or torsades de pointes)
- Cerebrovascular accident

13. Prior radiotherapy to the head
14. Evidence of acute intracranial / intra-tumoral hemorrhage
15. Any other previous or concomitant experimental glioblastoma treatments
16. Placement of Gliadel® wafer, seeds, or ferromagnetic nanoparticles
17. Pregnancy or lactation
18. Uncontrolled intercurrent illness including, but not limited to ongoing or active infection, chronic liver disease (e.g., cirrhosis, hepatitis), diabetes mellitus, or subjects with either of the following: fasting blood glucose (FBG defined as fasting for at least 8 hours) ≥ 200 mg/dL (7.0 mmol/L), or HbA1c ≥ 8%, chronic renal disease, pancreatitis, chronic pulmonary disease, auto-immune diseases or psychiatric illness/social situations that would limit compliance with study requirements. Patients must be free of any clinically relevant disease (other than glioma) that would, in the treating investigator's opinion, interfere with the conduct of the study or study evaluations.
19. Received a live vaccine within 30 days prior to the first dose of study drug.
20. Known active central nervous system (CNS) metastases and/or carcinomatous meningitis. Previously treated brain metastases may participate provided these remain stable
21. Known history of HIV infection, hepatitis B or hepatitis C infection
22. Active autoimmune disease that has required systemic treatment in past 2 years (i.e. with use of disease modifying agents, corticosteroids or immunosuppressive drugs)
23. History of (non-infectious) pneumonitis / interstitial lung disease that required steroids or current pneumonitis / interstitial lung disease
24. Immunodeficiency diagnosis or receiving chronic systemic steroid therapy (exceeding 10 mg daily of prednisone) or any other form of immunosuppressive therapy
25. High dose of corticosteroids (> 4mg/day of dexamethasone or equivalent for at least 3 consecutive days) within two weeks prior to the first dose of study drug
26. Treatment not initiated within 6 weeks after first biopsy or surgery of glioblastoma
27. Prior enrolment into this study

8.4.3 Patient Withdrawal

In general, patients should not be withdrawn prior to completion of the study.

Patients who withdraw consent to participate must be withdrawn from the study. No justification for such a decision is required. In the event that a patient withdraws from the study, the investigator must be informed immediately. If the patient already received the investigational treatment prior to withdrawal of consent, he/she will be offered to return for a safety follow-up

visit. No data obtained after withdrawal of consent will be recorded on case report forms and will not be evaluated as part of the clinical study.

Patients must be withdrawn if:

- the investigator or DSMB considers it in the best interest of the patients, i.e. any situation in which, in the investigator's or DSMB's opinion, a continuation of the treatment with the study medication would be harmful to the patient's safety and well-being.
- drug related AST, ALT or increased bilirubin of CTCAE grade 3 or 4 (> 5 x ULN for ALT& AST, > 3 x ULN for bilirubin) is reported.
- any other drug-related AE of grade 4 is reported.
- female patients become pregnant. The investigator must immediately notify the sponsor in the event of a confirmed pregnancy in a patient participating in the study.

Further reason for withdrawal could be:

- Serious adverse event (SAE) considered to be related to treatment with the investigational medicinal product
- Failure to comply with the study stipulations, e.g. use of recreational drugs or prohibited medications during the study

In all cases the date, circumstances and any reason provided will be documented on the withdrawal page of the eCRF and in the patient's medical records.

The investigator reserves the right to request the withdrawal of a patient due to protocol deviation(s), administrative or any other valid and ethical reason(s).

Patients withdrawn from the study will be informed about the reasons for their withdrawal.

In any case, if possible, a final study examination should be performed on patients who discontinue prematurely. This examination should include all procedures performed at the end of treatment visit. All ongoing AEs/SAEs of withdrawn patients have to be followed up until no more signs and symptoms are verifiable or the patient is in stable condition.

8.4.4 Replacement of Patients

Based on the drop-out rate during the study, drop-outs might be replaced. Up to two drop-outs per cohort may be replaced. Only those subjects will be replaced which have not withdrawn due to adverse drug reactions or adverse events based on study procedures.

8.4.5 Restrictions during the Study

Alcohol should not be consumed during olaptesed pegol application.

No intake of drugs of abuse during the entire course of the study.

No participation in another clinical study within 30 days prior to baseline and/or during the entire course of the study.

8.4.5.1 Diet

Patients should maintain a normal diet unless modifications are required to manage an AE such as diarrhea, nausea or vomiting.

8.4.5.2 Contraception

Olaptesed pegol may have adverse effects on a fetus in utero. Furthermore, it is not known if olaptesed pegol has transient adverse effects on the composition of sperm.

Women should be informed that taking the study medication may involve unknown risks to the fetus (unborn baby) if pregnancy were to occur during the study. In order to participate in the study women of childbearing potential as well as female partners of male participants must adhere to the contraception requirement (described below) from the day of study medication initiation (or 14 days prior to the initiation of study medication for oral contraception) throughout the study period up to 120 days (180 days for Arm A, 120 days for Arm C) after the last dose of study therapy. If there is any question that a woman of childbearing potential will not reliably comply with the requirements for contraception, that woman should not be entered into the study.

Medically acceptable methods of birth control are methods with a low failure rate of less than 1% per year; e.g. combined (estrogen and progestogen containing) hormonal contraception associated with inhibition of ovulation (oral, intravaginal, transdermal), progestogen-only hormonal contraception associated with inhibition of ovulation (oral, injectable, implantable), intrauterine device (IUD), intrauterine hormone-releasing system (IUS), bilateral tubal occlusion, vasectomized partner (provided that partner is the sole sexual partner of the study participant and that the vasectomized partner has received medical assessment of the surgical success), sexual abstinence during and for 120 days following last dose of drug.

All women, including those with tubal ligation, are considered to be of childbearing potential unless they have been postmenopausal for at least 2 years. Hysterectomized women are considered surgically sterile and are not required to use any contraception.

8.4.5.3 Use in Pregnancy

Women should be counseled to contact the investigator or his staff immediately if pregnancy is suspected. If a patient inadvertently becomes pregnant while on treatment with olaptesed pegol, the patient will immediately be removed from the study. The site will contact the patient at least monthly and document the patient's status until the pregnancy has been completed or terminated. The outcome of the pregnancy will be reported to the Sponsor without delay and within 24 hours if the outcome is a serious adverse experience (e.g., death, abortion, congenital anomaly, or other disabling or life-threatening complication to the mother or newborn). The investigator will make every effort to obtain permission to follow the outcome of the pregnancy and report the condition of the fetus or newborn to the Sponsor.

8.4.5.4 Use in Nursing Women

It is unknown whether olaptesed pegol is excreted in human milk. Since many drugs are excreted in human milk, and because of the potential for serious adverse reactions in the nursing infant, patients who are breast-feeding are not eligible for enrollment.

8.4.6 Termination of the Study

The Sponsor reserves the right to prematurely discontinue the study at any time. If the study is terminated or suspended, the sponsor and/or its designees will promptly inform the investigators/institutions and regulatory authorities. The IRB/EC should promptly be informed and provided the reasons(s) for the termination or suspension by the investigator/sponsor, as specified by the applicable regulatory requirement(s).

Reasons for terminating the study may include the following:

- the incidence or severity of AEs in this study indicates a potential health hazard to study patients.
- patient enrolment is unsatisfactory.
- data recording is inaccurate or incomplete.

The Sponsor may terminate the study at a study site when the investigator fails to comply with relevant regulations or insufficiently adheres to protocol requirements or recruitment remains insufficient.

8.5 Treatments

8.5.1 Identity of Investigational Product(s)

8.5.1.1 Olaptesed Pegol

Dosage form:	Solution for injection
Active ingredient:	Olaptesed Pegol, 100 mg / 6.8 mL
[redacted]	[redacted]
[redacted]	[redacted]
Route of administration:	Continuous infusion
Total weekly dose:	200, 400 or 600 mg
Packaging:	glass vials

The investigational product was produced and released according to current European Good Manufacturing Practice regulations. [redacted].

8.5.1.2 MVASI® (Bevacizumab)

Dosage form:	Solution for infusion
Active ingredient:	Bevacizumab, 100 mg / 4 ml or 400 mg / 16 mL
Inactive ingredients:	Trehalose dihydrate, sodium phosphate, polysorbate 20, water for injections

Presentation:	Clear to slightly opalescent, colorless to slightly yellow liquid
Route of administration:	Intravenous infusion
Dosing:	10 mg / kg body weight every 2 weeks
Packaging:	Glass vials

The investigational product was produced and released according to current European Good Manufacturing Practice regulations. Study medication has to be stored between 2-8°C and protected from light.

8.5.1.3 KEYTRUDA® (Pembrolizumab)

Dosage form:	Solution for infusion
Active ingredient:	Pembrolizumab, 100 mg / 4 mL
Inactive ingredients:	L-histidine, L-histidine hydrochloride monohydrate, sucrose, polysorbate 80, water for injections.
Presentation:	Clear to slightly opalescent, colorless to slightly yellow solution, pH 5.2 – 5.8.
Route of administration:	Intravenous infusion
Dosing:	200 mg every 3 weeks
Packaging:	Glass vials

The investigational product was produced and released according to current European Good Manufacturing Practice regulations. Study medication has to be stored between 2-8°C and protected from light.

8.5.2 Packaging and Labelling

8.5.2.1 Olaptesed Pegol

Olaptesed pegol will be supplied by the Sponsor including a certificate of compliance issued by a European Qualified Person (QP).

[redacted]

[redacted]

The study medication will be labelled according to local requirements.

Olaptesed pegol will be shipped under temperature control and monitoring. The receiving site has to confirm the receipt and the condition of the delivery in writing on provided forms. Should the temperature-monitoring device indicate that the specified shipment conditions were not maintained during the transport, olaptesed pegol must be quarantined such that it cannot be used unintentionally, and the Sponsor must be contacted immediately. [redacted].

8.5.2.2 MVASI® (Bevacizumab)

Bevacizumab is a recombinant humanized monoclonal antibody produced by DNA technology in Chinese Hamster Ovary cells. Bevacizumab is a concentrate for solution for infusion filled as either 4 mL solution in a vial (Type I glass) with a stopper (butyl rubber) containing 100 mg of bevacizumab or as 16 mL solution in a vial (Type I glass) with a stopper (butyl rubber) containing 400 mg of bevacizumab.

MVASI will be supplied by the Sponsor. Supply will be performed by Haupt Pharma Wuelfing, Gronau, Germany and will be in compliance with EU and local regulations where applicable.

MVASI will be shipped under temperature control and monitoring. The receiving site has to confirm the receipt and the condition of the delivery in writing on provided forms. Should the temperature-monitoring device indicate that the specified shipment conditions were not maintained during the transport, MVASI must be quarantined such that it cannot be used unintentionally, and the Sponsor must be contacted immediately. Upon receipt MVASI will be stored refrigerated (2-8°C) and protected from light in a place with restricted and controlled access. MVASI may not be frozen. MVASI may not be shaken.

8.5.2.3 KEYTRUDA® (Pembrolizumab)

Pembrolizumab is a concentrate for solution for infusion filled as 4 mL solution in a 10 mL Type I clear glass vial, with a coated grey chlorobutyl or bromobutyl stopper and an aluminum seal with a dark blue colored flip-off cap, containing 100 mg pembrolizumab.

KEYTRUDA will be supplied by the Sponsor. Supply will be performed by Medizone Germany GmbH, Oberhaching, Germany and will be in compliance with EU and local regulations where applicable.

KEYTRUDA will be shipped under temperature control and monitoring. The receiving site has to confirm the receipt and the condition of the delivery in writing on provided forms. Should the temperature-monitoring device indicate that the specified shipment conditions were not maintained during the transport, KEYTRUDA must be quarantined such that it cannot be used unintentionally, and the Sponsor must be contacted immediately. Upon receipt KEYTRUDA will be stored refrigerated (2-8°C) and protected from light in a place with restricted and controlled access. KEYTRUDA may not be frozen. KEYTRUDA may not be shaken.

8.5.3 Preparation

8.5.3.1 Olaptesed Pegol

Details on preparation and administration of olaptesed pegol are provided in the IMP Handling Instructions.

[redacted].

8.5.3.2 MVASI® (Bevacizumab)

MVASI will be prepared according to clinical practice guidelines in accordance with the current approved SmPC. The necessary amount of bevacizumab will be withdrawn and diluted to the required administration volume with sodium chloride 9 mg/mL (0.9%) solution for injection. The concentration of the final bevacizumab solution should be kept within the range of 1.4 mg/mL to 16.5 mg/mL. In the majority of the occasions the necessary amount of bevacizumab can be diluted with 0.9 % sodium chloride solution for injection to a total volume of 100 mL.

The dilution should be inspected visually for particulate matter and discoloration prior to administration. Bevacizumab infusions should not be administered or mixed with glucose solutions. This medicinal product must not be mixed with other medicinal products except those mentioned above.

MVASI is for single use only, as the product contains no preservatives.

8.5.3.3 KEYTRUDA® (Pembrolizumab)

KEYTRUDA will be prepared according to clinical practice guidelines in accordance with the current approved SmPC. The vial should be inspected visually for particulate matter and discoloration prior to administration. The concentrate is a clear to slightly opalescent, colorless to slightly yellow solution. The required volume of pembrolizumab, up to 4 mL (100 mg) of concentrate, will be withdrawn and transferred into an intravenous bag containing sodium chloride 9 mg/mL (0.9%) or glucose 50 mg/mL (5%) to prepare a diluted solution with a final concentration ranging from 1 to 10 mg/mL. Each vial contains an excess fill of 0.25 mL (total content per vial 4.25 mL) to ensure the recovery of 4 mL of concentrate.

KEYTRUDA infusions should not be co-administer other medicinal products through the same infusion line.

KEYTRUDA is for single use only. Discard any unused portion left in the vial.

8.5.4 Complaints

Pharmaceutical technical complaints associated with the investigational products like particles visible or solution not clear, must be reported to the sponsor immediately.

8.5.5 Olaptesed Pegol Treatment Doses and Administration

The dosing regimen is planned as follows. Treatments will be administered after all procedures and assessments have been completed as detailed in the Flow Chart (Table 2-1). Unless otherwise noted all treatments will be on Day 1 of each week.

After start of the continuous administration of olaptesed pegol on day 1, patients should stay at the site under the supervision of the site staff for at least 1 h.

During Weeks 1 to 6, radiotherapy according to standard of care will be administered as external-beam radiotherapy of a cumulative dose of 60 Gy in daily fractions of 2 Gy (5 per week, Mo through Fr). Elderly patients may receive a hypofractionated radiotherapy scheme (40.05 Gy given in 15 fractions of 2.67 Gy).

Olaptesed pegol will be continuously administered i.v. using an active pump at a rate of 200, 400 or 600 mg/week for a total of 26 weeks beginning with initiation of radiotherapy.

On the days of change of medication cassette that coincide with the administration of bevacizumab, the infusion with olaptesed pegol will be stopped before administration of bevacizumab. Infusion will be re-started with the new cassette post administration of bevacizumab.

On the days of change of medication cassette that coincide with the administration of pembrolizumab, the infusion with olaptesed pegol will be stopped before administration of pembrolizumab. Infusion will be re-started with the new cassette 30-60 minutes post administration of pembrolizumab.

In case the patient experiences problems with the port system (e.g. reddening, rash) that prevent administration of olaptesed pegol as continuous infusion using the pump, olaptesed pegol should be administered i.v. twice weekly (Day 1 and Day 4) at a fixed dose of 100, 200 or 300 mg respectively. Patients should be placed on continuous infusion as soon as the port issue resolved.

Patients in the expansion Arm A will receive concomitant bevacizumab.

Bevacizumab will be administered according to clinical practice guidelines at doses of 10 mg/kg every two weeks (7,8). The initial dose should be delivered over 90 minutes as an i.v. infusion. If the first infusion is well tolerated, the second infusion may be administered over 60 minutes. If the 60-minute infusion is well tolerated, all subsequent infusions may be administered over 30 minutes. It should not be administered as an intravenous push or bolus.

Patients in the expansion Arm C will receive concomitant pembrolizumab.

Pembrolizumab will be administered according to clinical practice guidelines at doses of 200 mg every 3 weeks. It should be delivered over 30 minutes as an i.v. infusion. Pembrolizumab must not be administered as an intravenous push or bolus injection.

For use in combination, see the SmPC for the concomitant therapies. When administering pembrolizumab as part of a combination therapy, pembrolizumab should be administered first.

Treatment with olaptesed pegol will be discontinued at the discretion of the investigator in case of intolerable toxicity. Treatment with olaptesed pegol alone or in combination with either

bevacizumab or pembrolizumab may be continued beyond 26 weeks if the patient has clear clinical benefit according to the investigator.

Treatment interruption is not permitted for more than 2 consecutive days and/or 7 days in total. Longer interruption of treatment with olaptesed pegol will be considered as major protocol violation.

8.5.5.1 Dose Escalation Cohorts

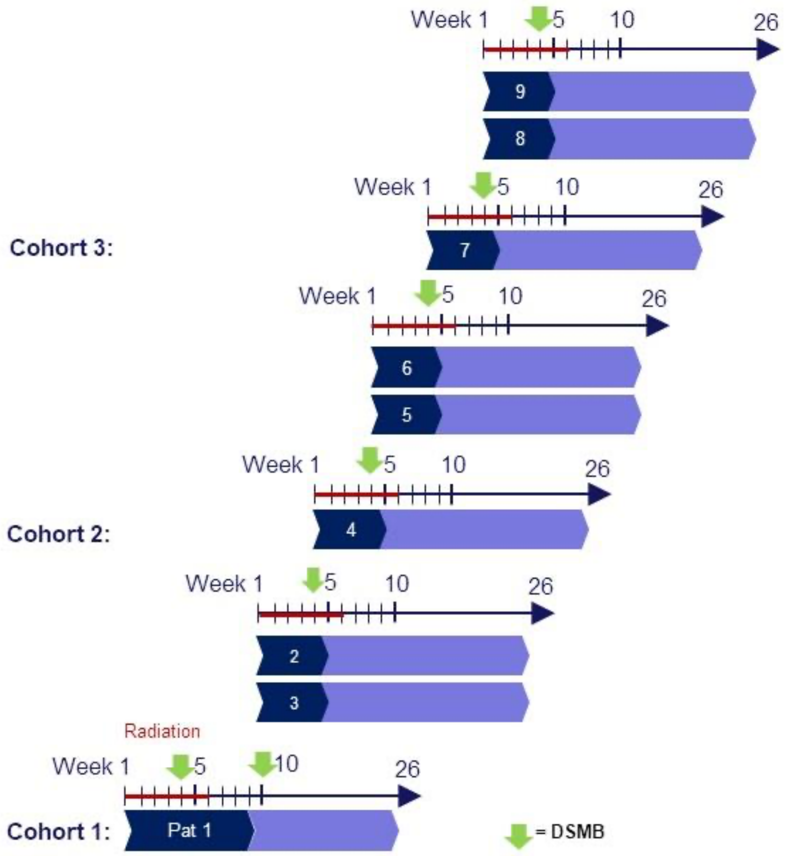

Figure 8-2: Dose escalation in three cohorts

Patients will be enrolled in three successional cohorts consisting of three patients each. Patients of Cohort 1 will be treated with a weekly dose of 200 mg, of Cohort 2 with a weekly dose of 400 mg and of Cohort 3 with a weekly dose of 600 mg olaptesed pegol.

After 4 weeks of treatment with 200 mg per week olaptesed pegol plus radiotherapy (including the radiotherapy phase) of the first patient of Cohort 1, the DSMB will review all DLTs, AEs, and relevant laboratory values. Until the next review after 10 weeks of treatment the DSMB will be informed continuously about all DLTs and SAEs. After 10 weeks of treatment the DSMB will review of all DLTs, AEs, and relevant laboratory values including olaptesed pegol plasma concentrations prior to enrolment of the next two patients of this cohort. Evaluation of all DLTs, AEs, and relevant laboratory values including olaptesed pegol plasma concentrations will be performed prior to enrolling patients in Cohort 2 after patients 2 and 3 received at least 4 weeks of treatment.

After the first patient of Cohort 2 received 4 weeks of treatment with weekly 400 mg olaptesed pegol in combination with radiotherapy, the DSMB will review all DLTs, AEs, and relevant laboratory values including olaptesed pegol plasma concentrations prior to enrolment of the next two patients of this cohort. The same evaluation will be performed prior to enrolling patients in Cohort 3 after patients 5 and 6 received at least 4 weeks of treatment.

After the first patient of Cohort 3 received 4 weeks of treatment with weekly 600 mg olaptesed pegol in combination with radiotherapy, the DSMB will review all DLTs, AEs, and relevant laboratory values including olaptesed pegol plasma concentrations prior to enrolment of the last two patients of this cohort. The same evaluation will be performed after patients 8 and 9 received at least 4 weeks of treatment.

The DSMB will also meet on an ad hoc basis if 2 or more DLTs are observed in any cohort.

If none of the three patients in any Cohort experiences a DLT, another three patients will be treated at the next higher dose level. However, if one of the three patients in a Cohort experiences a DLT, three more patients will be treated at the same dose level. The dose escalation continues until at least two patients among a cohort of three to six patients experience DLT (i.e., ≥ 33% of patients with a dose-limiting toxicity at that dose level), but the dose will not be escalated beyond 600 mg/week. The recommended dose for phase II trials is conventionally defined as the dose level just below this toxic dose level (39), or 600 mg/week if this dose level is not toxic.

Furthermore, the DSMB may recommend decreasing the weekly infused dose based on the results from safety evaluations. The DSMB will also meet on an ad hoc basis if 2 or more DLTs are observed in any arm.

8.5.5.2 Expansion Group – Arm A and Arm C

After the first patient in Arm A has received 4 weeks of treatment with weekly 600 mg olaptesed pegol in combination with radiotherapy and bevacizumab, the DSMB will review all DLTs, AEs, and relevant laboratory values including olaptesed pegol plasma concentrations.

The same procedure will apply to Arm C.

8.5.6 Radiotherapy

Prior to initiation of external-beam radiotherapy (EBRT), the craniotomy wound must be adequately healed, and all sutures, stitches or drains must be removed. If any condition (e.g., wound healing difficulties, infections) does not permit the initiation of RT within this timeframe, a further postponement of one week is allowed. If RT can still not be applied after this extension period (6 weeks after surgery), the patient is withdrawn from the study.

EBRT requires LINACs maximum with photon energies ≥ 4 MV capable of delivering static or dynamic intensity modulation. Intensity-Modulated Radiotherapy (IMRT) is allowed. Electron or particle irradiation is not allowed. Image-guided radiotherapy (IGRT), e.g. with cone-beam verification, is favored. Treatment verification and documentation should be carried out based on institutional policy.

For treatment planning, a T1-Gd-MRI MRI sequence has to be co-registered on the planning-CT acquired in treatment position. The accuracy of co-registration has to be confirmed by the radiation oncologist.

EBRT may be interrupted for a maximum period of seven consecutive days. The reason for interruption and the duration must be documented in the eCRF. Any longer treatment pause is considered as unacceptable protocol violation.

8.5.6.1 Target Volume Definition and Dosing of Conventional Fractionation

Target volume definition must follow RTOG (two target volumes with one including the T2/FLAIR) or EORTC (one target volume without the T2/FLAIR) recommendations, whereas each center has to commit and adhere to one of both definitions.

The following definitions apply for sites that chose RTOG recommendations: the GTV1 resembles the edema in T2-FLAIR sequences and must also cover the complete resection cavity and any contrast-enhancing lesion in the T1-post contrast sequences. If no surrounding edema is visible, the GTV1 includes the contrast-enhancing lesion (and the surgical resection cavity) plus a 2.5 cm margin. The *clinical target volume 1* (CTV1) is then defined as the GTV1 plus a 2 cm margin. The CTV1 may be reduced around natural margins for tumors (e.g., skull, falx, etc.). The *planning target volume 1* (PTV1) then resembles the CTV1 plus an additional margin of 2-5 mm. All (T1-)contrast-enhancing lesions/abnormalities (which must include the cavity margins) will be defined as boost volume (GTV2). The boost clinical target volume (CTV2) will be the GTV2 plus a margin of 2 cm. The CTV2 may also be reduced around natural margins for tumors (e.g., skull, falx, etc.). The *boost planning target volume* (PTV2) resembles the CTV2 plus an additional margin of 2-5 mm.

The following definitions apply for sites that chose EORTC recommendations: The GTV includes all contrast-enhancing lesion in a T1-weighted MRI and the resection cavity. The CTV is then generated by extending the GTV by a 2 cm margin. The PTV then resembles the CTV plus an additional margin of 2-5 mm. EBRT will then be applied up to a total dose of 60 Gy to the PTV.

EBRT will be applied up to a total dose of 46 Gy to the PTV1 and a sequential boost of 14 Gy (60 Gy total dose) to the PTV2. EBRT is given in daily doses of 2 Gy, 5 days per week (6 weeks total duration time). At least 95% of each PTV shall receive 100% of the prescribed dose and the minimum dose to both PTVs should be not less than 90% of the planned doses. The maximum dose to the PTV (EORTC) or PTV2 (EORTC) should not exceed 68 Gy.

8.5.6.2 Target Volume Definition and Dosing for Hypofractionation (Elderly Patients)

Hypofractionated EBRT will be applied as per the NCIC CTG CE.6 protocol with only one volume with no boost/cone-down. Thus, the GTV will be the entire contrast-enhancing tumor (T1-MRI). If the tumor has been partially resected, the GTV will be the surgical cavity plus all contrast-enhancing residual tumor. The CTV is defined as the GTV plus a 1.5 cm margin, with no expansion outside of any natural barriers (see above, e.g. skull and tentorium). The PTV is then defined as the CTV plus 2-5 mm in all directions.

Patients will receive a total dose of 40.05 Gy to the PTV, delivered in 15 daily fractions over 3 weeks. Each fraction of 2.67 Gy will be given daily, 5 days per week. At least 95% of the PTV

shall receive 100% of the prescribed dose and the minimum dose to the PTV should not be less than 90% of the planned dose. The maximum dose to the PTV should not exceed 42 Gy.

8.5.6.3 Organs at Risk

Organs at risk (OARs) are the optic nerves, optic chiasm, eye globes (including lenses and retinas), and the brain stem. The following OARs must be defined along with a planning risk volume (PRV) for each OAR (OAR + 3 mm): optic nerve, chiasm, brain stem. If an OAR is in proximity to any PTV, all dose to the PTV^{OAR} must be as close as possible to the planned total dose (46/60 Gy or 40.05 Gy) while not exceeding the OAR dose limit. If an OAR is overlapping the PTV a second PTV (PTVOAR) may be defined as PTV – (OAR + 3 mm margin). All dose to the PTVOAR must be as close as possible to the prescribed total dose (46/60 Gy or 40.05 Gy) while not exceeding the OAR dose limit. The maximum point doses permissible to critical structures are: PRV lenses 7 Gy, PRV retinae 50 Gy, PRV optic nerves 55 Gy, PRV chiasm 56 Gy, PRV brain stem 60 Gy.

8.5.7 Dose Limiting Toxicities

Dose limiting toxicities (DLT) according to the Common Terminology Criteria for Adverse Events (CTCAE v.5.0) are defined as any grade 3-4 non-hematological toxicities (excluding grade 3 vomiting and/or nausea, if encountered without adequate and optimal prophylactic therapy), at any dose-level, assessed by the Investigator and/or the Sponsor as related to olaptesed pegol.

The DSMB will evaluate DLTs and the safety of study participants. The DSMB will meet on an ad hoc basis if 2 or more DLTs are observed. DLTs may result in a down titration of olaptesed pegol.

DLTs will be observed throughout the first four weeks of combined radiotherapy with protracted infusion of olaptesed pegol. Radiotherapy will be generally administered as EBRT over six weeks to a cumulative dose of 60 Gy in daily fractions of 2 Gy (5 per week, Mo through Fr), and the four-week observation period will also apply for patients aged 65 years and older which may alternatively receive hypofractionated radiotherapy of 40.05 Gy in 15 fractions.

8.5.8 Dose Modification

8.5.8.1 Olaptesed Pegol

Olaptesed pegol must be withheld for drug-related toxicities and severe or life-threatening AEs as per Table 8-4 below. In case of restarting treatment with olaptesed pegol after holding for toxicities as described in Table 8-4, patients will initially receive 75% of the last weekly dose. If the respective toxicity doesn’t recur after 4 weeks, the dose will be ramped up to full last weekly dose.

Table 8-4: Olaptesed pegol Dose Modification Guidelines for drug-related Adverse Events

Toxicity	Hold Treatment for Grade	Timing for Restarting Treatment	Treatment Discontinuation
AST, ALT, or Increased Bilirubin	2	Toxicity resolves to Grade 0-1	Toxicity does not resolve within 12 weeks of last dose
	3-4	Permanently discontinue	Permanently discontinue
All other drug-related toxicity*	3 or Severe	Toxicity resolves to Grade 0-1	Toxicity does not resolve within 12 weeks of last dose
	4	Permanently discontinue	Permanently discontinue

Note: Permanently discontinue for any severe or Grade 3 drug-related AE in case of recurrence.
* Patients with intolerable or persistent Grade 2 drug-related AE may hold study medication at the discretion of the investigator. Permanently discontinue study drug for such AEs that do not recover to Grade 0-1 within 12 weeks of the last dose.

8.5.8.2 MVASI® (Bevacizumab)

No dose reductions of MVASI for adverse reactions are recommended. If indicated, treatment with MVASI should either be permanently discontinued or temporarily suspended. Table 8-5 describes dose modifications for specific adverse reactions.

Table 8-5: MVASI Dosage Modifications for Adverse Reactions

Adverse Reaction	Severity	Dose Modification
Gastrointestinal Perforations and Fistulae	• Gastrointestinal perforation, any grade<br>• Tracheoesophageal fistula, any grade<br>• Fistula, Grade 4<br>• Fistula formation involving any internal organ	Discontinue
Wound Healing Complications[1]	• Wound healing complications requiring medical intervention<br>• Necrotizing fasciitis	Discontinue
Hypertension	• Hypertensive crisis<br>• Hypertensive encephalopathy	Discontinue
	• Hypertension, severe	Withhold if not controlled with medical management; resume once controlled
Posterior Reversible Encephalopathy Syndrome (PRES)	• Any	Discontinue
Proteinuria	• Nephrotic syndrome	Discontinue
	• Proteinuria greater than or equal to 2 grams per 24 hours in absence of nephrotic syndrome	Withhold until proteinuria less than 2 grams per 24h

Adverse Reaction	Severity	Dose Modification
Thromboembolic Events	• Arterial thromboembolism, severe	Discontinue
	• Venous thromboembolism, Grade 4	Discontinue
Hemorrhage[2]	• Grade 3 or 4	Discontinue
	• Recent history of hemoptysis of > 2.5 mL of red blood	Withhold
Infusion-Related Reactions	• Severe	Discontinue
	• Clinically significant	Interrupt infusion; resume at a decreased rate of infusion after symptoms resolve
	• Mild, clinically insignificant	Decrease infusion rate

1: Treatment should not be initiated for at least 28 days following major surgery or until the surgical wound is fully healed. In patients who experienced wound healing complications during treatment, treatment should be withheld until the wound is fully healed. Treatment should be withheld for elective surgery.
2: Patients should be monitored for signs and symptoms of CNS bleeding, and MVASI treatment discontinued in cases of intracranial bleeding.

8.5.8.3 KEYTRUDA® (Pembrolizumab)

No dose reductions of KEYTRUDA are recommended. KEYTRUDA should be withheld or discontinued to manage adverse reactions as described in Table 8-6.

The safety of re-initiating pembrolizumab therapy in patients previously experiencing immune-related myocarditis is not known. KEYTRUDA, as monotherapy or as combination therapy, should be permanently discontinued for Grade 4 or recurrent Grade 3 immune-related adverse reactions, unless otherwise specified in Table 8-6. For Grade 4 hematological toxicity, only in patients with cHL, KEYTRUDA should be withheld until adverse reactions recover to Grades 0-1.

Table 8-6: Recommended treatment modifications for KEYTRUDA

Immune-related adverse reactions	Severity	Treatment modification
Pneumonitis	Grade 2	Withhold until adverse reactions recover to Grades 0-1*
	Grades 3 or 4, or recurrent Grade 2	Permanently discontinue
Colitis	Grades 2 or 3	Withhold until adverse reactions recover to Grades 0-1*
	Grade 4 or recurrent Grade 3	Permanently discontinue
Nephritis	Grade 2 with creatinine > 1.5 to ≤ 3 times upper limit of normal (ULN)	Withhold until adverse reactions recover to Grades 0-1*
	Grade ≥ 3 with creatinine > 3 times ULN	Permanently discontinue

Immune-related adverse reactions	Severity	Treatment modification
Endocrinopathies	Grade 2 adrenal insufficiency and hypophysitis	Withhold treatment until controlled by hormone replacement
	Grades 3 or 4 adrenal insufficiency or symptomatic hypophysitis<br><br>Type 1 diabetes associated with Grade ≥ 3 hyperglycemia (glucose > 250 mg/dL or > 13.9 mmol/L) or associated with ketoacidosis<br><br>Hyperthyroidism Grade ≥ 3	Withhold until adverse reactions recover to Grades 0-1*<br><br>For patients with Grade 3 or Grade 4 endocrinopathies that improved to Grade 2 or lower and are controlled with hormone replacement, if indicated, continuation of pembrolizumab may be considered after corticosteroid taper, if needed. Otherwise, treatment should be discontinued.
	Hypothyroidism	Hypothyroidism may be managed with replacement therapy without treatment interruption.
Hepatitis	Grade 2 with AST or ALT > 3 to 5 times ULN or<br>total bilirubin > 1.5 to 3 times ULN	Withhold until adverse reactions recover to Grades 0-1*
	Grade ≥ 3 with AST or ALT > 5 times ULN or total bilirubin > 3 times ULN	Permanently discontinue
	In case of liver metastasis with baseline Grade 2 elevation of AST or ALT, hepatitis with AST or ALT increases ≥ 50% and lasts ≥ 1 week	Permanently discontinue
Skin reactions	Grade 3 or suspected Stevens-Johnson syndrome (SJS) or toxic epidermal necrolysis (TEN)	Withhold until adverse reactions recover to Grades 0-1*
	Grade 4 or confirmed SJS or TEN	Permanently discontinue
Other immune-related adverse reactions	Based on severity and type of reaction (Grade 2 or Grade 3)	Withhold until adverse reactions recover to Grades 0-1*
	Grades 3 or 4 myocarditis<br>Grades-3 or 4 encephalitis<br>Grades-3 or 4 Guillain-Barré syndrome	Permanently discontinue
	Grade 4 or recurrent Grade 3	Permanently discontinue
Infusion-related reactions	Grades 3 or 4	Permanently discontinue

Note: toxicity grades are in accordance NCI-CTCAE v.4.0.
* If treatment-related toxicity does not resolve to Grades 0-1 within 12 weeks after last dose of KEYTRUDA, or if corticosteroid dosing cannot be reduced to ≤ 10 mg prednisone or equivalent per day within 12 weeks, KEYTRUDA should be permanently discontinued.

8.5.9 Prior and Concomitant Therapy

All concomitant medications received within 30 days before the first dose of study treatment and 30 days after the last dose of study treatment must be documented in the corresponding section of the eCRF. Other relevant previous medication as judged by the investigator should also be documented in the eCRF. Concomitant medications administered after 30 days after the last dose of study treatment should be recorded for SAEs.

All treatments being taken by the patients at entry into the study and all treatments given in addition to the study treatment during the study are regarded as concomitant treatments and must be documented in the eCRF. Any change in the concomitant medication will be documented.

Concomitant medications should be kept to a minimum during the study. However, if these are considered necessary for the patient's welfare and are unlikely to interfere with the study objectives, they may be allowed at the discretion of the investigator.

8.5.9.1 Unacceptable Previous Therapy

Any previous radiotherapy to the head is not allowed.

Any previous or concomitant experimental glioblastoma treatment is not allowed.

Any placement of Gliadel® wafer, seeds, or ferromagnetic nanoparticles is not allowed.

Chemotherapies are not allowed 5 years prior to the first dose of study drug and during study treatment.

Previous or current treatment with an anti-CTLA-4, anti-PD-1, anti-PD-L1, or anti-PDL2 agent (Arm C only).

Live vaccine within 30 days prior to the first dose of study drug (Arm C only).

Chronic systemic steroid therapy (exceeding 10 mg daily of prednisone) or any other form of immunosuppressive therapy (Arm C only).

High dose of corticosteroids (> 4mg/day of dexamethasone or equivalent for at least 3 consecutive days) within two weeks prior to the first dose of study drug (Arm C only).

Systemic treatment of an active autoimmune disease in past 2 years, i.e. use of disease modifying agents, corticosteroids or immunosuppressive drugs (Arm C only).

8.5.10 Rescue Medication

No rescue medication is foreseen in this study.

8.5.11 Treatment after End of the Study

After the planned treatment period of the study has ended, no further olaptesed pegol will be provided for patients.

8.5.12 Drug Accountability and Treatment Compliance

The IMP must not be used outside the context of this study protocol. The investigator or authorized staff is obliged to document the receipt, dispensation, and return of all IMP received during this study in accordance with the protocol and any applicable laws and regulations. Records on receipt, use, return, loss, or other disposition of IMP must be maintained by the investigator. The investigator or, if applicable, pharmacist must sign the respective forms used. Records on IMP delivery to the site, the inventory at the site, the use by each patient, and the return to the manufacturer must be maintained by the investigator and/or a pharmacist or another appropriately trained individual at the investigational site. These records will include dates, quantities, batch numbers, and the unique code numbers assigned to the IMP and patients. The investigators must maintain records documenting that the patients were provided with the doses specified in the protocol. Furthermore, they should reconcile all IMP received from the manufacturer. It is the responsibility of the investigator to give reasons for any discrepancies in IMP accountability. Forms will be provided to enhance drug accountability. Following authorization by the sponsor and/or study manager, all unused products will be collected by the monitor or designee and returned to the Sponsor or manufacturer for destruction. IMP will be administered in the clinical unit under the supervision of a physician trained and skilled in emergency and life-saving procedures. The investigator or designee will record the time and dose of all administrations of IMP. Any reasons for non-compliance must be documented including missed visits or interruptions in administration.

8.5.13 Method of Assigning Patients to Treatment Groups

Each patient is uniquely identified by his/her patient number. Upon signature of informed consent each patient is assigned a five-digit patient number by the EDC system via the investigator, which is composed of:

Digits 1 and 2:	Study site (01, 02, 03, etc.)
Digits 3, 4 and 5:	Individual patient number within the site (consecutively in the order of enrollment within the center: 001, 002, etc.)

At each site, the first patient is assigned to patient number 001, and subsequent patients are assigned to consecutive numbers (e.g. the second patient is assigned to patient number 002; the third patient is assigned to patient number 003). Once assigned to a patient this unique identifier is not to be reused. Each olaptesed pegol medication package contains 10 vials and is labeled with a 4-digit box number. Vial numbers consumed at each visit will be recorded on the respective IMP preparation forms used.

8.5.14 Blinding

This is an open-label study.

9. Study Procedures and Assessments

9.1 Study Visits

An overview/flow chart on study conduct is provided in the Section 2.

9.1.1 Informed Consent Visit / Screening

The patient will be fully informed of all study procedures and implications both verbally and in writing via use of an informed consent form. See Section 14.4 for more information regarding the patient consent process. Once a patient signed the informed consent form, a patient number (identifier) is to be assigned.

Within 28 days prior to the administration of the first dose of study medication, the following procedures and assessments must be completed, and all inclusion and exclusion criteria must be met.

9.1.1.1 Day -28 to Day -8

The following observations/procedures are to be performed and checked:

- Obtain informed consent
- Check of inclusion and exclusion criteria
- Demographic data (including height and weight)
- Medical history
- Previous and concomitant medication
- Adverse Events
- Physical examination including neurological examination (NANO scale)
- Vital signs
- 12-lead ECG
- Quality of life assessments: EORTC-QLQ C30, BN 20
- Blood sampling for clinical chemistry, hematology, coagulation, immunogenicity and biomarkers
- Serum pregnancy test (females of child-bearing potential)

9.1.1.2 Day -7 to Day -1

The investigator will review all information obtained from the screening procedures. Screening failures; i.e. screened patients not in compliance with all criteria; are to be excluded and the reason will be recorded in the patient notes. Patients who fulfill all the inclusion criteria and none of the exclusion criteria will be eligible to participate in the study. Information of patient's study participation can be provided to the patient's general practitioner upon request.

Once eligibility is confirmed, the following assessments will be performed:

- Response assessments:
  - Imaging (brain MRI including multiparametric sequences)
  - Define baseline for RANO subsequent assessments
- Adverse events since last visit
- Concomitant medication

Thereafter the subcutaneous port will be implanted.

9.1.2 Treatment Period: Week 1

Each patient will complete the following procedures and assessments:

- Adverse events since last visit
- Concomitant medication
- 12-lead ECG
- Vital signs
- Blood sampling
  - Clinical chemistry (Day 1)
  - Hematology (Day 1)
  - Pharmacokinetics and immunogenicity (Days 1 (pre-dose), 2 and 5)
  - Biomarkers (Days 1 (pre-dose) and 2)
  - Pharmacogenetic (pre-dose Day 1)
- Pregnancy test (urine or blood)

Dosing/treatment:

- Day 1: Expansion Arm A: MVASI
- Day 1: Expansion Arm C: KEYTRUDA
- Day 1: connection of the olaptesed pegol infusion pump to port-a-cat
- Day 2: check of infusion pump and selection of flow rate program for Week 1 Days 2-7
- Radiation therapy on Days 2, 3, 4, and 5

9.1.3 Treatment Period: Week 2 - 6

On Day 1 of each week each patient will complete the following procedures and assessments during the 5-week period:

- Adverse events since last visit
- Concomitant medication
- 12-lead ECG (Week 5)
- Vital signs

- Blood sampling
  - Clinical chemistry
  - Hematology
  - Pharmacokinetics and immunogenicity (Weeks 2, 4 and 6)
  - Biomarkers (Weeks 2, 4 and 6)
- Pregnancy test (urine or blood, Week 5)
- Vital signs (at last day of RT only)
- Neurological examination (NANO scale) (at last day of RT only)

Dosing/treatment:

- Week 2 Day 1: selection of flow rate program for the remaining treatment period
- Week 3 and 5, Day 1: Expansion Arm A: MVASI
- Week 4 Day 1: Expansion Arm C: KEYTRUDA
- Week 2 - 6, Day 1: weekly change of infusion pump container
- Radiation therapy on Days 1, 2, 3, 4, and 5

9.1.4 Treatment Period: Week 7, 8, 10, 11, 12, 13, 14, 15, 16, 17, 19, 20, 21, 22, 23, 24, 25, 26

On Day 1 of each week each patient will complete the following procedures:

- Adverse events since last visit
- Concomitant medication
- 12-lead ECG (Weeks 13, 21)
- Vital signs
- Blood sampling for clinical chemistry (Weeks 13, 15, 21, 24)
- Pregnancy test (urine or blood, Weeks 13, 21)

Dosing/treatment:

- Expansion Arm A: MVASI (Week 7, 11, 13, 15, 17, 19, 21, 23, 25)
- Expansion Arm C: KEYTRUDA (Week 7, 10, 13, 16, 19, 22, 25)
- Weekly change of infusion pump container

9.1.5 Treatment Period: Week 9, 18 and End of Treatment

On Day 1 of each week each patient will complete the following procedures and assessments:

- Adverse events since last visit
- Concomitant medication
- Blood sampling
  - Clinical chemistry
  - Hematology

  - Pharmacokinetics and immunogenicity (Week 9 only)
  - Biomarkers
- Pregnancy test (urine or blood)
- Physical examination (EOT only)
- 12-lead ECG
- Vital signs
- Neurological examination (NANO scale)
- Quality of life assessments: EORTC-QLQ C30, BN 20 (Week 9 and at EOT)
- Response assessments (at EOT only if regular end of treatment after 26 weeks):
  - Imaging (brain MRI including multiparametric sequences)
  - RANO assessment

Dosing/treatment

- Weeks 9: Expansion Arm A: MVASI
- Weeks 9 and 18: change of infusion container
- After Week 26, at the end of treatment or at the discretion of the patient and/or investigator: removal of infusion pump and explant port-a-cat

9.1.6 Follow-up

Every 3 months each patient will complete the following procedures and assessments:

- Concomitant medication
- Blood sampling
  - Clinical chemistry
  - Hematology
  - Biomarkers
  - Immunogenicity (Month 9 and 12 only)
- Physical examination
- Vital signs
- Neurological examination (NANO scale)
- Quality of life assessments: EORTC-QLQ C30, BN 20
- Response assessments:
  - Imaging (brain MRI including multiparametric sequences)
  - RANO assessment
- Survival (for max. 24 months after first dose).

In case the patient is no longer able to come to the site, the patient will be followed up for survival only.

Patients starting a new therapy:

Patients starting a new therapy will be followed up for survival. The investigator will review all new anti-cancer therapy initiated after the last dose of study treatments. Information regarding new anti-cancer treatments will be collected and recorded in the eCRF. The assessments listed above will not be performed except for MRI. Whenever possible the MRI under new treatment shall be performed with the parameters defined for this study. The frequency of MRIs and FU visits can be adapted to the schedule of the new therapy (e.g. every 2 months).

9.1.7 Premature Study Termination

In case of premature discontinuation during the treatment phase, the investigator shall try to arrange a final visit and perform examinations according to the end of treatment visit. If the patient does not enter the follow-up this is the study termination visit.

In case of premature discontinuation during the follow-up phase, the investigator shall try to arrange a final visit and perform examinations according to the follow-up visit. This is then the study termination visit.

9.1.8 Time Windows

Study treatment days, EOT:	± 2 days
Follow-up:	± 7 days<br>in case of new therapy, the visit schedule might be adapted to the new therapy
Dosing interruptions in the case of medical / surgical events or logistical reasons not related to study therapy:	not more than 2 consecutive days and / or 7 days in total
MRI scans:	up to 3 days before or after the scheduled day

9.2 Efficacy Assessments

9.2.1 Response Assessments

Response will be assessed starting from baseline (screening) during treatment phase and in follow-up visits according to updated RANO criteria (including MRI and clinical evaluation (37).

Response may be categorized as complete response (CR), partial response (PR), stable disease (SD), progressive disease (PD).

9.2.2 Tumor Vascularization

Tumor vascularization will be assessed per vascular MRI scans. Changes from baseline in tumor vascularization over time will be evaluated. Details will be described in the imaging manual. Comparative analyses (e.g. perfusion, diffusion, rCBV) using a population of retrospective matched patients who received standard of care treatment (i.e. radiotherapy plus temozolomide) are planned.

9.2.3 Neurological Examination

Neurological examination will be done according to NANO scales (38).

9.2.4 Time to Event Time Points

Progression-free survival and overall survival will be assessed.

9.2.5 Quality of Life Assessments

Quality of life will be assessed using the validated quality of life scale (QLQ-C30) by the EORTC as well as the module for brain tumors, BN 20. The global score as well as the functional scales and the symptom scales will be analyzed.

Site staff will give the patients these questionnaires to complete. Site staff will collect the questionnaires and check them for completeness, but site staff may not interpret the questionnaire for the patient.

9.2.6 Pharmacokinetics

Olaptesed pegol concentration will be quantified in plasma prior to and during therapy.

Two plasma aliquots will be prepared at each time point according to the procedure described in the laboratory manual and stored at the clinical site at or below -65 °C. One aliquot will be sent for analysis to a third-party service laboratory and the other will be kept at the clinical site.

9.2.7 Pharmacodynamics

9.2.7.1 CXCL12 (SDF-1)

Basal plasma concentration of CXCL12 will be quantified in platelet poor plasma. CXCL12 cannot be used as a biomarker after dosing with olaptesed pegol.

Two plasma aliquots will be prepared. One aliquot will be sent for analysis to a third-party service laboratory and the other will be kept at the clinical site.

9.2.7.2 Additional Biomarkers

Serum and whole blood samples will be collected for further optional exploratory biomarker analyses. Whole blood samples will be collected for the expansion group only. Tissue from previous biopsy or resection or from re-biopsy / re-resection, if performed during the study, will be used for optional exploratory biomarker analyses.

9.2.8 Pharmacogenetics

Understanding genetic determinants of drug response is an important endeavor during medical research. This research will evaluate whether genetic variation within a clinical study population correlates with response to the treatment(s) under evaluation. If genetic variation is found to predict efficacy or adverse events, the data might inform optimal use of therapies in the patient population. This research contributes to understanding genetic determinants of efficacy and safety associated with the treatments in this study.

It is planned to store a blood sample for pharmacogenetic analyses, which may be recognized in the future of being important for drug development and treatment. The variations in genes related to CXCL12 or receptors CXCR4 and CXCR7 will be determined if data suggest a more detailed analysis of inter-individual differences regarding safety, efficacy or PK assessments. If the patient agreed on the collection of the pharmacogenetic sample, a whole blood sample will be collected according to the procedure described in the laboratory manual and stored at the clinical site at or below -65 °C. After completion of the study, samples may be transferred to a qualified third-party provider for long term storage. According to the patient informed consent, samples may be stored for up to 5 years and will be disposed after approval by the sponsor according to EU and national laws for biological material.

9.3 Demographics and Medical History

Demographic data to be collected for this study include:

- Gender
- Age
- Body weight (kg)
- Height (cm)
- Ethnicity
- Body Mass Index (kg/m²) will be calculated based on height and weight
- Degree of resection (biopsy only, partial, complete)

Body weight and height will be recorded once only during the screening visit. Measurement of weight will be done with the patient wearing regular clothes and shoes.

The medical history comprises all relevant medical conditions/illnesses in the past and present at the start of the study.

9.4 Safety Assessments

9.4.1 Physical Examination

The following body systems will be examined at physical examination: eyes, ears, nose, throat including thyroid, size of liver and spleen, neck; respiratory system; central and peripheral nervous system; cardiovascular system; gastrointestinal system including mouth; musculoskeletal system; skin; other appropriate systems (related to Medical History). Rectal, genitourinary, and breast exams are not to be routinely performed (i.e. only on a “for cause” basis). Additional physical examinations can be performed throughout the study whenever deemed necessary by the investigator. Values determined to be clinically significant according to the Investigator will be recorded as an AE.

9.4.2 Vital Signs

Vital signs include blood pressure and heart rate. Values determined to be clinically significant according to the Investigator will be recorded as an AE.

Measurement of blood pressure will be carried out after at least 5 minutes of resting in the supine position and whenever possible always on the same arm. If there is any suspicion of unreliable measurement, blood pressure will be measured again. This value will be considered as definitive and will be recorded.

9.4.3 ECG

A standard 12-lead ECGs will be recorded. Standard ECG parameters (e.g. P, PR, QRS, QT, QTcBazett etc.) and heart rate will be analyzed.

The ECG will be carried out after at least 5 minutes of resting in the supine position by qualified staff members. Each ECG recording will be printed as an original rhythm strip with at least 5

evaluable ECG complexes (cardiac cycles) for each lead. A print-out will be kept as source data, this print-out needs to be signed and dated by one of the investigators after the ECG has been reviewed. In case of any abnormal findings, the reviewing investigator needs to assess these finding in respect to their clinical relevance. Values determined to be clinically significant according to the Investigator will be recorded as an AE.

9.4.4 Clinical Laboratory Evaluation

The following laboratory parameters will be assessed in each patient using validated standard methods. Values determined to be clinically significant according to the Investigator will be recorded as an AE.

- Clinical chemistry: AST, ALT, AP, GGT, creatine kinase, lactate dehydrogenase (LDH), albumin, total bilirubin, glucose, blood urea nitrogen (BUN), urea, creatinine, total protein, chloride, uric acid, CRP, calcium, sodium, potassium
- Hematology: hemoglobin, hematocrit, MCH, MCV, MCHC, RBC, WBC, platelet count, neutrophils, basophils, eosinophils, lymphocytes, monocytes
- Coagulation: prothrombin time (PT) and activated partial thromboplastin time (aPTT). In case of prolonged aPTT, measurement of individual clotting factors VIII, IX, XI and XII will be performed.

9.4.5 Pregnancy Test

At screening, plasma from venous blood samples will be analyzed for Human Chorionic Gonadotropin (hCG) to assess pregnancy status in females of childbearing potential. All women, including those with tubal ligation, are considered to be of childbearing potential unless they have been postmenopausal for at least 2 years. Hysterectomized women are considered surgically sterile and are not required to use any contraception. Samples will be disposed by the laboratory after completion of analysis. Pregnancy tests during the treatment period may be performed via urine test or blood test.

9.4.6 Immunogenicity

Serum samples will be analyzed for potential antibodies to olaptesed pegol.

Two serum aliquots will be prepared at each sample collection time point according to the procedure described in the laboratory manual and stored at the clinical site at or below -65°C. One aliquot will be sent for analysis to a third-party service laboratory and the other will be kept at the clinical site.

10. Pharmacovigilance

10.1 Definition of Adverse Events

An adverse event (AE) is defined as any untoward medical occurrence in a patient administered a product and which does not necessarily have a causal relationship with this treatment. An AE can therefore be any new sign, symptom, illness, or syndrome, any abnormal laboratory values, if judged clinically significant in the opinion of the investigator, any worsening (change in nature, severity or frequency) of a concomitant or pre-existing illness, any adverse effect of the investigational medicinal product, including comparator or concomitant medication, any drug interactions, any adverse effect of an invasive procedure required by the protocol or any accident or injury, whether or not related to the medicinal (investigational) product.

All AEs fall into one of two categories: “non-serious” and “serious”.

In this study, the following medical events will not be considered to fall under the definition of an AE:

- Surgical procedures or other therapeutic interventions themselves are not AEs, but the condition for which the surgery/intervention is required is an AE and should be documented accordingly.
- Planned surgical measures and the condition(s) leading to these measures are not AEs, if the condition(s) was (were) known before the period of observation and did not worsen during study. In the latter case, the condition should be reported as medical history.
- Any serious adverse events (SAEs) detected by the investigator in a study patient after the end of the period of observation, which the investigator does not consider to be related to prior study treatment or procedures
- Progression of the cancer under study

10.2 Definition of Serious Adverse Events

A serious adverse event (SAE) is any untoward medical occurrence that at any dose (including overdose):

- Results in death
- Is life-threatening, i.e. the patient was at immediate risk of death at the time of the SAE; it does not refer to a SAE that hypothetically might have caused death if it were more severe.
- Requires in-patient hospitalization or prolongation of existing hospitalization. This means that hospital in-patient admission or prolongation of hospital stay was required for the treatment of the AE, or that they occurred as a consequence of the event. Visits to a hospital by ambulance or to the emergency room without admission will not be regarded as hospitalization unless the event fulfills any other of the serious criteria
- Results in persistent or significant disability or incapacity, i.e. a permanent or significant and substantial disruption of a person’s ability to carry out normal life functions.
- Is a congenital anomaly or birth defect

- Is an important medical event. Medical and scientific judgment should be exercised in deciding whether expedited reporting is appropriate in situations where none of the outcomes listed above occurred. Important medical events that may not be immediately life-threatening or result in death or hospitalization but may jeopardize the patient or may require intervention to prevent one of the other outcomes listed in the definition above should also usually be considered serious. Examples of such events include allergic bronchospasm requiring intensive treatment in an emergency room or at home, blood dyscrasias, or convulsions that do not result in in-patient hospitalization, or the development of drug dependency or drug abuse. A diagnosis of cancer/ malignant tumor during the course of a treatment should always be considered as medically important.

Clarification of the difference in meaning between “severe” and “serious”: The term “severe” is often used to describe the intensity (severity) of a specific event (as in mild, moderate, or severe myocardial infarction); the event itself, however, may be of relatively minor medical significance (such as severe headache). This is not the same as “serious”, which is based on the outcome or action criteria usually associated with events that pose a threat to life or functioning. Seriousness (not severity) serves as a guide for defining regulatory reporting obligations.

Other events to be reported on the SAE report form:

- Misuse and overdose: Drug misuse and drug overdose should be reported in the same format and within the same timelines as an SAE, even if they may not result in an adverse outcome. If no SAE is associated, the case will be regarded as non-serious.
- Exposure to drug during pregnancy or lactation: In principle, pregnancy and the lactation period are exclusion criteria for clinical studies involving investigational drugs, which are not directly related to the respective conditions. In the event of a pregnancy occurring during the course of this particular study, the patient should be withdrawn from study, but closely followed-up during the entire course of the pregnancy and postpartum period. All recommendations described in the investigational drug brochure during pregnancy and lactation have to be carefully considered.

The sponsor must be notified without delay. Parental and neonatal outcomes must be recorded even if they are completely normal and without Adverse Events. Off-spring should be followed up for at least 8 weeks after delivery.

Longer observation periods may be determined by the sponsor if an adverse outcome of the pregnancy was observed.

10.3 Period of Observation

All adverse events that occur after the consent form is signed but before treatment with IMP must be reported by the investigator if they cause the patient to be excluded from the study or are the result of a protocol-specified intervention, including but not limited to washout or discontinuation of usual therapy, diet or a procedure.

For the purposes of this study, the period of observation for collection of AEs extends from the time when the patient signed informed consent until 30 days after the last olaptesed pegol administration. For SAEs, the observation period extends to 90 days after the last treatment and for pregnancies to 120 days after the last treatment.

If the Investigator detects a SAE in a study patient after the end of the period of observation and considers the event possibly related to prior study treatment or procedures, he or she should contact the sponsor to determine how the AE should be documented and reported.

All AEs that occur in the course of a clinical study regardless of the causal relationship must be monitored and followed up until the outcome is known as long as the patient is in the study. There must be documented reasonable attempts to get this information. It is the responsibility of the Investigator to ensure that any necessary additional therapeutic measures and follow-up procedures are performed.

Association of an event with the IMP is excluded if the event occurs later than the following latency period after discontinuation of olaptesed infusion:

- 30 days for non-serious adverse events,
- 90 days for SAEs,
- 120 days for pregnancy,
- 30 days if subject initiates a new cancer therapy, whichever is earlier.

If events occur after these latency periods, they will not be recorded.

10.4 Recommendations to treat Overdosing and Intoxication with the Study Medication

No drug-specific antidote is available. Other emergencies will be treated symptomatically according to standard medical practice.

10.5 Documentation and Reporting of Adverse Events

All AEs (whether serious or non-serious) must be documented in the eCRF. Additional documentation of SAEs is described in section 10.7.

The Investigator must document all AEs that occur during the observation period set on the respective pages provided in the eCRF in accordance with the instructions for the completion of adverse event reports in clinical studies. These instructions are provided in the Investigator file and in the eCRF itself.

Every attempt should be made to describe all AEs in terms of a diagnosis. If a clear diagnosis has been made, individual signs and symptoms should not be recorded unless they represent atypical or extreme manifestations of the diagnosis, in which case they should be reported as separate events. If a clear diagnosis cannot be established, each sign and symptom must be recorded individually.

10.5.1 Nature

The **nature** of the event will be described in precise, standard medical terminology (i.e. not necessarily the exact words used by the patient). If known, a specific diagnosis should be stated (e.g. flue like symptoms).

10.5.2 Severity

The **severity** of the adverse event will be described in terms of Grades according to according to the Common Terminology Criteria for Adverse Events (CTCAE v.5.0) and according to the investigator's clinical judgment.

• Grade 1:	mild	asymptomatic or mild symptoms; clinical or diagnostic observations only; intervention not indicated.
• Grade 2:	moderate	minimal, local or noninvasive intervention indicated; limiting age-appropriate instrumental activities of daily life (ADL)[1]
• Grade 3:	severe or medically significant but not immediately life-threatening	hospitalization or prolongation of hospitalization indicated; disabling; limiting self-care ADL[2]
• Grade 4:	life-threatening consequences	urgent intervention indicated
• Grade 5:	death related to AE	-

10.5.3 Duration

The **duration** of the event will be described by the start date and end date.

For SAEs, the start date is the date on which the event became serious. The end date of an SAE is the date when the event resolved (not when serious criteria were no longer fulfilled).

10.5.4 Relationship

The **causal relationship** of the event to use of the study medication will be described according to the WHO-UMC system for standardized case causality assessment as presented in Table 10-1. All points of the assessment criteria should be reasonably complied with.

[1] Instrumental ADL refer to preparing meals, shopping for groceries or clothes, using the telephone, managing money, etc.
[2] Self-care ADL refer to bathing, dressing and undressing, feeding self, using the toilet, taking medications, and not bedridden.

Table 10-1: WHO-UMC assessment criteria

Causality term	Assessment criteria	E2B (EudraVigilance Database / EMA)
Certain	• Event or laboratory test abnormality, with plausible time relationship to drug intake<br>• Cannot be explained by disease or other drugs<br>• Response to withdrawal plausible (pharmacologically, pathologically)<br>• Event definitive pharmacologically or phenomenologically (i.e. an objective and specific medical disorder or a recognized pharmacological phenomenon)<br>• Re-challenge satisfactory, if necessary	Reasonable possibility
Probable/ Likely	• Event or laboratory test abnormality, with reasonable time relationship to drug intake<br>• Unlikely to be attributed to disease or other drugs<br>• Response to withdrawal clinically reasonable<br>• Re-challenge not required	
Possible	• Event or laboratory test abnormality, with reasonable time relationship to drug intake<br>• Could also be explained by disease or other drugs<br>• Information on drug withdrawal may be lacking or unclear	
Unlikely	• Event or laboratory test abnormality, with a time to drug intake that makes a relationship improbable (but not impossible)<br>• Disease or other drugs provide plausible explanations	No reasonable possibility
Unrelated	• There is no evidence or argument to suggest a causal relationship.	

For regulatory E2B reporting, this assessment will be converted to the binary system of “reasonable possibility” (certain, probable/likely, possible) and “no reasonable possibility” (unlikely or unrelated). The expression “reasonable possibility” means to convey in general that there is factual evidence or argument to suggest a causal relationship. The assessment will be documented on the AE and SAE form.

10.5.5 Outcome

The **outcome** of the event will be described in terms of:

- Recovered: fully recovered or by medical or surgical treatment the condition has returned to the level observed at the first study related activity after the patient signed the informed consent
- Recovering: the condition is improving, and the patient is expected to recover from the event. This term should only be used when the patient has completed the study

- Recovered with sequelae: as a result of the AE, the patient suffered persistent and significant disability/incapacity (e.g. became blind, deaf, paralyzed). Any AE recovered with sequelae should be rated as an SAE
- Not recovered
- Fatal
- Unknown: This term should only be used in cases where the patient is lost to follow-up.

Insofar as possible all adverse events should be followed-up to determine the final outcome of the event. Details of follow-up should be recorded in the eCRF and the SAE report form, if applicable (e.g. discontinuation of study medication, if specific treatment is required, if hospitalization is required etc.).

10.6 Documentation and Reporting of Serious Adverse Events

If the adverse event is serious, the Investigator must complete in addition to the "adverse event" eCRF page a "SAE report form" at the time the SAE is detected. This form must be sent immediately, i.e. within 24 hours upon becoming aware of the SAE to the safety contact of the sponsor [redacted] The Investigator will document the date when any employee had first been aware of the report and fax all SAE reports (initial and follow up reports) even if they are incomplete within one day upon receipt to the safety department of the sponsor, represented by:

[redacted]

When an "overdose" or "drug misuse" of the investigational product occurs without an adverse event, the investigator should only complete a SAE report form and send this to the sponsor's safety contact. It should be clearly stated that no AE was observed. In this case, there is no need to complete the AE page in the eCRF. The event will not be considered as serious.

Likewise, if a pregnancy is detected, the investigator should only complete a SAE report form and send this to the sponsor's safety contact. It should be clearly stated that no AE was observed. In this case, there is no need to complete the AE page in the eCRF. A pregnancy will not be considered as serious as long as there is no serious adverse outcome.

The initial report should be as complete as possible, including details of the current illness and (serious) AE, the reason why the event was considered serious, date of onset and stop date (if applicable), diagnostic procedures and treatment of the event, relevant medical history and concomitant medication and action taken with study medication.

Every attempt should be made to describe all AEs in terms of a diagnosis. If a clear diagnosis has been made, individual signs and symptoms should not be recorded unless they represent atypical or extreme manifestations of the diagnosis, in which case they should be reported as

separate events. If a clear diagnosis cannot be established, each sign and symptom must be recorded individually.

For the time period beginning at treatment allocation through 90 days following cessation of treatment, or 30 days following cessation of treatment if the patient initiates new anticancer therapy, whichever is earlier, any SAE, or follow up to a SAE, including death due to any cause other than progression of the cancer under study, whether or not related to the Sponsor's product, must be reported.

10.7 Reporting of Pregnancy and Lactation to the Sponsor

Although pregnancy and lactation are not considered adverse events, it is the responsibility of investigators or their designees to report any pregnancy or lactation in a patient (spontaneously reported to them) that occurs during the study.

Pregnancies and lactations that occur after the consent form is signed but before treatment with IMP must be reported by the investigator if they cause the patient to be excluded from the study or are the result of a protocol-specified intervention, including but not limited to washout or discontinuation of usual therapy, diet, placebo treatment or a procedure.

Pregnancies and lactations that occur from the time of treatment allocation/randomization through 120 days following cessation of IMP, or 30 days following cessation of treatment if the patient initiates new anticancer therapy, whichever is earlier, must be reported by the investigator. All reported pregnancies must be followed to the completion/termination of the pregnancy. Pregnancy outcomes of spontaneous abortion, missed abortion, benign hydatidiform mole, blighted ovum, fetal death, intrauterine death, miscarriage and stillbirth must be reported as serious events (Important Medical Events). If the pregnancy continues to term, the outcome (health of infant) must also be reported.

Such events must be reported within 24 hours to the Sponsor on the SAE report form to the e-mail or fax number given in section 10.7.

10.8 Follow-up of Missing Information

Information not available at the time of the initial report (e.g., an end date for the AE or laboratory values received after the report) must be documented on a “Serious Adverse Event” form, with the box “Follow-up” checked under “Report type”.

All patients who have an AE, whether considered associated with the use of the investigational products or not, must be monitored to determine the outcome. The clinical course of the AE will be followed up according to accepted standards of medical practice, even after the end of the period of observation, until a satisfactory explanation is found, or the Investigator considers it medically justifiable to terminate follow-up.

Should the AE result in death, a full pathologist’s report should be supplied, if possible.

The sponsor will identify missing information for each SAE report. Requests for follow up will be sent to the CRO for further processing. The CRO will require follow up information in regular intervals from the Investigators until all queries are resolved or no further information can be

reasonably expected. All responses to queries and supply of additional information by the Investigator should follow the same reporting route and timelines as the initial report.

10.9 Deaths

In the case a patient dies, the investigator will document the cause of death in the eCRF page "End of Study". If possible, a death certificate should be obtained with the cause of death evaluated and documented.

10.10 Expedited Reporting of Adverse Events

10.10.1 SUSAR Reporting

The reference safety information (RSI) is given in the IB "Olaptesed pegol" in its current edition.

The sponsor will report all serious and unexpected AEs, which, on the basis of the RSI, are judged by either the investigator or the sponsor as having a reasonable suspected causal relationship i.e. suspected unexpected serious adverse reaction - SUSAR, to the CAs and the concerned IRB/IEC according to applicable law.

The Sponsor will notify the Investigator of study product SUSARs in accordance with the local requirements.

10.10.2 Development Safety Update Reports

The sponsor or sponsor representative will prepare and submit DSURs to CAs and concerned IRB/IECs.

11. Statistical Methods

11.1 Determination of Sample Size

No formal sample size calculations were performed for this standard dose-escalation 3+3 design. In addition, a further 12 patients will be enrolled (Arm A: 6 patients; Arm B: 6 patients) to obtain first results for a combined therapy with bevacizumab (Arm A) and fully resected (Arm B). Analyses for all endpoints will be performed descriptively. Another 6 patients will be enrolled (Arm C) to obtain first results for a combined therapy with pembrolizumab. No hypotheses will be tested.

The primary endpoint of this study is the safety in terms of adverse events with focus on DLTs. Efficacy will be assessed as exploratory analysis.

This dose-escalation study is designed to enroll successive cohorts of patients (at least 3 patients/cohort) with three dose levels, 200, 400, and 600 mg/week. Dose escalation is to be halted when the maximum tolerated dose (MTD) was reached. The MTD was defined as the dose level below that at which the DLT was observed in one-third or more patients. If one of the patients in a dose cohort experienced a DLT, three more patients are to be added to the cohort. If no further DLT is observed in the group, an additional cohort of three patients is to be enrolled at the next higher dose.

11.2 Statistical and Analytical Plans

All items of the eCRF will be presented in individual patient data listings and in appropriate summary tables. Individual patient data listings will be presented per parameter and will be sorted appropriately. Further details of the planned analyses will be given in the statistical analysis plan (SAP).

11.3 Summary of Variables

Continuous variables will be summarized by descriptive statistics in terms of means, standard deviation, median, the first and third quartile, minimum and maximum including N, N(missing),. Frequency tables will be drawn for categorical variables using counts and percentages. Summary tables will be displayed by cohort and visit (if applicable). Where appropriate the presentation will include changes from baseline and shift tables.

Time-to-event endpoints will be analyzed by using Kaplan-Meier-Methods. Kaplan-Meier plots will also be provided.

11.4 Data Sets to be Analyzed

11.4.1 Safety Analysis Set

Enrolled patients who received at least olaptesed pegol for any duration form the Safety Analysis Set (SAF). The SAF will be used for all analyses of safety, tolerability, baseline and background characteristics.

11.4.2 Full Analysis Set

The Full Analysis Set (FAS) is a subset of the SAF consisting of all patients for whom both baseline data and data on the efficacy variable at some post-baseline visit is available. The FAS will be used for the analysis of efficacy.

11.5 Analysis of Study Conduct and Patient Disposition

The disposition of patients and analysis sets, inclusion and exclusion criteria, and the status at study termination will be shown.

11.6 Analysis of Baseline and Background Characteristics

All screening and baseline summaries will be based on the SAF if not indicated otherwise.

Baseline demographic and disease characteristics (including medical history) will be summarized. Demographic characteristics will include gender, age, height, weight, BMI and ethnic origin. Medical History will be documented and assessed in the scope of overall safety.

Previous and concomitant medications will be coded according to WHO-DD and the verbatim will be tabulated together with the Anatomical Therapeutic Chemical (ATC) level 2 classification as well as the WHO-DD preferred term (PT).

11.7 Efficacy Analyses

Efficacy analyses will be based on the FAS if not indicated otherwise. Efficacy parameters will be summarized by cohort/arm and visit. Absolute changes from baseline/ pre-dose will be calculated where appropriate (e.g. quality of life score) and will be summarized descriptively.

Methods for survival analysis will be applied for evaluation of time to event endpoints, which adequately takes censored information (i.e. observations from patients who were withdrawn or lost to follow up) into account.

The analysis will be regarded exploratory only.

11.8 Safety Analyses

Safety analyses will be based on the SAF if not indicated otherwise. The corresponding safety variables are specified below.

11.8.1 Adverse Events

AEs will be summarized by System Organ Class (SOC) and preferred term. Coding will be based on the MedDRA. The proportion of patients experiencing at least one treatment emergent adverse event (TEAE) and the proportion of patients experiencing each type of TEAE will be summarized. A summary by severity and relatedness will be provided, additionally. Deaths, serious adverse events and adverse events with a causal relationship to the study medication will be presented separately.

11.8.2 Clinical Laboratory

Where applicable, recorded values in hematology and clinical chemistry will be summarized descriptively for each time point. The numbers of readings below, within and above normal range will be presented in frequency tables. Absolute and relative changes calculated to the values collected at baseline and/or pre-dose will also be summarized descriptively for quantitative parameters.

11.8.3 Physical Examination and Other Safety Measures

Physical examination results will be listed and summarized where applicable. Vital signs and ECG will be summarized descriptively for each scheduled protocol time point. Absolute changes calculated relative to the assessments at baseline will also be summarized descriptively.

11.9 Pharmacokinetics

Individual olaptesed pegol plasma concentration data will be listed. Concentration data will be summarized for each time point.

11.10 Pharmacodynamics

All PD variables will be evaluated by applying appropriate descriptive statistics including absolute and relative changes from baseline, if applicable.

11.11 Subgroup Analyses

Subgroup analysis will be conducted for patients receiving combination treatment with bevacizumab or pembrolizumab, further details will be described in the SAP.

11.12 Interim Analyses

Not applicable.

11.13 Handling of Dropouts and Missing Data

Patients dropping out of the study might be replaced according to chapter 8.4.4. Whenever possible reasons for dropping out will be ascertained and recorded. The data of dropouts will be included in the safety analysis and in the efficacy analysis as far as necessary data are available. In general, other than partial dates (for example to calculate date differences) there will be no imputation of missing data. In time to event analyses missing data will be taken into account by censoring.

12. Data Collection, Handling, and Record Keeping

12.1 Generation of Data Base

The eCRF will be supplied by the CRO and should be handled in accordance with the instructions provided. eCRFs will be provided as regulatory compliant, electronically secure and protected, web-based database. An audit trail will record all entries and corresponding changes.

All eCRFs should be filled out completely by qualified personnel or authorized study staff. Only authorized persons will be granted access. After the last query for a patient is closed, the eCRF is reviewed by the investigator and signed electronically.

12.2 Data Collection

All study data are to be recorded in the eCRF.

All the patient's data have to be reported on the eCRF in an pseudonymized fashion, the patient only being identified by the patient number.

The investigator will be responsible for the completeness, accuracy and legibility of the information in the eCRF and other study documents. Data derived from source documents are to be consistent with these source documents.

The study monitors then have to check the eCRF against the source documents for accuracy and validity according to the monitoring schedule, as applicable.

Upon completion of the examination, eCRF completion is expected at each site to ensure quality of data and patient safety. Once eCRF are completed, they will be available for review by the monitor and the clinical data management. Completed eCRF will be reviewed remotely for logical discrepancies. The monitor will ensure that all data queries and subsequent amendments in the eCRF documentation are made according to GCP guidelines.

12.2.1 Source Data

Source data is all information, original records of clinical findings, observations, or other activities in a clinical study necessary for the reconstruction and evaluation of the study. Source data are contained in source documents. Examples of these original documents and data records include: hospital records, physician's and nurse's notes, clinical and office charts, laboratory notes, pharmacy dispensing records, recorded data from automated instruments, consultant letters, screening and enrolment log, etc.

The investigator is responsible to keep all study-related source data enabling the sponsor to reconstruct the complete course of the clinical study. Source data will be transcribed to and reported through the CRF accordingly.

12.3 Data Management

All data management activities will be done according to ICH-GCP as required by regulatory agencies. Responsibility for data management lies with the designated CRO following their internal standard operating procedures (SOPs).

The designated CRO will be responsible for the activities associated with the data management of this study, including the production of an eCRF, setting up a relevant database, along with appropriate validation of data and resolution of queries. All data will be entered into the eCRF. Automated and manual checks will be made against the data to ensure completeness and consistency.

AEs will be standardized for terminology and classification, using MedDRA Dictionary. Concomitant medications will be classified by site of action and therapeutic and clinical characteristics using the WHO-DRUG dictionary. Clean data sets will be provided for statistical analysis and reporting.

12.4 Data Protection

The data obtained in the course of the study will be treated pursuant to the EU General Data Protection Regulation (GDPR) (EU Datenschutz-Grundverordnung (DSGVO)) and national laws on data protection.

Generally, all patient related data is recorded in a pseudonymized manner to effectively mitigate the possibility to attribute clinical data to a person in accordance with data protection regulations.

During the clinical study, patients will be identified solely by means of their individual identification code (Patient ID). Study data stored on a computer will be stored in accordance with local data protection law and will be handled in strictest confidence (e.g. preventing unauthorized use of the system by password-protection, limited-rights management of authorized personnel, security screening of access to data, access monitoring and an access log of patient data). Distribution of these data to unauthorized persons has to be prevented strictly. Data from patients including imaging analyses, central lab measurements and their results is therefore only accessible to designated personnel on the study and is password protected. The appropriate regulations of local data legislation will be fulfilled in its entirety.

The patient consents in writing to release the investigator from his/her professional discretion in so far as to allow inspection of original data for monitoring purposes by health authorities and authorized persons (inspectors, clinical monitors, auditors). Authorized persons (inspectors, clinical monitors, auditors) may inspect the patient-related data collected during the study in accordance with the data protection law.

The investigator will maintain a patient identification list (Patient IDs with the corresponding patient names) to enable records to be identified. This list will be stored in the clinical data repository, which is an access-restricted room. Patients who do not consent to circulate their pseudonymized data will not be included into the study.

This protocol, the CRFs and other study-related documents and material must be handled with strict confidentiality and must not be disclosed to third parties except with the express prior consent of Sponsor. In particular, it must be ensured that the study medication is kept out of

reach of third parties. Staffs of the investigators involved in this study are also bound by this agreement.

Electronic data will be handled according to best business practices including but not limited to, use of appropriate system login details (e.g. for data capturing and management systems), systemic up-to-date virus/malware detection, firewalls, and back-up and restore processes.

In this study, a Remote Data Entry (RDE) system is used for data processing using an audit trail. Data is stored on servers behind restrictive firewalls in the data center of the university hospital. Users are connected via a secure 256 bit-RSA encryption. The concept of IT-security includes a data-backup system that backs up clinical study data on a daily basis.

In accordance with NOXXON Pharma AG SOPs, vendors' processes and systems are regularly audited to check their compliance with clinical study related guidelines and regulations and data protection regulations. Reporting procedures include the reporting of breaches as well, enabling NOXXON Pharma AG to meet such regulatory expectations.

In the unlikely event that unpseudonymized personal patient data would be received by the sponsor, those would be immediately destroyed by the recipient according to the sponsor's policy.

If the study personnel at the clinical study site becomes aware that a personal data breach has occurred the competent supervisory authority (University Hospital Administration, legal department and law-enforcement institutions) should be notified immediately, i.e. no later than 72 hours after having become aware of it. In the case of the breach is unlikely to result in a risk for the health, rights and freedoms of individuals, the next steps can be discussed and actions to prevent further breaches are installed. The notification of a breach should contain the following key components:

An explanation of the personal data breach, including the form and extent of the respective data concerned, the name and contact details of the site's Data Controller's Data Protection Officer, an estimation of the possible consequences of the personal data breach.

In addition, a short description of the measures taken by the controller to address the personal data breach, including, where appropriate, to mitigate its possible adverse effects.

Subjects are to be notified if the personal data breach is likely to result in a high risk to their rights and freedoms, in order to allow them to take the precautions and enforce legal alterations to protect further damage. Within the scope of this study, no sequencing data or other sensitive high-throughput data is generated. Therefore, the possible effects of a data breach lie within the personal clinical data.

Specific measures after theft or breaches of personal medical information include the information of the respective authorities, supporting the fast identification of the source of the information breach and support in retrieving the stolen information.

13. Quality Assurance

13.1 Data Monitoring

Monitoring visits will follow monitoring procedures developed by the designated CRO, as regularly as it is necessary during the conduct of the study until the last eCRFs have been completed and all queries have been resolved, in order to comply with GCP and to ensure acceptability of the study data for international registration purpose.

Regular monitoring visits by representatives of the Sponsor and/or entrusted CRO at the investigator's site prior to the start and during the course of the study will aid in following the study's progress, ensure utmost accuracy of the data and allow for early detection of possible errors. The investigator or supervising physician permits sponsor's employees who are supervising the clinical study (e.g. CRAs) to regularly examine the case report forms and ask questions about further records relevant to the study. Quality control serves to ensure correct employment of the study medication, adherence to the study protocol and the completeness, plausibility and utility of the data entered in the case report forms. The cooperation between the investigator or his/her colleagues and the CRA will take into account the current status of GCP (Good Clinical Practice) requirements.

For quality assurance, the authorities require a direct comparison between the data recorded in the eCRF and the source data compiled by the investigator. For this purpose, the investigators will allow the CRA of the Sponsor and/or the CRO to inspect the source data compiled for this study. According to legal requirements, it is also possible that the Quality Assurance Department of the CRO and of the Sponsor, or a designated contract auditor or a Regulatory Authority, may want to inspect the logistical procedures as well as to scrutinize the data (audit or inspection). The investigator will allow the persons responsible for the audit or the inspection to have access to the source data and documents and will answer any questions.

13.2 Audits and Inspections

The investigator will permit study-related audits, and inspections by the IRB/IEC, the sponsor, and regulatory authorities of all study-related documents (e.g. source documents, regulatory documents, data collection instruments, study data etc.). The investigator will ensure the availability of applicable study-related facilities (e.g. pharmacy, diagnostic laboratory, etc.) for inspections.

Audits and inspections will be performed in accordance with ICH-GCP, EU Directives, and applicable local regulations to ensure that the clinical study is conducted in compliance with the study protocol requirements.

14. Ethical and Legal Requirements

14.1 Independent Ethics Committee

The study will only start after approval of the study protocol and all relevant documentation by the IRB/IEC of the participating countries in accordance with applicable regulatory and local requirements.

After completion or termination of the study, the results will be submitted to the IRB/IEC. The study is completed with the last visit of last patient.

14.2 Ethical Conduct of the Study

This study will be conducted in compliance with the study protocol, the ethical principles originating in or derived from the Declaration of Helsinki and in compliance with IRB/IEC, informed consent regulations, and International Conference on Harmonization (ICH) Good Clinical Practices (GCP) Guidelines (63,64). In addition, all local legal and regulatory requirements will be followed. NOXXON Pharma AG will provide appropriate insurance coverage for the patients, in accordance with legal requirements and local law. An insurance certificate will be provided for the investigator's site file. Patients are to be informed by the investigator of the existence of such insurance and that they have the right to inspect the terms and conditions of said insurance or may receive a copy, if required per national legislation.

14.3 Changes in the Conduct of the Study

Changes to the protocol during the study will be documented as amendments. The amended protocol will be signed by the relevant personnel at NOXXON Pharma AG and by the Investigator. Depending on the contents of the amendment and local legal requirements, the amendment will be submitted to the relevant IRB/IEC and, where necessary, to the relevant competent authorities. The Investigator should not implement any deviation from, or changes of the protocol, without agreement by NOXXON Pharma AG and prior review and documented approval/favorable opinion of the appropriate IRB/IEC and, if legally required, competent authorities, except where necessary to eliminate an immediate hazard to the patients, or when the change(s) that were approved by NOXXON Pharma AG involve only logistical or administrative aspects of the study.

The study, and any amendments, will only be implemented following compliance with all legally required regulatory requirements.

14.4 Patient Information and Consent

Generally, interest in clinical studies for glioblastoma is high among patients and patient working groups. Interested patients will be seen for an initial interview and the general possibility of participation in the study is discussed based on the available reports, imaging studies and lab values. Local hospitals and practitioners will also be involved and send interested patients for initial interviews.

The Patient Information will be used to explain to the patient the risks and benefits of study participation.

The Consent Sheet will comply with all applicable regulations governing the protection of human patients, including ICH GCP guidelines, the Declaration of Helsinki, patient confidentiality and data protection. The IEC's written approval of the Patient Information and Consent Sheet will be obtained.

Prior to screening, patients will be provided with a copy of the approved Patient Information and Consent Sheet. The investigator or an authorized member of the study team will discuss with the patient the nature of the study and the treatments to be administered (including the risks and discomforts to be expected). Patients will have sufficient opportunity to inquire about details of the study, to discuss study related issues with their families and to decide whether or not to participate. They will be instructed that they are free to withdraw their participation in the study at any time and for any reason without prejudice.

The investigator will ensure that appropriate signatures and dates on the informed consent document are obtained prior to the performance of any protocol procedures and prior to the administration of study treatments. The investigator will provide each patient with a copy of the signed and dated consent and document the provision of consent in the patient's source notes.

The investigator will inform patients of new information that may be relevant to the patients' willingness to continue participation in the study and is also obligated to protect the patients' confidentiality. Any revision of the Patient Information and Consent Sheet must be approved by the IEC prior to use.

14.5 Confidentiality

The investigators, designated CRO and NOXXON Pharma AG and all other involved parties will preserve the confidentiality of all patients taking part in the study, in accordance with ICH GCP and local regulations. The confidentiality of all patient identities will be maintained, except during source data verification, when monitors, auditors and other authorized agents of the sponsor or its designee, the IECs approving this research, as well as any other applicable regulatory authorities will be granted direct access to the study patients' original medical records. No material bearing a patient’s name will be kept on file by designated CRO or NOXXON Pharma AG. The data retained from this study will be protected in accordance with all applicable legal requirements.

Information about study patients will be kept confidential and managed according to the EU Directive 2001/20/EC, 2005/28/EC, 2003/63/EC and EU Regulation No 536/2014 and relevant national and local legislations.

14.6 Finance, Patient Insurance Coverage and Investigator Indemnity

Financing agreements will be managed by the sponsor in a separate document.

NOXXON Pharma AG will provide appropriate insurance coverage for the patients, in accordance with legal requirements and local law. An insurance certificate will be provided for the investigator’s site file. Patients are to be informed by the investigator of the existence of

such insurance and that they have the right to inspect the terms and conditions of said insurance or may receive a copy, if required per national legislation.

14.7 Publication of Study Results

A final clinical study report will be prepared according to the ICH guideline on 'Structure and Content of Clinical Study Reports' (ICH E3). A final clinical study report will be prepared regardless of whether the study is completed or prematurely terminated. The clinical study report will be the sole property of the sponsor. Publication of the clinical study report or of parts of it may only be allowed when authorized by the sponsor. Reports to the authorities and IEC after study termination will be provided and results will be posted in the Clinical Study Register as required by law.

The investigator and the institution recognize that all data generated from this study are the proprietary and confidential information of the sponsor, along with all information supplied by the sponsor. The sponsor recognizes the Investigator's and the institution's rights and obligations, as an academic partner, to publish the study results. The study results will be published in accordance with the good publication practice guideline of the international society for medical publication professionals. This includes but is not limited to the following principles:

- The sponsor confirms the authors' freedom to make public or publish the study results and grants full access to the study data for this purpose. The investigator and the institution agree that they are not permitted to publish data related to the study independent of the sponsor and the other investigators.
- The investigator and the institution plan and produce publications in a timely manner and avoid premature release of study information.
- The sponsor and the Investigator and other individuals who have expertise in the area and who are willing to interpret the data and write or review articles and presentations will form a publication steering committee to oversee the preparation of articles and presentations from this study.

Prior to any written or oral presentation of the study results or any part thereof, the investigator or the institution shall send the full text of the proposed disclosure to the sponsor (or, if applicable, the publication steering committee) for review and comments at least 60 business days prior to submission for publication or oral presentation. The sponsor reserves the right to have deleted from the proposed publication any confidential information of the sponsor with the exception for results or data generated on the basis of the study which are of scientific interest to the investigator and which may be published in compliance with this section. During the above-mentioned period, sponsor in his sole discretion will take the steps he deems necessary to secure any intellectual property arising from the study results or data, including the filing or substantiating of one or more patent applications. The investigator or the institution shall follow customary principles related to scientific publications in determining and attributing authorship of any proposed publication, provided that any such publication shall acknowledge the sponsorship by the sponsor (65).

15. Amendments to the Protocol

15.1 Protocol Amendment 1

[redacted] [redacted]

[redacted]

[redacted] [redacted]

[redacted] [redacted]

[redacted]

[redacted] [redacted]

[redacted]

	[redacted]		[redacted]				[redacted]	
	[redacted]		[redacted]		[redacted]		[redacted]	[redacted]
[redacted]	[redacted]	[redacted]	[redacted]	[redacted]	[redacted]	[redacted]	[redacted]	[redacted]
[redacted]								
[redacted]	[redacted]			[redacted]	[redacted]	[redacted]	[redacted]	
[redacted]	[redacted]							

[redacted]

	[redacted]		[redacted]				[redacted]	
	[redacted]		[redacted]		[redacted]		[redacted]	[redacted]
[redacted]	[redacted]	[redacted]	[redacted]	[redacted]	[redacted]	[redacted]	[redacted]	[redacted]
[redacted]								
[redacted]	[redacted]			[redacted]	[redacted]	[redacted]	[redacted]	
[redacted]	[redacted]		[redacted]				[redacted]	
[redacted]	[redacted]		[redacted]				[redacted]	

[redacted]

- [redacted]
- [redacted]

[redacted]

- [redacted]
- [redacted]

16. Reference List

1. CBTRUS Statistical Report: Primary brain and other central nervous system tumors diagnosed in the United States in 2010–2014 | Neuro-Oncology | Oxford Academic (oup.com).
2. Abedalthagafi M, Barakeh D, Foshay KM. Immunogenetics of glioblastoma: the future of personalized patient management. NPJ Precis Oncol. 2018;2:27. Epub 2018/12/12. doi: 10.1038/s41698-018-0070-1. PubMed PMID: 30534602; PubMed Central PMCID: PMCPMC6279755.
3. Brown JM. Vasculogenesis: a crucial player in the resistance of solid tumours to radiotherapy. Br J Radiol. 2014;87(1035):20130686. Epub 2013/12/18. doi: 10.1259/bjr.20130686. PubMed PMID: 24338942; PubMed Central PMCID: PMCPMC4064599.
4. Kioi M, Vogel H, Schultz G, Hoffman RM, Harsh GR, Brown JM. Inhibition of vasculogenesis, but not angiogenesis, prevents the recurrence of glioblastoma after irradiation in mice. J Clin Invest. 2010;120(3):694-705. doi: 10.1172/JCI40283. PubMed PMID: 20179352; PubMed Central PMCID: PMC2827954.
5. Liu SC, Alomran R, Chernikova SB, Lartey F, Stafford J, Jang T, Merchant M, Zboralski D, Zollner S, Kruschinski A, Klussmann S, Recht L, Brown JM. Blockade of SDF-1 after irradiation inhibits tumor recurrences of autochthonous brain tumors in rats. Neuro-oncology. 2014;16(1):21-8. doi: 10.1093/neuonc/not149. PubMed PMID: 24335554; PubMed Central PMCID: PMC3870826.
6. Walters MJ, Ebsworth K, Berahovich RD, Penfold ME, Liu SC, Al Omran R, Kioi M, Chernikova SB, Tseng D, Mulkearns-Hubert EE, Sinyuk M, Ransohoff RM, Lathia JD, Karamchandani J, Kohrt HE, Zhang P, Powers JP, Jaen JC, Schall TJ, Merchant M, Recht L, Brown JM. Inhibition of CXCR7 extends survival following irradiation of brain tumours in mice and rats. Br J Cancer. 2014;110(5):1179-88. Epub 2014/01/16. doi: 10.1038/bjc.2013.830. PubMed PMID: 24423923; PubMed Central PMCID: PMCPMC3950859.
7. Chinot OL, Wick W, Mason W, Henriksson R, Saran F, Nishikawa R, Carpentier AF, Hoang-Xuan K, Kavan P, Cernea D, Brandes AA, Hilton M, Abrey L, Cloughesy T. Bevacizumab plus radiotherapy-temozolomide for newly diagnosed glioblastoma. N Engl J Med. 2014;370(8):709-22. Epub 2014/02/21. doi: 10.1056/NEJMoa1308345. PubMed PMID: 24552318.
8. Gilbert MR, Dignam JJ, Armstrong TS, Wefel JS, Blumenthal DT, Vogelbaum MA, Colman H, Chakravarti A, Pugh S, Won M, Jeraj R, Brown PD, Jaeckle KA, Schiff D, Stieber VW, Brachman DG, Werner-Wasik M, Tremont-Lukats IW, Sulman EP, Aldape KD, Curran WJ, Jr., Mehta MP. A randomized trial of bevacizumab for newly diagnosed glioblastoma. N Engl J Med. 2014;370(8):699-708. Epub 2014/02/21. doi: 10.1056/NEJMoa1308573. PubMed PMID: 24552317; PubMed Central PMCID: PMCPMC4201043.
9. de Groot JF, Lamborn KR, Chang SM, Gilbert MR, Cloughesy TF, Aldape K, Yao J, Jackson EF, Lieberman F, Robins HI, Mehta MP, Lassman AB, Deangelis LM, Yung WK, Chen A, Prados MD, Wen PY. Phase II study of aflibercept in recurrent malignant glioma: a North American Brain Tumor Consortium study. J Clin Oncol. 2011;29(19):2689-95. doi: 10.1200/JCO.2010.34.1636. PubMed PMID: 21606416; PubMed Central PMCID: PMCPMC3139373.
10. Jin F, Hagemann N, Schäfer ST, Brockmeier U, Zechariah A, Hermann DM. SDF-1 restores angiogenesis synergistically with VEGF upon LDL exposure despite CXCR4 internalization and degradation. Cardiovasc Res. 2013;100(3):481-91. Epub 2013/09/10. doi: 10.1093/cvr/cvt209. PubMed PMID: 24014104.
11. Jin DK, Shido K, Kopp HG, Petit I, Shmelkov SV, Young LM, Hooper AT, Amano H, Avecilla ST, Heissig B, Hattori K, Zhang F, Hicklin DJ, Wu Y, Zhu Z, Dunn A, Salari H, Werb Z, Hackett NR, Crystal RG, Lyden D, Rafii S. Cytokine-mediated deployment of SDF-1 induces revascularization through recruitment of CXCR4+ hemangiocytes. Nat Med. 2006;12(5):557-67. PubMed PMID: 16648859.
12. Ruiz de Almodovar C, Luttun A, Carmeliet P. An SDF-1 Trap for Myeloid Cells Stimulates Angiogenesis. Cell. 2006;124(1):18-21. PubMed PMID: 16413476.

13. Yu JX, Huang XF, Lv WM, Ye CS, Peng XZ, Zhang H, Xiao LB, Wang SM. Combination of stromal-derived factor-1alpha and vascular endothelial growth factor gene-modified endothelial progenitor cells is more effective for ischemic neovascularization. J Vasc Surg. 2009;50(3):608-16. Epub 2009/07/15. doi: 10.1016/j.jvs.2009.05.049. PubMed PMID: 19595531.

14. Hiasa K, Ishibashi M, Ohtani K, Inoue S, Zhao Q, Kitamoto S, Sata M, Ichiki T, Takeshita A, Egashira K. Gene transfer of stromal cell-derived factor-1alpha enhances ischemic vasculogenesis and angiogenesis via vascular endothelial growth factor/endothelial nitric oxide synthase-related pathway: next-generation chemokine therapy for therapeutic neovascularization. Circulation. 2004;109(20):2454-61. Epub 2004/05/19. doi: 10.1161/01.Cir.0000128213.96779.61. PubMed PMID: 15148275.

15. Ho TK, Tsui J, Xu S, Leoni P, Abraham DJ, Baker DM. Angiogenic effects of stromal cell-derived factor-1 (SDF-1/CXCL12) variants in vitro and the in vivo expressions of CXCL12 variants and CXCR4 in human critical leg ischemia. J Vasc Surg. 2010;51(3):689-99. Epub 2010/03/09. doi: 10.1016/j.jvs.2009.10.044. PubMed PMID: 20206813.

16. Salcedo R, Oppenheim JJ. Role of chemokines in angiogenesis: CXCL12/SDF-1 and CXCR4 interaction, a key regulator of endothelial cell responses. Microcirculation. 2003;10(3-4):359-70. PubMed PMID: 12851652.

17. Kryczek I, Lange A, Mottram P, Alvarez X, Cheng P, Hogan M, Moons L, Wei S, Zou L, Machelon V, Emilie D, Terrassa M, Lackner A, Curiel TJ, Carmeliet P, Zou W. CXCL12 and vascular endothelial growth factor synergistically induce neoangiogenesis in human ovarian cancers. Cancer Res. 2005;65(2):465-72. PubMed PMID: 15695388.

18. Reddy K, Zhou Z, Jia SF, Lee TH, Morales-Arias J, Cao Y, Kleinerman ES. Stromal cell-derived factor-1 stimulates vasculogenesis and enhances Ewing's sarcoma tumor growth in the absence of vascular endothelial growth factor. Int J Cancer. 2008;123(4):831-7. Epub 2008/06/10. doi: 10.1002/ijc.23582. PubMed PMID: 18537159; PubMed Central PMCID: PMCPMC2587257.

19. Aghi M, Cohen KS, Klein RJ, Scadden DT, Chiocca EA. Tumor stromal-derived factor-1 recruits vascular progenitors to mitotic neovasculature, where microenvironment influences their differentiated phenotypes. Cancer Res. 2006;66(18):9054-64. Epub 2006/09/20. doi: 10.1158/0008-5472.Can-05-3759. PubMed PMID: 16982747.

20. Guleng B, Tateishi K, Ohta M, Kanai F, Jazag A, Ijichi H, Tanaka Y, Washida M, Morikane K, Fukushima Y, Yamori T, Tsuruo T, Kawabe T, Miyagishi M, Taira K, Sata M, Omata M. Blockade of the stromal cell-derived factor-1/CXCR4 axis attenuates in vivo tumor growth by inhibiting angiogenesis in a vascular endothelial growth factor-independent manner. Cancer Res. 2005;65(13):5864-71. PubMed PMID: 15994964.

21. Song CW, Park I, Cho LC, Yuan J, Dusenbery KE, Griffin RJ, Levitt SH. Is indirect cell death involved in response of tumors to stereotactic radiosurgery and stereotactic body radiation therapy? International journal of radiation oncology, biology, physics. 2014;89(4):924-5. Epub 2014/06/28. doi: 10.1016/j.ijrobp.2014.03.043. PubMed PMID: 24969800.

22. Han ZB, Ren H, Zhao H, Chi Y, Chen K, Zhou B, Liu YJ, Zhang L, Xu B, Liu B, Yang R, Han ZC. Hypoxia-inducible factor (HIF)-1 alpha directly enhances the transcriptional activity of stem cell factor (SCF) in response to hypoxia and epidermal growth factor (EGF). Carcinogenesis. 2008;29(10):1853-61. Epub 2008/03/15. doi: 10.1093/carcin/bgn066. PubMed PMID: 18339685.

23. Kelly BD, Hackett SF, Hirota K, Oshima Y, Cai Z, Berg-Dixon S, Rowan A, Yan Z, Campochiaro PA, Semenza GL. Cell type-specific regulation of angiogenic growth factor gene expression and induction of angiogenesis in nonischemic tissue by a constitutively active form of hypoxia-inducible factor 1. Circ Res. 2003;93(11):1074-81. Epub 2003/10/25. doi: 10.1161/01.Res.0000102937.50486.1b. PubMed PMID: 14576200.

24. Greenfield JP, Cobb WS, Lyden D. Resisting arrest: a switch from angiogenesis to vasculogenesis in recurrent malignant gliomas. J Clin Invest. 2010;120(3):663-7. Epub 2010/02/25. doi: 10.1172/jci42345. PubMed PMID: 20179347; PubMed Central PMCID: PMCPMC2827970.

25. Hambardzumyan D, Bergers G. Glioblastoma: Defining Tumor Niches. Trends in cancer. 2015;1(4):252-65. Epub 2016/04/19. doi: 10.1016/j.trecan.2015.10.009. PubMed PMID: 27088132; PubMed Central PMCID: PMCPMC4831073.

26. Puchalski RB, Shah N, Miller J, Dalley R, Nomura SR, Yoon JG, Smith KA, Lankerovich M, Bertagnolli D, Bickley K, Boe AF, Brouner K, Butler S, Caldejon S, Chapin M, Datta S, Dee N, Desta T, Dolbeare T, Dotson N, Ebbert A, Feng D, Feng X, Fisher M, Gee G, Goldy J, Gourley L, Gregor BW, Gu G, Hejazinia N, Hohmann J, Hothi P, Howard R, Joines K, Kriedberg A, Kuan L, Lau C, Lee F, Lee H, Lemon T, Long F, Mastan N, Mott E, Murthy C, Ngo K, Olson E, Reding M, Riley Z, Rosen D, Sandman D, Shapovalova N, Slaughterbeck CR, Sodt A, Stockdale G, Szafer A, Wakeman W, Wohnoutka PE, White SJ, Marsh D, Rostomily RC, Ng L, Dang C, Jones A, Keogh B, Gittleman HR, Barnholtz-Sloan JS, Cimino PJ, Uppin MS, Keene CD, Farrokhi FR, Lathia JD, Berens ME, Iavarone A, Bernard A, Lein E, Phillips JW, Rostad SW, Cobbs C, Hawrylycz MJ, Foltz GD. An anatomic transcriptional atlas of human glioblastoma. Science. 2018;360(6389):660-3. Epub 2018/05/12. doi: 10.1126/science.aaf2666. PubMed PMID: 29748285; PubMed Central PMCID: PMCPMC6414061.

27. Deng L, Stafford JH, Liu SC, Chernikova SB, Merchant M, Recht L, Martin Brown J. SDF-1 Blockade Enhances Anti-VEGF Therapy of Glioblastoma and Can Be Monitored by MRI. Neoplasia. 2017;19(1):1-7. Epub 2016/12/13. doi: 10.1016/j.neo.2016.11.010. PubMed PMID: 27940247; PubMed Central PMCID: PMCPMC5149063.

28. Bristol-Myers Squibb Announces Phase 3 CheckMate -498 Study Did Not Meet Primary Endpoint of Overall Survival with Opdivo (nivolumab) Plus Radiation in Patients with Newly Diagnosed MGMT-Unmethylated Glioblastoma Multiforme [Internet]. 2019. Available from: https://news.bms.com/news/corporate-financial/2019/Bristol-Myers-Squibb-Announces-Phase-3-CheckMate--498-Study-Did-Not-Meet-Primary-Endpoint-of-Overall-Survival-with-Opdivo-nivolumab-Plus-Radiation-in-Patients-with-Newly-Diagnosed-MGMT-Unmethylated-Glioblastoma-Multiforme/default.aspx

29. Reardon DA, Brandes AA, Omuro A, Mulholland P, Lim M, Wick A, Baehring J, Ahluwalia MS, Roth P, Bähr O, Phuphanich S, Sepulveda JM, De Souza P, Sahebjam S, Carleton M, Tatsuoka K, Taitt C, Zwirtes R, Sampson J, Weller M. Effect of Nivolumab vs Bevacizumab in Patients With Recurrent Glioblastoma: The CheckMate 143 Phase 3 Randomized Clinical Trial. JAMA Oncol. 2020;6(7):1003-10. Epub 2020/05/22. doi: 10.1001/jamaoncol.2020.1024. PubMed PMID: 32437507.

30. Tomaszewski W, Sanchez-Perez L, Gajewski TF, Sampson JH. Brain Tumor Microenvironment and Host State: Implications for Immunotherapy. Clin Cancer Res. 2019;25(14):4202-10. Epub 2019/02/26. doi: 10.1158/1078-0432.Ccr-18-1627. PubMed PMID: 30804019; PubMed Central PMCID: PMCPMC6635001.

31. Wu A, Maxwell R, Xia Y, Cardarelli P, Oyasu M, Belcaid Z, Kim E, Hung A, Luksik AS, Garzon-Muvdi T, Jackson CM, Mathios D, Theodros D, Cogswell J, Brem H, Pardoll DM, Lim M. Combination anti-CXCR4 and anti-PD-1 immunotherapy provides survival benefit in glioblastoma through immune cell modulation of tumor microenvironment. J Neurooncol. 2019;143(2):241-9. Epub 2019/04/27. doi: 10.1007/s11060-019-03172-5. PubMed PMID: 31025274.

32. Ludwig H, Kasparu H, Leitgeb C, Rauch E, Linkesch W, Zojer N, Greil R, Seebacher A, Pour L, Weißmann A, Adam Z. Bendamustine-bortezomib-dexamethasone is an active and well-tolerated regimen in patients with relapsed or refractory multiple myeloma. Blood. 2014;123(7):985-91. Epub 2013/11/15. doi: 10.1182/blood-2013-08-521468. PubMed PMID: 24227817; PubMed Central PMCID: PMCPMC3924931.

33. Steurer M, Montillo M, Scarfò L, Mauro FR, Andel J, Wildner S, Trentin L, Janssens A, Burgstaller S, Frömming A, Dümmler T, Riecke K, Baumann M, Beyer D, Vauléon S, Ghia P, Foà R, Caligaris-Cappio F, Gobbi M. Olaptesed pegol (NOX-A12) with bendamustine and rituximab: a phase IIa study in patients with relapsed/refractory chronic lymphocytic leukemia. Haematologica. 2019;104(10):2053-60. Epub 2019/05/18. doi: 10.3324/haematol.2018.205930. PubMed PMID: 31097627; PubMed Central PMCID: PMCPMC6886437.

34. Halama N. Poster presentation: Halama N, Prüfer U, Frömming A, Beyer D, Eulberg D, Jungnelius JU, Mangasarian A, Evaluation of tumor biomarkers in patients with microsatellite-stable, metastatic colorectal or pancreatic cancer treated with the CXCL12 inhibitor NOX-A12 and preliminary safety in combination with PD-1 checkpoint inhibitor pembrolizumab, 4th CRI-CIMT-EATI-AACR International Cancer Immunotherapy Conference, 2018.

35. Stupp R, Hegi ME, Mason WP, van den Bent MJ, Taphoorn MJ, Janzer RC, Ludwin SK, Allgeier A, Fisher B, Belanger K, Hau P, Brandes AA, Gijtenbeek J, Marosi C, Vecht CJ, Mokhtari K, Wesseling P, Villa S, Eisenhauer E, Gorlia T, Weller M, Lacombe D, Cairncross JG, Mirimanoff RO, European Organisation for R, Treatment of Cancer Brain T, Radiation Oncology G, National Cancer Institute of Canada Clinical Trials G. Effects of radiotherapy with concomitant and adjuvant temozolomide versus radiotherapy alone on survival in glioblastoma in a randomised phase III study: 5-year analysis of the EORTC-NCIC trial. Lancet Oncol. 2009;10(5):459-66. Epub 2009/03/10. doi: 10.1016/S1470-2045(09)70025-7. PubMed PMID: 19269895.

36. Stummer W, Meinel T, Ewelt C, Martus P, Jakobs O, Felsberg J, Reifenberger G. Prospective cohort study of radiotherapy with concomitant and adjuvant temozolomide chemotherapy for glioblastoma patients with no or minimal residual enhancing tumor load after surgery. J Neurooncol. 2012;108(1):89-97. Epub 2012/02/07. doi: 10.1007/s11060-012-0798-3. PubMed PMID: 22307805; PubMed Central PMCID: PMCPMC3337400.

37. Ellingson BM, Wen PY, Cloughesy TF. Modified Criteria for Radiographic Response Assessment in Glioblastoma Clinical Trials. Neurotherapeutics. 2017;14(2):307-20. Epub 2017/01/22. doi: 10.1007/s13311-016-0507-6. PubMed PMID: 28108885; PubMed Central PMCID: PMCPMC5398984.

38. Nayak L, DeAngelis LM, Brandes AA, Peereboom DM, Galanis E, Lin NU, Soffietti R, Macdonald DR, Chamberlain M, Perry J, Jaeckle K, Mehta M, Stupp R, Muzikansky A, Pentsova E, Cloughesy T, Iwamoto FM, Tonn JC, Vogelbaum MA, Wen PY, van den Bent MJ, Reardon DA. The Neurologic Assessment in Neuro-Oncology (NANO) scale: a tool to assess neurologic function for integration into the Response Assessment in Neuro-Oncology (RANO) criteria. Neuro-oncology. 2017;19(5):625-35. Epub 2017/04/30. doi: 10.1093/neuonc/nox029. PubMed PMID: 28453751; PubMed Central PMCID: PMCPMC5464449.

39. Le Tourneau C, Lee JJ, Siu LL. Dose escalation methods in phase I cancer clinical trials. J Natl Cancer Inst. 2009;101(10):708-20. Epub 2009/05/14. doi: 10.1093/jnci/djp079. PubMed PMID: 19436029; PubMed Central PMCID: PMCPMC2684552.

40. Hattermann K, Held-Feindt J, Lucius R, Müerköster SS, Penfold ME, Schall TJ, Mentlein R. The chemokine receptor CXCR7 is highly expressed in human glioma cells and mediates antiapoptotic effects. Cancer Res. 2010;70(8):3299-308. Epub 2010/04/15. doi: 10.1158/0008-5472.Can-09-3642. PubMed PMID: 20388803.

41. Maderna E, Salmaggi A, Calatozzolo C, Limido L, Pollo B. Nestin, PDGFRbeta, CXCL12 and VEGF in glioma patients: different profiles of (pro-angiogenic) molecule expression are related with tumor grade and may provide prognostic information. Cancer biology & therapy. 2007;6(7):1018-24. Epub 2007/07/06. doi: 10.4161/cbt.6.7.4362. PubMed PMID: 17611402.

42. De Palma M, Lewis CE. Macrophage regulation of tumor responses to anticancer therapies. Cancer Cell. 2013;23(3):277-86. Epub 2013/03/23. doi: 10.1016/j.ccr.2013.02.013. PubMed PMID: 23518347.

43. Dalton HJ, Pradeep S, McGuire M, Hailemichael Y, Ma S, Lyons Y, Armaiz-Pena GN, Previs RA, Hansen JM, Rupaimoole R, Gonzalez-Villasana V, Cho MS, Wu SY, Mangala LS, Jennings NB, Hu W, Langley R, Mu H, Andreeff M, Bar-Eli M, Overwijk W, Ram P, Lopez-Berestein G, Coleman RL, Sood AK. Macrophages Facilitate Resistance to Anti-VEGF Therapy by Altered VEGFR Expression. Clin Cancer Res. 2017;23(22):7034-46. Epub 2017/09/01. doi: 10.1158/1078-0432.Ccr-17-0647. PubMed PMID: 28855350; PubMed Central PMCID: PMCPMC5690831.

44. Zboralski D, Hoehlig K, Eulberg D, Fromming A, Vater A. Increasing Tumor-Infiltrating T Cells through Inhibition of CXCL12 with NOX-A12 Synergizes with PD-1 Blockade. Cancer immunology research. 2017;5(11):950-6. doi: 10.1158/2326-6066.CIR-16-0303. PubMed PMID: 28963140.

45. Feig C, Jones JO, Kraman M, Wells RJ, Deonarine A, Chan DS, Connell CM, Roberts EW, Zhao Q, Caballero OL, Teichmann SA, Janowitz T, Jodrell DI, Tuveson DA, Fearon DT. Targeting CXCL12 from FAP-expressing carcinoma-associated fibroblasts synergizes with anti-PD-L1 immunotherapy in pancreatic cancer. Proc Natl Acad Sci U S A. 2013;110(50):20212-7. doi: 10.1073/pnas.1320318110. PubMed PMID: 24277834; PubMed Central PMCID: PMC3864274.

46. D'Alterio C, Buoncervello M, Ieranò C, Napolitano M, Portella L, Rea G, Barbieri A, Luciano A, Scognamiglio G, Tatangelo F, Anniciello AM, Monaco M, Cavalcanti E, Maiolino P, Romagnoli G, Arra C, Botti G, Gabriele L, Scala S. Targeting CXCR4 potentiates anti-PD-1 efficacy modifying the tumor microenvironment and inhibiting neoplastic PD-1. Journal of experimental & clinical cancer research : CR. 2019;38(1):432. Epub 2019/10/30. doi: 10.1186/s13046-019-1420-8. PubMed PMID: 31661001; PubMed Central PMCID: PMCPMC6819555.

47. NOXXON Clinical Study Report SNOXA12C001 “NOX-A12 to mobilize stem cells in healthy volunteers - A single center, open label, dose escalation study of intravenous NOX-A12 in up to 48 healthy subjects” Version 1.0, 18 Apr 2011.

48. NOXXON Clinical Study Report SNOXA12C101 “A single center, open-label, repeated dose, Phase I study in healthy subjects to assess the safety, tolerability, pharmacokinetics and the effect on mobilization of hematopoietic stem cells of NOX-A12 alone and in combination with filgrastim.” Version 1.0, 13 Sep 2011.

49. NOXXON Clinical Study Report SNOXA12C201 “A multi-center, open label, uncontrolled, Phase IIa clinical trial evaluating the safety and efficacy of Olaptesed Pegol in combination with a background therapy of bendamustine and rituximab (BR) in previously treated patients with chronic lymphocytic leukemia”, Version 2.0, 09 Oct 2017.

50. NOXXON Clinical Study Report SNOXA12C301 “A multi-center, open label, uncontrolled, Phase IIa clinical trial evaluating the safety and efficacy of NOX-A12 in combination with a background therapy of bortezomib and dexamethasone in previously treated patients with multiple myeloma”, Version 1.0, 29 Jun 2016.

51. Poster presentation: "Final Top-Line Data: CXCL12 inhibitor NOX-A12 and pembrolizumab in microsatellite-stable, metastatic colorectal or pancreatic cancer", Niels Halama, Anja Williams, Meggy Suarez-Carmona, Jutta Schreiber, Nicolas Hohmann, Ulrike Pruefer, Jürgen Krauss, Dirk Jaeger, Anna Frömming, Diana Beyer, Dirk Eulberg, Jarl Ulf Jungnelius, Aram Mangasarian, ESMO 2020.

52. Herrlinger U, Schäfer N, Steinbach JP, Weyerbrock A, Hau P, Goldbrunner R, Friedrich F, Rohde V, Ringel F, Schlegel U, Sabel M, Ronellenfitsch MW, Uhl M, Maciaczyk J, Grau S, Schnell O, Hänel M, Krex D, Vajkoczy P, Gerlach R, Kortmann RD, Mehdorn M, Tüttenberg J, Mayer-Steinacker R, Fietkau R, Brehmer S, Mack F, Stuplich M, Kebir S, Kohnen R, Dunkl E, Leutgeb B, Proescholdt M, Pietsch T, Urbach H, Belka C, Stummer W, Glas M. Bevacizumab Plus Irinotecan Versus Temozolomide in Newly Diagnosed O6-Methylguanine-DNA Methyltransferase Nonmethylated Glioblastoma: The Randomized GLARIUS Trial. J Clin Oncol. 2016;34(14):1611-9. Epub 2016/03/16. doi: 10.1200/jco.2015.63.4691. PubMed PMID: 26976423.

53. Wick W, Gorlia T, Bady P, Platten M, van den Bent MJ, Taphoorn MJ, Steuve J, Brandes AA, Hamou MF, Wick A, Kosch M, Weller M, Stupp R, Roth P, Golfinopoulos V, Frenel JS, Campone M, Ricard D, Marosi C, Villa S, Weyerbrock A, Hopkins K, Homicsko K, Lhermitte B, Pesce G, Hegi ME. Phase II Study of Radiotherapy and Temsirolimus versus Radiochemotherapy with Temozolomide in Patients with Newly Diagnosed Glioblastoma without MGMT Promoter Hypermethylation (EORTC 26082). Clin Cancer Res. 2016;22(19):4797-806. Epub 2016/05/05. doi: 10.1158/1078-0432.Ccr-15-3153. PubMed PMID: 27143690.

54. Reardon DA, Kim TM, Frenel JS, Simonelli M, Lopez J, Subramaniam DS, Siu LL, Wang H, Krishnan S, Stein K, Massard C. Treatment with pembrolizumab in programmed death ligand 1-positive recurrent glioblastoma: Results from the multicohort phase 1 KEYNOTE-028 trial. Cancer. 2021;127(10):1620-9. Epub 2021/01/27. doi: 10.1002/cncr.33378. PubMed PMID: 33496357.

55. Marabelle A, Le DT, Ascierto PA, Di Giacomo AM, De Jesus-Acosta A, Delord JP, Geva R, Gottfried M, Penel N, Hansen AR, Piha-Paul SA, Doi T, Gao B, Chung HC, Lopez-Martin J, Bang YJ, Frommer RS, Shah M, Ghori R, Joe AK, Pruitt SK, Diaz LA, Jr. Efficacy of Pembrolizumab in

Patients With Noncolorectal High Microsatellite Instability/Mismatch Repair-Deficient Cancer: Results From the Phase II KEYNOTE-158 Study. J Clin Oncol. 2020;38(1):1-10. Epub 2019/11/05. doi: 10.1200/jco.19.02105. PubMed PMID: 31682550.

56. Bristol Myers Squibb Announces Update on Phase 3 CheckMate -548 Trial Evaluating Patients with Newly Diagnosed MGMT-Methylated Glioblastoma Multiforme [Internet]. 2020. Available from: https://news.bms.com/news/details/2020/Bristol-Myers-Squibb-Announces-Update-on-Phase-3-CheckMate--548-Trial-Evaluating-Patients-with-Newly-Diagnosed-MGMT-Methylated-Glioblastoma-Multiforme/default.aspx

57. Thomas RP et al. Poster presentation ESNO 2016 "A Phase I study of chemo-radiotherapy with plerixafor for newly diagnosed Glioblastoma (GB)".

58. Jones LW, Ali-Osman F, Lipp E, Marcello JE, McCarthy B, McCoy L, Rice T, Wrensch M, Il'yasova D. Association between body mass index and mortality in patients with glioblastoma mutliforme. Cancer causes & control : CCC. 2010;21(12):2195-201. Epub 2010/09/15. doi: 10.1007/s10552-010-9639-x. PubMed PMID: 20838873; PubMed Central PMCID: PMCPMC3312598.

59. NOXXON Study Report SNOXA12C106, „Quantification of SDF-1 in Human Plasma Samples: Non-regulatory Analysis of Samples from Phase I Clinical Study SNOXA12C101", 08 November 2012.

60. SNOXA12C401 - Vote DSMB meeting 28-April-2021.

61. Lai A, Tran A, Nghiemphu PL, Pope WB, Solis OE, Selch M, Filka E, Yong WH, Mischel PS, Liau LM, Phuphanich S, Black K, Peak S, Green RM, Spier CE, Kolevska T, Polikoff J, Fehrenbacher L, Elashoff R, Cloughesy T. Phase II study of bevacizumab plus temozolomide during and after radiation therapy for patients with newly diagnosed glioblastoma multiforme. J Clin Oncol. 2011;29(2):142-8. Epub 2010/12/08. doi: 10.1200/jco.2010.30.2729. PubMed PMID: 21135282; PubMed Central PMCID: PMCPMC3058273 found at the end of this article.

62. Vredenburgh JJ, Desjardins A, Reardon DA, Peters KB, Herndon JE, 2nd, Marcello J, Kirkpatrick JP, Sampson JH, Bailey L, Threatt S, Friedman AH, Bigner DD, Friedman HS. The addition of bevacizumab to standard radiation therapy and temozolomide followed by bevacizumab, temozolomide, and irinotecan for newly diagnosed glioblastoma. Clin Cancer Res. 2011;17(12):4119-24. Epub 2011/05/03. doi: 10.1158/1078-0432.Ccr-11-0120. PubMed PMID: 21531816; PubMed Central PMCID: PMCPMC3117928.

63. International Conference on Harmonisation. ICH Harmonised Tripartite Guideline. Good Clinical Practice E6(R2), 14-Jun-2017.

64. World Medical Association. Declaration of Helsinki.Ethical Principles for Medical Research Involving Human Subjects. 59th WMA General Assembly, Seoul, Republic of Korea, October 2008.

65. Battisti WP, Wager E, Baltzer L, Bridges D, Cairns A, Carswell CI, Citrome L, Gurr JA, Mooney LA, Moore BJ, Peña T, Sanes-Miller CH, Veitch K, Woolley KL, Yarker YE. Good Publication Practice for Communicating Company-Sponsored Medical Research: GPP3. Ann Intern Med. 2015;163(6):461-4. Epub 2015/08/11. doi: 10.7326/m15-0288. PubMed PMID: 26259067.

66. Deng L, Zheng W, Dong X, Liu J, Zhu C, Lu D, Zhang J, Song L, Wang Y, Deng D. Chemokine receptor CXCR7 is an independent prognostic biomarker in glioblastoma. Cancer biomarkers : section A of Disease markers. 2017;20(1):1-6. doi: 10.3233/CBM-151430. PubMed PMID: 28759950.

Supplementary Table 1: Complete listing of treatment-emergent adverse events related to NOX-A12 and/or underlying disease and/or irradiation per dose level.

**System Organ Class** Preferred Term	**Dose level 1 200 mg/week NOX-A12**		**Dose level 2 400 mg/week NOX-A12**		**Dose level 3 600 mg/week NOX-A12**	
	# of events	# of patients	# of events	# of patients	# of events	# of patients
**Nervous system disorders**	**5**	**2**				
Paralysis	1	1				
Headache	1	1				
Partial seizures	1	1				
Monoparesis	1	1				
Paraesthesia	1	1				
**Investigations**	**1**	**1**			**4**	**2**
Alanine aminotransferase increased	1	1			2	2
Gamma-glutamyltransferase increased					2	2
**Gastrointestinal disorders**	**1**	**1**			**3**	**1**
Constipation					3	1
Nausea	1	1				
**Blood and lymphatic system disorders**			**1**	**1**	**2**	**1**
Leukocytosis			1	1	2	1
**General disorders and administration site conditions**	**2**	**2**				
Pyrexia	1	1				
Fatigue	1	1				
**Ear and labyrinth disorders**	**1**	**1**				
Vertigo	1	1				
**Respiratory, thoracic and mediastinal disorders**	**1**	**1**				
Dyspnoea exertional	1	1				
**Psychiatric disorders**	**1**	**1**				
Logorrhoea	1	1				
**Total**	**12**	**3**	**1**	**1**	**9**	**2**

Supplementary Table 2: Complete listing of treatment-emergent adverse events related to NOX-A12 only per dose level.

System Organ Class<br>Preferred Term	Dose level 1<br>200 mg/week NOX-A12		Dose level 2<br>400 mg/week NOX-A12		Dose level 3<br>600 mg/week NOX-A12	
	# of events	# of patients	# of events	# of patients	# of events	# of patients
**Nervous system disorders**	**1**	**1**				
Paraesthesia	1	1				
**Investigations**	**1**	**1**			**4**	**2**
Alanine aminotransferase increased	1	1			2	2
Gamma-glutamyltransferase increased					2	2
**Gastrointestinal disorders**	**1**	**1**			**3**	**1**
Constipation					3	1
**Blood and lymphatic system disorders**			**1**	**1**	**2**	**1**
Leukocytosis			1	1	2	1
**General disorders and administration site conditions**	**2**	**2**				
Pyrexia	1	1				
**Respiratory, thoracic and mediastinal disorders**	**1**	**1**				
Dyspnoea exertional	1	1				
**Total**	**4**	**3**	**1**	**1**	**9**	**2**

Supplementary Table 3: Patient characteristics of the SOC cohort (n=22). ECOG: Eastern Cooperative Oncology Group; IDH: isocitrate dehydrogenase; MGMT: O^6-methylguanine DNA methyltransferase; MRI: magnetic resonance imaging; n: number; SOC: standard-of-care.

**Variable**	**n (%)**	**Median (range)**
Gender		
Male	15 (68.2)	
Female	7 (31.8)	
Age (years)		60 (44-82)
ECOG Score		
0	12 (54.5)	
1	5 (22.7)	
2	5 (22.7)	
Methylation status		
Unmethylated	22 (100.0)	
IDH status		
Wildtype	22 (100.0)	
Resection status		
Incomplete Resection	15 (68.2)	
Biopsy	7 (31.8)	
Radiotherapy		
Normofractionated	18 (81.8)	
Hypofractionated	4 (18.2)	
First line systemic therapy		
Temozolomide	18 (81.8)	
None	4 (18.2)	
Consecutive MRI scans		4.5 (3-13)

Supplementary Table 4: Patient stratification by the combined EG12 score compared to isolated E12 and G12 assessment. Table showing PFS, OS, E12 (%), G12 (%) and EG12 score of GLORIA patients sorted by EG12 score from high to low. E12: CXCL12⁺ endothelial cells; G12: CXCL12⁺ glioma cells; OS: overall survival; PFS: progression-free survival.

Patient	PFS (days)	OS (days)	E12 (%)	G12 (%)	EG12 Score
C3-001	183	350	77.45	24,20	17.78
C1-001	260	545	72.12	8,96	7.49
C2-001	120	287	55.28	21,60	5.39
C1-003	183	562	56.97	19,14	5.01
C3-003	174	481	51.84	23,06	4.40
C2-003	120	427	64.88	3,28	1.03
C3-004	183	338	50.52	14,73	-0.42
C1-002	92	150	53.23	8,58	-2.14
C3-002	71	144	48.00	2,19	-7.96
C2-002	58	440	38.46	2.88	-12.38
Median			54.25	11.85	2.72

> Median
< Median
