# Supplementary material for: L-RNA aptamer-based CXCL12 inhibition combined with radiotherapy in newly-diagnosed glioblastoma: dose escalation of the phase I/II GLORIA trial
Source: Nat Commun. 2024 May 28;15:4210. doi: 10.1038/s41467-024-48416-9 (PMC11133480; doi:10.1038/s41467-024-48416-9)
Supplement: Supplementary file 2 — Reporting Summary [file 41467_2024_48416_MOESM2_ESM.pdf]

nature portfolio

Corresponding author(s): Michael Hölzel

Last updated by author(s): Apr 28, 2024

Reporting Summary

Nature Portfolio wishes to improve the reproducibility of the work that we publish. This form provides structure for consistency and transparency in reporting. For further information on Nature Portfolio policies, see our Editorial Policies and the Editorial Policy Checklist.

Statistics

For all statistical analyses, confirm that the following items are present in the figure legend, table legend, main text, or Methods section.

n/a	Confirmed	
☐	☒	The exact sample size (*n*) for each experimental group/condition, given as a discrete number and unit of measurement
☒	☐	A statement on whether measurements were taken from distinct samples or whether the same sample was measured repeatedly
☐	☒	The statistical test(s) used AND whether they are one- or two-sided<br>*Only common tests should be described solely by name; describe more complex techniques in the Methods section.*
☒	☐	A description of all covariates tested
☒	☐	A description of any assumptions or corrections, such as tests of normality and adjustment for multiple comparisons
☐	☒	A full description of the statistical parameters including central tendency (e.g. means) or other basic estimates (e.g. regression coefficient) AND variation (e.g. standard deviation) or associated estimates of uncertainty (e.g. confidence intervals)
☐	☒	For null hypothesis testing, the test statistic (e.g. *F*, *t*, *r*) with confidence intervals, effect sizes, degrees of freedom and *P* value noted<br>*Give P values as exact values whenever suitable.*
☒	☐	For Bayesian analysis, information on the choice of priors and Markov chain Monte Carlo settings
☒	☐	For hierarchical and complex designs, identification of the appropriate level for tests and full reporting of outcomes
☒	☐	Estimates of effect sizes (e.g. Cohen's *d*, Pearson's *r*), indicating how they were calculated

Our web collection on statistics for biologists contains articles on many of the points above.

Software and code

Policy information about availability of computer code

Data collection	Graphical elements were generated using GraphPad Prism 10 (GraphPad Software) and Adobe Illustrator 2023 (Adobe Inc.). Database management (eCRF) was carried out using Viedoc version 4.66 eCRF (Viedoc Technologies) and Microsoft Excel 2019 (Microsoft Corporation). For mIF, Imaging cycles were performed using an Akoya Phenocycler" instrument and CODEX® instrument manager software (Akoya Biosciences).
Data analysis	mIF images were processed with CODEX® Processor (Akoya Biosciences) and analyzed with HALO® Image Analysis software (Indica Labs, v.3.3). Statistical tests were performed using GraphPad Prism 9 and R (V.3.3.2, x86_64-pc-linux-gnu). Dataset GSE18210935 was downloaded from the Broad Institute Single Cell Portal (https://singlecell.broadinstitute.org/single_cell). Data was analyzed and visualized using Rversion 4.2.2. CXCL12 expression was overlayed on UMAP using scCustomize (Version 1.1.1) using the FeaturePlot_scCustom() function with RColorBrewer (Version 1.1-3) and the color pallet "OrRd". Cells were called positive for CXCL12 if they had a log-normalized count of 1 or more. All code used to analyze and plot scRNAseq data can be found under the relevant repository at https://github.com/BaldLab

For manuscripts utilizing custom algorithms or software that are central to the research but not yet described in published literature, software must be made available to editors and reviewers. We strongly encourage code deposition in a community repository (e.g. GitHub). See the Nature Portfolio guidelines for submitting code & software for further information.

Data

Policy information about availability of data

All manuscripts must include a data availability statement. This statement should provide the following information, where applicable:

- Accession codes, unique identifiers, or web links for publicly available datasets
- A description of any restrictions on data availability
- For clinical datasets or third party data, please ensure that the statement adheres to our policy

The study protocol is made available as Supplementary Note 2. Source data are provided with this paper. The data generated in this study are provided in the Supplementary Information and Source Data file. High resolution images of Supplementary Fig. 6 are provided in the following repository: "Giordano, Layer, Leonardelli et al. Supplementary Fig. 6", Mendeley Data, V1, doi: 10.17632/wfhnv7j2wh.1 (https://data.mendeley.com/datasets/wfhnv7j2wh/1). The publicly available scRNAseq data set used for re-analysis (from Abdelfattah et al.)(ref. 35) can be accessed via the GEO archive provided under accession ID GSE182109. Identifying individual participant data are protected and are not available due to data privacy laws. Individual de-identified participant data are available upon written request from the sponsor (according to local legal requirements for at least ten years).

All code generated in this study to analyze and plot scRNAseq data have been deposited in the github repository under accession code Giordano_Layer_Leonardelli_etal_CXCL12_GBM_GSE182109 (https://github.com/BaldLab).

Research involving human participants, their data, or biological material

Policy information about studies with human participants or human data. See also policy information about sex, gender (identity/presentation), and sexual orientation and race, ethnicity and racism.

Reporting on sex and gender	There were no restrictions regarding sex or gender of participating patients and male as well as female patients participated (see below). Sex was determined based on self-report. The guidance on Sex and Gender reporting was followed and corresponding statements are included in the resubmitted manuscript. Given the rather low sample size reported in this study, we feel a disaggregation for sex and gender does not contribute to the conclusions of this work. However, all information on individual outcomes of patients of all sexes are provided for the interested reader.
Reporting on race, ethnicity, or other socially relevant groupings	We do not report on race, ethnicity or other relevant groupings of participants. No such restrictions applied for inclusion
Population characteristics	All patients were first-diagnosed and neuropathologically confirmed as glioblastoma, IDH-wildtype (CNS WHO grade 4) according to the WHO classification for CNStumors 2021by immunohistochemistry (IHC).The median age at diagnosis was 65 years(range 43-79 years).Seven patients considered themselves male and three female. Seven had an ECOG score of 0, while three had an ECOG score of 1. Eight of ten patients had undergone partial resection, two were not amenable to resection and received biopsy only. Seven patients received normofractionated and three hypofractionated RT.
Recruitment	Inclusion criteria of the dose-escalation arm of the trial were age ≥ 18 years, incompletely resected or biopsied GBM (detectable postoperative residual tumor), absence of MGMT promoter (hyper)methylation, Eastern Cooperative Oncology Group(ECOG) performance score ≤2, estimated life expectancy ≥3 months, stable or decreasing dose of corticosteroids and adequate hepatic and renal function. Sex was determined based on self-report. All patients were neuropathologically confirmed as glioblastoma, IDH-wildtype (CNS WHO grade 4) according to the WHO classification for CNS tumors 2021 by immunohistochemistry (IHC). If patients were ≤ 54 years of age, they were assessed additionally by pyrosequencing for IDH1 and IDH2. GLORIA was conducted at six academic centers in Germany, whereas the protocol was approved by ethic committees at each participating site. Each patient provided written informed consent in accordance with established guidelines. No trial participant received financial compensation. If the inclusion criteria were fulfilled, patients were screened for recruitement by the individual study sites. Self-selection bias can be excluded, since patients only got to choose inclusion into the trial, there were no treatment arms to choose from. Selection bias cannot ultimately be fully excluded as investigators decised independently on fulfilment of inclusion criteria thus gating study entry. However inclusion criteria did not leave much room for interpretation and thus investigator-based preferences for individual patients. The distribution of age and resection status in this trial demonstrates that no preference towards fitter patients was apparent.
Ethics oversight	GLORIA was conducted at six academic centers in Germany, whereas the protocol was approved by ethic committees at each participating site (ethic committees of the university hospitals of Mannheim, Bonn, Leipzig, Essen, Tübingen and Münster). The study design and conduct complied with all relevant regulations regarding the use of human study participants. The trial followed the guidelines of the Declaration of Helsinki and the International Conference on Harmonization Good Clinical Practices Guidelines. Each patient provided written informed consent in accordance with established guidelines. The trial was reviewed by an independent data safety and monitoring committee. The sponsor agreed to the separate report of the dose escalation part of the trial as provided in this manuscript after the end of follow-up of the last patient of DL3, as all patients in the expansion arm receive differing treatment combinations impairing comparability. This dose escalation part was the only part of the trial in the initial protocol versions before the expansion arms were amended to explore additional combination treatment options of interest.

Note that full information on the approval of the study protocol must also be provided in the manuscript.

Field-specific reporting

Please select the one below that is the best fit for your research. If you are not sure, read the appropriate sections before making your selection.

- [x] Life sciences
- [ ] Behavioural & social sciences
- [ ] Ecological, evolutionary & environmental sciences

For a reference copy of the document with all sections, see nature.com/documents/nr-reporting-summary-flat.pdf

Life sciences study design

All studies must disclose on these points even when the disclosure is negative.

Sample size	No formal sample size calculations were performed for this standard dose-escalation trial. The dose escalation was designed as a 3+3 rule-based design according to Le Tourneau et al. with three successional cohorts consisting of three patients each. Patients of DL1 were to be treated with a weekly dose of 200 mg, of DL 2 with a weekly dose of 400 mg and of DL 3 with a weekly dose of 600 mg NOX-Al2. After four weeks of treatment of the first patient of DL1, the data safety monitoring board (DSMB) reviewed all DLTs, AEs, and relevant laboratory values. During the following ten weeks of treatment, the DSMB was kept informed continuously about all DLTs and SAEs, and, at the end of this period, reviewed all DLTs, AEs, and relevant laboratory values including NOX-Al2 plasma concentrations prior to enrolment of the next two patients of this DL. The evaluation was repeated prior to enrolling patients in DL 2 and after patients 2 and 3 received at least four weeks of treatment. The same procedures were performed prior to enrolment of further patients in DL 2 and for DL3. If none of the three patients in any DL experienced a DLT, another three patients were to be treated at the next higher DL. However, if one of the three patients in a DL experienced a DLT, three more patients were to be treated at the same DL. The dose escalation was planned to be continued until at least two patients among a cohort of three to six patients experienced DLT (i.e., ≥ 33% of patients with a DLT at that DL), but the dose would not be escalated beyond 600 mg/week. The recommended dose for phase II trials was defined as the DL just below this toxic dose level, or 600 mg/week if this DL is not toxic. As reported, a total of 10 patients was enrolled and no DLTs were observed, while treatment with RT and NOX-Al2 was safe and well tolerated.
Data exclusions	No data were excluded from the analysis.
Replication	Findings from the clinical part of the trial cannot be replicated technically, since they represent findings in individual humans with glioblastoma that were treated with experimental or standard of care treatment. Replication its wider sense is given by the fact that a total of n=10 patients were treated (n=5 EG12high and n=5 EG12low), providing thus a robustness of the findings present. mIF stainings have been conducted in a total of n=32 samples (biological replicates) with consistent findings.
Randomization	We report on a non-randomized multicentric phase I/II study of RT in combination with NOX-A12 in first-line partially resected or unresected GBM (CNS WHO grade 4) patients with unmethylated MGMT promoter. To benchmark tissue and outcome, we established a reference cohort of GBM patients treated outside of the study with SOC RT and temozolomide (TMZ) at the University Hospital Bonn between 2010 and 2023.
Blinding	No randomization took place (see above).Both investigators and patients were not blinded and aware that every GLORIA patient would receive the experimental treatment. Placebo treatment was not considered for this trial since it was considered unethical without SOC treatment and would come with a relevant patient burden, as NOX-A12 was applied by 24/7 i.v. infusions of the drug over a pump system. Of note, the independent central reader was blinded to all clinical aspects of the trial. Also, mIF was performed and analysed blinded to all clinical aspects of the trial and patient identities.

Reporting for specific materials, systems and methods

We require information from authors about some types of materials, experimental systems and methods used in many studies. Here, indicate whether each material, system or method listed is relevant to your study. If you are not sure if a list item applies to your research, read the appropriate section before selecting a response.

Materials & experimental systems

n/a	Involved in the study	
[ ]	[x]	Antibodies
[x]	[ ]	Eukaryotic cell lines
[x]	[ ]	Palaeontology and archaeology
[x]	[ ]	Animals and other organisms
[ ]	[x]	Clinical data
[x]	[ ]	Dual use research of concern
[x]	[ ]	Plants

Methods

n/a	Involved in the study	
[x]	[ ]	ChIP-seq
[x]	[ ]	Flow cytometry
[ ]	[x]	MRI-based neuroimaging

Antibodies

Antibodies used	Ki-67 0.01 mg/mL; BD Pharmingen™ Purified Mouse Anti-Ki-67; Clone B56; BD Biosciences; Cat. Number 556003; RRID AB_396287<br><br>SDF1/CXCL12 0.01 mg/mL; CXCL12 Monoclonal Antibody (79018); Clone 79018; Cat. Number Thermo Fisher MA5-23759; RRID AB_2608711

α-SMA 0.01 mg/mL; Alpha-Smooth Muscle Actin Monoclonal Antibody (1A4), eBioscience™; Clone 1A4; Thermo Fisher Cat. Number 14-9760-82; RRID AB_2572996

CD31 0.01 mg/mL Anti-CD31 antibody [EP3095] - BSA and Azide free; Clone EP3095; abcam Cat. Number ab226157; Rabbit Recombinant Monoclonal CD31 antibody. Carrier free.

GFAP 0.01 mg/mL; GFAP Monoclonal Antibody (2.2B10); Clone 2.2B10; Thermo Fisher Cat. Number 13-0300; RRID AB_2532994

CD68 0.005 mg/mL; Clone KP-1; BioLegend Cat. Number 916104; RRID AB_2616797

Validation

Ki-67 0.01 mg/mL; BD Pharmingen™ Purified Mouse Anti-Ki-67; Clone B56; BD Biosciences; Cat. Number 556003; RRID AB_396287
Relevant citations
Schürch CM, Bhate SS, Barlow GL et al. Coordinated Cellular Neighborhoods Orchestrate Antitumoral Immunity at the Colorectal Cancer Invasive Front. Cell. 2020 Sep 3;182(5):1341-1359.e19.
Phillips D, Schürch CM, Khodadoust MS et al. Highly Multiplexed Phenotyping of Immunoregulatory Proteins in the Tumor Microenvironment by CODEX Tissue Imaging. Front Immunol. 2021 May 19;12:687673.
Benson MJ, Elgueta R, Schpero W, et al. Distinction of the memory B cell response to cognate antigen versus bystander inflammatory signals. J Exp Med. 2009; 206(9):2013-2025.
Matthew M. Gubin, Ekaterina Esaulova, Jeffrey P. Ward,, et al. High-Dimensional Analysis Delineates Myeloid and Lymphoid Compartment Remodeling during Successful Immune-Checkpoint Cancer Therapy, Cell (2018)

SDF1/CXCL12 0.01 mg/mL; CXCL12 Monoclonal Antibody (79018); Clone 79018; Cat. Number Thermo Fisher MA5-23759; RRID AB_2608711
Relevant citations
Li J, Shu X, Xu J et al. S100A9-CXCL12 activation in BRCA1-mutant breast cancer promotes an immunosuppressive microenvironment associated with resistance to immunotherapy. Nat Commun. 2022 Mar 18;13(1):1481.
Giallongo C, Dulcamare I, Tibullo D, et al. CXCL12/CXCR4 axis supports mitochondrial trafficking in tumor myeloma microenvironment. Oncogenesis. 2022 Jan 21;11(1):6

α-SMA 0.01 mg/mL; Alpha-Smooth Muscle Actin Monoclonal Antibody (1A4), eBioscience™; Clone 1A4; Thermo Fisher Cat. Number 14-9760-82; RRID AB_2572996
Relevant citations
Wu JY, Yeager K, Tavakol DN, Morsink M et al. Directed differentiation of human iPSCs into mesenchymal lineages by optogenetic control of TGF-β signaling. Cell Rep. 2023 May 30;42(5):112509
Cords L, Engler S, Haberecker M, Rüschoff JH et al. Cancer-associated fibroblast phenotypes are associated with patient outcome in non-small cell lung cancer. Cancer Cell. 2024 Mar 11;42(3):396-412.e5.

CD31 0.01 mg/mL Anti-CD31 antibody [EP3095] - BSA and Azide free; Clone EP3095; abcam Cat. Number ab226157; Rabbit Recombinant Monoclonal CD31 antibody. Carrier free.
Validated in IHC-P, WB, Flow Cyt (Intra) and tested in Human samples (manufacturer´s website).
Relevant citations
Sparano JA, Gray R, Oktay MH, et al. A metastasis biomarker (MetaSite Breast™ Score) is associated with distant recurrence in hormone receptor-positive, HER2-negative early-stage breast cancer. NPJ Breast Cancer. 2017 Nov 8;3:42.
Liang W, Wu J, Qiu X. LINC01116 facilitates colorectal cancer cell proliferation and angiogenesis through targeting EZH2-regulated TPM1. J Transl Med. 2021 Jan 26;19(1):45.

GFAP 0.01 mg/mL; GFAP Monoclonal Antibody (2.2B10); Clone 2.2B10; Thermo Fisher Cat. Number 13-0300; RRID AB_2532994
Relevant citations
Schürch CM, Bhate SS, Barlow GL et al. Coordinated Cellular Neighborhoods Orchestrate Antitumoral Immunity at the Colorectal Cancer Invasive Front. Cell. 2020 Sep 3;182(5):1341-1359.e19.
Wei H, Wu X, Withrow J, et al.. Glial progenitor heterogeneity and key regulators revealed by single-cell RNA sequencing provide insight to regeneration in spinal cord injury. Cell Rep. 2023 May 30;42(5):112486.

CD68 0.005 mg/mL; Clone KP-1; BioLegend Cat. Number 916104; RRID AB_2616797
IHC-P - Quality tested (manufacturer´s website).
Relevant citations
Schürch CM, Bhate SS, Barlow GL et al. Coordinated Cellular Neighborhoods Orchestrate Antitumoral Immunity at the Colorectal Cancer Invasive Front. Cell. 2020 Sep 3;182(5):1341-1359.e19.
Phillips D, Schürch CM, Khodadoust MS et al. Highly Multiplexed Phenotyping of Immunoregulatory Proteins in the Tumor Microenvironment by CODEX Tissue Imaging. Front Immunol. 2021 May 19;12:687673.
Wagner J, Rapsomaniki MA, Chevrier S et al.. A Single-Cell Atlas of the Tumor and Immune Ecosystem of Human Breast Cancer. Cell. 2019 May 16;177(5):1330-1345.e18.

Clinical data

Policy information about clinical studies

All manuscripts should comply with the ICMJEguidelines for publication of clinical research and a completedCONSORT checklist must be included with all submissions.

Clinical trial registration	EudraCT: 2018-004064-62; ClinicalTrials.gov: NCT04121455
Study protocol	Besides the information provided in the official trial registration pages listed above, the trial protocol is provided alongside this publication.
Data collection	Following adequate cranial wound healing and implantation of a venous port catheter, treatment with NOX-A12 was initiated witsequences. Between September 2019 and September 2021, a total of 10 patients was enrolled and treated. Follow-up of the last patient ended in August 2022.hin six weeks post cranial surgery. After an initial dose of 70, 160 or 230 mg per day respectively on day 1, patients were administered a fixed dose of 200, 400 or 600 mg NOX-A12 per week (DL 1, DL 2, DL 3) by continuous (24h) i.v. infusion over a commercially-available closed pump system (CADD®-Solis VIP Ambulatory Infusion Pump by Smiths Medical) starting on day 1. Treatment with NOX-A12 ended after 26 weeks. Patients with disease progression during the 26-week treatment period continued treatment with all assessments if deemed appropriate by the investigator. Continuation of treatment with NOX-A12 beyond 26 weeks was allowed as per each investigator's decision, if the patient had clear clinical benefit. No simultaneous systemic oncologic treatment was permitted. Clinical and radiographic follow-up assessments included standard and advanced magnetic resonance imaging (MRI) sequences. Between September 2019 and September 2021, a total of 10 patients was enrolled and treated. Follow-up of the last patient ended in August 2022.
Outcomes	The primary endpoint of the trial was safety as per the incidence of AEs. Secondary endpoints included NOX-A12 plasma levels, imaging parameters with a specific emphasis on monitoring re-vascularization, PFS, OS and clinician/patient reported outcomes (CRO/PRO). As an additional exploratory endpoint, tumor tissue obtained in surgery was analyzed by mIF staining (CODEX®).

Plants

Seed stocks	NA
Novel plant genotypes	NA
Authentication	NA

Magnetic resonance imaging

Experimental design

Design type	NA
Design specifications	NA
Behavioral performance measures	NA

Acquisition

Imaging type(s)	structural, diffusion, perfusion - no fMRI, no psychological testing
Field strength	not defined
Sequence & imaging parameters	MRI imaging sequences included: 3D Tl-weighted volumetric imaging (3D T1), T2-fluid-attenuated inversion recovery (FLAIR) imaging, diffusion-weighted imaging (DWI), Tl-weighted dynamic contrast-enhanced perfusion imaging (DCE), T2-weighted turbo spin-echo imaging (T2 TSE), T2-weighted dynamic susceptibility contrast-enhanced perfusion imaging (DSC), and post-contrast 3D T1 imaging. The following additional advanced imaging parameters were calculated: diffusion-weighted imaging (DWI)-derived ADC; diffusion susceptibility contrast (DSC)-derived leakage- corrected normalized rCBV and threshold-calculated FTBhigh (rCBV > 1.75); dynamic contrast-enhanced (DCE)-derived transfer constant of contrast agent (Ktrans) between the blood and the extravascular extracellular space (EES), fractional EESvolume (ve), and fractional plasma volume (vp).
Area of acquisition	wholebrain

Diffusion MRI ☒ Used ☐ Not used

Parameters: as per radiologist's discretion with consecutive central quality check

Preprocessing

Preprocessing software	Following acquisition, MRI images were uploaded to a secure online portal (decidemedical, Clinflows) where a central quality check was performed. All image post-processing and interpretation was performed using IB NeuroTM (Imaging Biometrics), Olea Sphere (Olea Medical) and Mint LesionTM (Mint Medical GmbH) software and assessed by a central reader not involved in the treatment of the patients (SB).
Normalization	NA
Normalization template	NA
Noise and artifact removal	NA
Volume censoring	NA

Statistical modeling & inference

Model type and settings	NA
Effect(s) tested	NA

Specify type of analysis: ☐ Whole brain ☐ ROI-based ☒ Both

Anatomical location(s): All MRI images were uploaded to an imaging database and outcome was centrally assessed by a board-certified radiologist with expertise in the field blinded for study site and clinical status. Target (TL) and non-target lesions (NTL) were identified, validated and assessed in regard to tumor size (SPD) and corresponding timepoint tumor response according to the modified Criteria for Radiographic Response (mRANO). For details, see reference 53. One patient enrolled had a singular residual tumor lesion meeting the inclusion criteria, while not qualifying for a target lesion (<10 mm in at least one diameter as per mRANO), thus documented as NTL. New non-measurable contrast-enhancing lesions only constituted progression in case of complete response (CR). NTLs only impacted the response assessment in the case of a complete response of TLs. Preliminary tumor progression

Statistic type for inference (See Eklund et al. 2016)	NA
Correction	NA

Models & analysis

n/a	Involved in the study
☒	☐ Functional and/or effective connectivity
☒	☐ Graph analysis
☐	☒ Multivariate modeling or predictive analysis

Multivariate modeling and predictive analysis: Survival rates were estimated using the Kaplan-Meier method and statistically assessed by log-rank test and Cox proportional hazards regression. For Cox proportional hazards regression, a time to event variable (PFS or OS) was analyzed in regard to the event variable (censored or event) for two patient cohorts providing a hazard ratio, its 95% confidence interval and a p-value.
