# Supplementary material for: L-RNA aptamer-based CXCL12 inhibition combined with radiotherapy in newly-diagnosed glioblastoma: dose escalation of the phase I/II GLORIA trial
Source: Nat Commun. 2024 May 28;15:4210. doi: 10.1038/s41467-024-48416-9 (PMC11133480; doi:10.1038/s41467-024-48416-9)
Supplement: Supplementary file 3 — Description of Additional Supplementary Files [file 41467_2024_48416_MOESM3_ESM.pdf]

Supplementary Data 1:
Complete listing of adverse events.

Supplementary Data 2:
Complete listing of treatment-emergent adverse events per dose level.

Supplementary Data 3:
Complete listing of treatment-emergent adverse events related to underlying disease and/or irradiation per dose level.

Supplementary Data 4:
Combined lists of baseline data and calculations. First tab: Clinical baseline data, mIF-derived quantification of cell phenotypes and calculation of EG12 scores. Second tab: MRI imaging results. Third tab: Plasma levels. Fourth tab: NANO scores.

Supplementary Data 5:
Multi-cycle reactions protocol and list of oligonucleotid sequences used for mIF.
